# Engineered PN MoS_2_–Al_2_O_3_-Based Photodiode Device for High-Performance NIR LiDAR and Sensing Applications

**DOI:** 10.3390/s26020542

**Published:** 2026-01-13

**Authors:** Ahmed Abdelhady A. Khalil, Abdallah M. Karmalawi, Moamen R. A. Elsayed, Ramy El-Bashar, Hamdy Abdelhamid, Heba A. Shawkey, S. S. A. Obayya, Mohamed Farhat O. Hameed

**Affiliations:** 1 National Institute of Laser Enhanced Sciences (NILES), Cairo University, Giza 12613, Egypt; 2 Center for Nanotechnology, Zewail City of Science, Technology and Innovation, October Gardens, 6th of October City, Giza 12578, Egypt; mfarahat@zewailcity.edu.eg; 3 Radiometry Metrology Laboratory, Division of Photometry and Radiometry, National Institute of Standards (NIS), President Sadat St., 136, Al-Haram, Giza 12211, Egyptmoamen.ragab@nis.sci.eg (M.R.A.E.); 4 Electrical Engineering Department, College of Engineering and Information Technology, Ajman University, Ajman P.O. Box 346, United Arab Emirates; 5 Centre for Nanoelectronics and Devices (CND), Zewail City of Science, Technology and Innovation, October Gardens, 6th of October City, Giza 12578, Egypt; 6 Microelectronics Department, Electronics Research Institute (ERI), Cairo 12622, Egypt; 7 Centre for Photonics and Smart Materials, Zewail City of Science, Technology and Innovation, October Gardens, 6th of October City, Giza 12578, Egypt; 8 Faculty of Engineering, University of Mansoura, Mansoura 35516, Egypt

**Keywords:** photodetector, PN photodiode, MoS_2_–Al_2_O_3_ photodiode, light harvesting device, photodetector performance parameters, metrology

## Abstract

Near-infrared (NIR) photodetectors are essential for LiDAR, optical communication, and sensing technologies requiring fast response and low power consumption. This work reports a PN photodiode incorporating a co-sputtered MoS_2_–Al_2_O_3_ composite layer to enhance NIR photoresponse for LiDAR and environmental sensing applications. The composite layer improves device performance through defect passivation, dielectric screening, and modified carrier transport behavior. Under 100 mW·cm^−2^ illumination at 4 V, the device delivers a photocurrent of 10 mA with a response time of 155 µs, corresponding to an approximately threefold (~300%) improvement compared to a reference structure. Spectral measurements show peak responsivity at 970 nm with extended sensitivity up to 1100 nm. These results indicate that embedding Al_2_O_3_ within the MoS_2_ improves the MoS_2_/Si interface and facilitates infrared photon absorption in the Si substrate, leading to enhanced vertical carrier collection and reduced recombination compared with conventional surface-passivated MoS_2_/dielectric layers-based devices. The proposed device demonstrates a low-cost, broadband photodiode architecture suitable for eye-safe LiDAR and environmental monitoring applications.

## 1. Introduction

Transition metal dichalcogenides (TMDCs) [1] have revolutionized nanotechnology due to their exceptional optical and electrical properties. Molybdenum disulfide (MoS_2_) [2] is a well-studied TMDC due to its unique bandgap characteristics. MoS_2_ is an ideal material for transistors, photodetectors, and photovoltaic devices, with a direct bandgap of 1.8 eV in monolayer and an indirect bandgap of 1.2 eV in bulk. Despite their promising features, MoS_2_-based photodetectors have significant hurdles, including high sensitivity to external conditions and intrinsic defects, such as sulfur vacancies, which induce trap states and impair charge transport pathways [3,4]. The scientific community has adopted several creative tactics, such as sophisticated surface passivation and defect engineering methods, to reduce these restrictions [5]. The interface passivation in improving semiconductor performance has been previously confirmed in perovskites [6,7] and nanowire-based solar cells [8], where passivation layers can suppress recombination losses. A strategy similarly employed in the MoS_2_–Al_2_O_3_ photodiode to enable high responsivity and broadband detection. For example, recent studies have demonstrated that the responsivity and operational stability of MoS_2_ photodetectors can be greatly improved by applying hydrogen plasma treatment in combination with layered passivation employing aluminum oxide (Al_2_O_3_) and hafnium oxide (HfO_2_) [9]. Notably, a responsivity of 567 A/W was achieved, with a rise time of 10.51 s and a decay time of 15.78 s at a bias of −5 V. The devices retained approximately 95% of their performance metrics after prolonged exposure to ambient conditions [9]. Al_2_O_3_ is frequently employed in semiconductor fabrication due to its outstanding insulating properties and strength against chemical degradation [10]. When integrated with MoS_2_ photodetectors, Al_2_O_3_ not only provides environmental protection but also induces tensile strain that modulates MoS_2_’s electronic structure and enhances carrier mobility [11]. A thin (~3 nm) ALD-grown Al_2_O_3_ layer has been reported to increase photocurrent and responsivity by an order of magnitude through strain engineering and defect passivation [12]. Latest breakthroughs in MoS_2_ photodetectors have yielded remarkable advancements [13]. Recent advances have reported significant performance enhancements, including responsivities reaching 16.1 A/W with noise-equivalent power (NEP) values as low as 8 × 10^−15^ W Hz^−1/2^ based on Al_2_O_3_ stress liners to enhance carrier mobility [14]. Further, higher responsivities, exceeding 500 A/W, have been introduced in devices employing hydrogen plasma treatment combined with alternating Al_2_O_3_/HfO_2_ passivation stacks [15]. Complementary approaches—such as hybrid sensitization, van der Waals (vdW) heterostructures, and flexible substrates—have expanded MoS_2_ photodetection into broadband, self-powered, and ultrafast regimes [16,17,18,19].

Recent work by Iqbal et al. [20] has shown that MoS_2_–Al_2_O_3_ composite films prepared by reactive co-sputtering exhibit systematically enhanced absorption and modified optical constants relative to pure MoS_2_. Building on this concept, the present study introduces a homogeneous MoS_2_–Al_2_O_3_ composite photodiode architecture in which both materials are intimately intermixed rather than stacked. This distributed junction geometry facilitates improved charge transport, enhanced dielectric screening, and effective defect passivation. The composite was synthesized by RF sputtering and comprehensively characterized using ATR, Raman, SEM/EDX, XRD, and AFM. Electrical measurements reveal a strong increase in photocurrent with illumination intensity, demonstrating enhanced photosensitivity. The device achieves a photocurrent of ~10 mA under 1000 W m^−2^ irradiation and a fast rise time of 155 µs, representing a substantial improvement over many MoS_2_ photodetectors whose response times commonly extend from milliseconds to several seconds [21,22]. Compared with the baseline MoS_2_ photodiode of Khalil et al. [23], the composite design delivers a threefold increase in photocurrent and substantially extended spectral sensitivity. These enhancements arise from Al_2_O_3_-induced defect passivation, dielectric screening, and mild tensile strain, which collectively suppress recombination, extend carrier diffusion length, and redshift absorption toward the 970 nm region, leading to stronger IR coupling and improved charge transport. Overall, the MoS_2_–Al_2_O_3_ composite photodiode demonstrates high sensitivity, fast response, and broad spectral performance, underscoring its potential for advanced optical sensing, imaging, and communication applications.

Beyond its optoelectronic merits, the Al_2_O_3_/MoS_2_ composite photodiode also presents a promising potential for environmental and near-infrared (NIR) LiDAR applications. Eye-safe NIR detection within the 850–1000 nm range is increasingly crucial for remote sensing, forestry canopy profiling, and atmospheric or topographic mapping, where high responsivity, low dark current, and stable operation under low-light conditions are required [24,25,26]. The enhanced broadband sensitivity and suppressed recombination losses enabled by the Al_2_O_3_ interfacial layer directly address these system-level demands. By combining CMOS-compatible fabrication with strong NIR responsivity, the proposed MoS_2_–Al_2_O_3_ architecture offers a cost-effective, scalable alternative to conventional InGaAs or Si-based photodiodes typically used in slow-scan and environmental LiDAR receivers [27,28]. This makes it particularly suitable for long-range, eye-safe sensing platforms emphasizing signal integrity and energy efficiency over ultrafast temporal response.

## 2. Device Fabrication, Characterization, and Dynamic Performance Parameters

### 2.1. Device Fabrication: Coating Procedure

**Figure 1:**
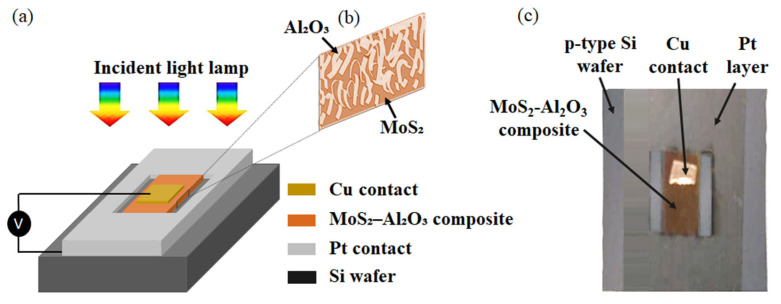
(**a**) Schematic diagram and (**b**) the corresponding fabricated photodiode based on MoS_2_–Al_2_O_3_ composite (**c**) photograph of the fabricated device under test (DUT).

The photodiode devices are fabricated using a physical vapor deposition (PVD) technique via radio frequency (RF) sputtering, using a PROTOFLEX 1400 system (IBM, Inc., Armonk, NY, USA) at the Egyptian Nanotechnology Center, Cairo University. The van der Waals p–n junction is realized by vertically stacking a composite semiconductor TF-comprising a monolayer blend of Al_2_O_3_ and n-type MoS_2_ onto a p-type silicon (Si) wafer substrate. Figure 1a illustrates the layered architecture of the fabricated photodiode. The cross-sectional side view of the MoS_2_–Al_2_O_3_ composite layer is visualized in Figure 1b. A top view of the actual fabricated device structure is presented in Figure 1c. This work used a single-sided polished, ⟨100⟩-oriented silicon wafer with a thickness of 675 µm (Prime Grade) [29]. The active composite layer of molybdenum disulfide (MoS_2_) and aluminum oxide (Al_2_O_3_) was deposited on the Si wafer using RF magnetron sputtering. High-purity MoS_2_ (4 mm, 99.9999%) and Al_2_O_3_ (8 mm, 99.9999%) targets were used, with ultra-high-purity argon as the sputtering gas. During deposition, the substrates were held at room temperature with no intentional heating or cooling to preserve the materials’ intrinsic characteristics and avoid undesired phase transitions. To reduce contamination, the process was carried out in an ultra-high vacuum (UHV) chamber evacuated by a cryogenic pump. Sputtering parameters were extensively tuned to produce homogeneous thin films with exact thickness control. The deposition was performed at an argon working pressure of 5 × 10^−2^ Torr, a substrate temperature of 25 °C, a gas flow rate of 30 sccm, and an RF power of 150 W. The chamber’s base pressure was set to 10^−5^ Torr, while the deposition pressure remained at 10^−3^ Torr. In this study, MATLAB 2024 is utilized to redraw and visualize the data presented in the figures.

A Cu stripe is used atop the active layer to serve as the cathode, as illustrated in Figure 1a. Additionally, thin platinum (Pt) layer is patterned to frame the p-type Si region, which is itself sputtered in a ring-like geometry to encapsulate the MoS_2_–Al_2_O_3_ composite and serve as the anode. This Pt contact plays a crucial role in facilitating charge transport and enhancing device performance [30]. Its presence mitigates recombination losses and leads to a substantial increase in photocurrent [31], and demonstrates superior performance compared to conventional oxide-contacted photodiodes [32]. The strategic use of Pt—known for its high work function and excellent conductivity [33]—further contributes to efficient carrier collection and improves the photodetection capabilities [34]. The active areas of the fabricated TFs are defined with high precision to ensure reproducibility and device performance optimization. In this work, the selected dimensions from the bottom are as follows: p-type Si wafer (1.5 × 1.5 cm^2^), Pt thin electrode (outer dimension 1.5 × 1.5 cm^2^, inner active window 0.6 × 1 cm^2^), MoS_2_–Al_2_O_3_ composite TF (0.6 × 0.8 cm^2^), and Cu electrode TF (0.2 × 0.2 cm^2^). The adjustment of the Pt electrode dimensions ensures that the semiconductor active area is precisely confined to 1 × 1 cm^2^. Based on prior experience, these dimensions were deliberately chosen to maximize light absorption and carrier extraction, thereby ensuring an optimal balance between device area and responsivity. In this work, all reported performance data were obtained from a single fabricated photodetector device that was fully characterized for its optoelectronic response. To reinforce confidence in the results, several additional devices were fabricated under identical conditions and systematically employed for complementary analyses, including detailed morphological characterization and dynamic performance measurements of the DUT. The agreement observed across these identically prepared devices demonstrates that the reported characteristics are reproducible outcomes of the fabrication process rather than isolated or exceptional results. Taken together, these findings confirm the reliability, reproducibility, and representative nature of the presented device performance.

### 2.2. Morphological, Structural, and Spectroscopic Characterization of the MoS_2_–Al_2_O_3_ Active Layer

To elucidate the structural, morphological, and vibrational properties of the co-sputtered MoS_2_–Al_2_O_3_ composite active layer, a suite of advanced characterization techniques was employed as detailed below.

*a*  *Fourier Transform Infrared Spectroscopy (FTIR: ATR)*

Chemical bonding and functional group analysis were conducted using a Thermo Nicolet 6700 FTIR spectrometer (Thermo Fisher Scientific (formerly Thermo Nicolet), Madison, WI, USA) mode is used to measure chemical fingerprints of functional groups in the 400–4000 cm^−1^ spectral range. This technique enables precise identification of both organic and inorganic constituents through vibrational fingerprinting. Key advantages of FTIR include non-destructive analysis, high spectral resolution, and broad applicability across diverse material systems.

*b*  *Raman Spectroscopy Characterization*

Raman spectroscopy was also employed to evaluate the structural and vibrational characteristics of the co-sputtered MoS_2_–Al_2_O_3_ composite active layer. Measurements were performed using a HORIBA Scientific Raman spectrometer operated via LabSpec 6 software, configured with a 532 nm edge-filtered excitation laser, a 1800 grooves/mm grating (visible range), a ×50 WS UWD objective lens, and a slit width of 48 µm. Spectra were acquired over the 0–3500 cm^−1^ range with a 10 s accumulation time and ICS correction enabled.

*c*  *Scanning Electron Microscope*

High-resolution morphological analysis using a Thermo Fisher Scientific Quanta FEG 250 field-emission scanning electron microscope (FE-SEM) operating at 5–20 kV accelerating voltage and equipped with energy-dispersive X-ray spectroscopy (EDS) for simultaneous compositional analysis, is also used for field-emission environmental scanning electron microscopy (FE-ESEM) and elemental mapping, enabling detailed visualization of surface topology, grain distribution, and film uniformity across the composite layer.

*d*  *Elemental Mapping by Energy-Dispersive X-Ray Spectroscopy*

Energy-dispersive X-ray (EDX) mapping was used to obtain spatially resolved compositional information across the MoS_2_–Al_2_O_3_ thin film. In this technique, the specimen is irradiated with a focused electron beam, which induces the emission of characteristic X-rays from the constituent elements. These X-rays are detected and processed to generate elemental distribution maps, allowing visualization of the spatial arrangement of specific atoms within the scanned region. When integrated with scanning electron microscopy, EDX-mapping provides a powerful means of correlating morphological features with compositional data, thereby enabling comprehensive characterization of thin-film uniformity and microstructural integrity.

*e*  *X-ray diffraction (XRD)*

Phase composition and crystallographic structure were investigated via wide-angle X-ray diffraction using a Bruker D8 diffractometer equipped with small-angle capability. This technique provided insights into lattice parameters, crystallite size, and preferred orientation within the composite matrix.

*f*  *Surface Roughness*

Topographical and nanoscale surface features were characterized using an Agilent Technologies 5600LS AFM system. Capable of resolving atomic-scale features on both conductive and non-conductive substrates, AFM offered three-dimensional imaging with minimal sample preparation, thereby enabling accurate quantification of surface roughness and texture. The structural, morphological, and spectroscopic characterizations, including X-ray diffraction (XRD), Raman spectroscopy, atomic force microscopy (AFM), and Fourier transform infrared spectroscopy (FTIR), were carried out at the Faculty of Nanotechnology, Cairo University. High-resolution imaging via Scanning Electron Microscopy (SEM) and EDX-Mapping was conducted at the Electron Microscopy Unit of the National Research Center (NRC).

### 2.3. Dynamic Performance Parameters of the Fabricated DUT

To evaluate the functional efficacy of the fabricated device under test (DUT), a series of dynamic electrical and optoelectronic measurements was conducted. These assessments aimed to quantify key performance metrics, including current–voltage behavior, photoresponse characteristics, and switching dynamics under controlled biasing and illumination conditions. The following subsections detail the methodologies, instrumentation, and observed trends associated with each parameter, providing insight into the operational stability and responsiveness of the MoS_2_–Al_2_O_3_-based active layer within the device architecture.

*a*  *I–V characteristics setup*

In this study, the current–voltage (I–V) behavior of the photodiode is characterized by sweeping the applied load voltage at different calibrated illumination intensities of 130 W/m^2^, 500 W/m^2^, 800 W/m^2^, and 1000 W/m^2^. During these measurements, the studied device was maintained at a constant ambient temperature of 25 °C. To measure the incident irradiance during testing, a traceably calibrated SMP22 pyranometer (Kipp & Zonen) was employed, ensuring accuracy and conformity with international metrological standards. The sensor exhibits a broad spectral response spanning from 285 nm to 2800 nm. A steady-state direct current (DC) bias was applied to the device under test (DUT) throughout the measurement sequence, while the I–V electrical characteristics were performed using a pair of calibrated Keithley 2010 precision multimeters. One of them is configured to measure the potential difference across the DUT, whereas the other monitors the voltage drop across a calibrated 100 Ω series-connected resistor, thereby enabling high-fidelity current extraction [35,36]. The I–V characteristics of the samples were measured using a Quartz Tungsten Halogen (QTH) lamp under dark conditions and at varying irradiance levels (130, 500, 800, and 1000 W/m^2^). In this work, the active area of the device is about (~1 cm^2^) is uniformly exposed to illumination, while the applied bias voltage is linearly varied from −4 V to +4 V in 1 mV steps using a programmable DC source meter. Instrument control and data acquisition are fully automated through a LabVIEW-based interface. A comprehensive description of the I–V measurement system can be found in references [37,38].

*b*  *System Configuration for Relative Spectral Responsivity Characterization*

Spectral responsivity (SR) characterizes the wavelength-dependent response of a photodetector, quantifying its ability to convert incident optical power at a given wavelength into an electrical output, typically a photocurrent. Under monochromatic illumination conditions, SR is evaluated by comparing the photocurrent generated by the DUT to that of a calibrated reference photodetector (model S2281, Hamamatsu) at −2v biasing voltage. The spectral responsivity of the DUT, denoted as $R_{DUT}(\lambda )$, at specific wavelengths is determined using a comparative measurement approach, as detailed in references [23,39,40,41]. This relationship is mathematically expressed as:

(1) $$R_{DUT}\left(\lambda \right)=\frac{I_{DUT}\left(\lambda \right)}{I_{REF}\left(\lambda \right)}\times R_{REF}\left(\lambda \right)\times G$$

where *I_DUT_* (*λ*) and *I_REF_* (*λ*) represent the photocurrent signals of the DUT and the reference detector, respectively, $R_{REF}(\lambda )$ denotes the known spectral responsivity (A/W) of the reference detector, and G is the transimpedance amplifier gain, which is unity in this setup. All measurements were conducted at the National Institute for Standards (NIS) under controlled thermal conditions, maintaining the DUT @ 25.0 ± 1.0 °C. Details of the quantum efficiency (QE) measurement system are provided in Ref. [42]; however, QE data are not presented in this work, as the responsivity measurements discussed herein are relative, and QE—being an absolute metric—would not offer the intended comparative insight.

Quantum efficiency (QE) measures how effectively incident radiant energy induces a measurable response—such as photocurrent—in a photosensitive device [43]. It is categorized into internal QE (IQE), referring to the ratio of generated carriers to absorbed photons, and external QE (EQE), which relates to the ratio of generated carriers to all incident photons. QE is typically expressed as a function of wavelength or absorption coefficient and ideally reaches unity when all photons are absorbed and collected. In practice, QE < 1 due to losses from reflection, recombination, structural defects, passivation layer variations, and doping effects [44]. As a result, upon illumination, the incident radiant flux on a photodiode is separated into three components: reflectance, absorbance, and transmittance. Each of these components makes a unique contribution to the device’s total optical response. EQE ($\eta_{e}$) is a fundamental metric for assessing photodetector performance, and its quantitative evaluation can be related to the optical input to the resulting electrical output as follows [43,45,46]:

(2) $$\eta_{e}=\frac{R\left(\lambda \right)hc}{\lambda e}$$

where R(λ) represents the responsivity, *h* is Planck’s constant, λ is the wavelength, e is the electronic charge, and *c* is the light speed. The relative EQE derived from measured responsivity effectively maps the spectral photon-to-electron conversion efficiency. On the other hand, IQE quantifies how effectively absorbed photons are converted into usable electrical signals, excluding losses due to reflection or transmission. Moreover, IQE is distinct from external quantum efficiency (EQE), which excludes the optical-based losses. If there is no light transmitted, the $IQE\left(\eta_{i}\right)$ can be obtained directly from the photodiode reflectance (*R*) [45]:

(3) $$\eta_{i}=\frac{\eta_{e}}{1-R\left(\lambda \right)}$$

*c*  *Photo-transient response time system*

The experimental setup for evaluating the response time is configured to characterize the temporal behavior of the PN photodiode by ensuring a well-defined unidirectional current flow. Fast photoresponse is a key performance metric for assessing the efficiency of photodetectors; therefore, a mechanical optical chopper operating at a modulation frequency of 25 Hz is employed to modulate the incident light. The photo-transient response measurement procedure follows established methodologies described in references [36,38,39,40]. All measurements are performed under a constant bias voltage of 4 V, with transient photocurrent signals from both the test photodiode and a calibrated reference detector recorded simultaneously using a GDS-2202 digital storage oscilloscope (200 MHz bandwidth).

## 3. Results and Discussion

### 3.1. Characterization Results

To comprehensively evaluate the structural, chemical, and morphological attributes of the newly developed MoS_2_–Al_2_O_3_ composite-based photodiode, a multi-technique characterization scheme was employed. Attenuated Total Reflectance (ATR) spectroscopy was used to identify characteristic absorption bands associated with functional groups, confirming the chemical composition of the hybrid film. Raman spectroscopy validated the successful deposition of MoS_2_ by revealing its distinctive vibrational modes and providing insight into crystallinity and phase purity. Scanning Electron Microscopy (SEM) offered high-resolution imaging of the surface morphology, enabling assessment of film uniformity, grain dispersion, and topographical features. Elemental composition and spatial distribution of Mo, S, Al, and O were further examined through Energy-Dispersive X-ray (EDX) mapping, confirming the homogeneity of the composite. X-ray Diffraction (XRD) analysis was conducted to investigate the crystallographic structure and phase composition of the thin film. Additionally, Atomic Force Microscopy (AFM) was employed to quantify surface roughness and nanoscale texture, offering three-dimensional insight into the film’s interface quality.

*a*  *ATR characterization analysis*

**Figure 2:**
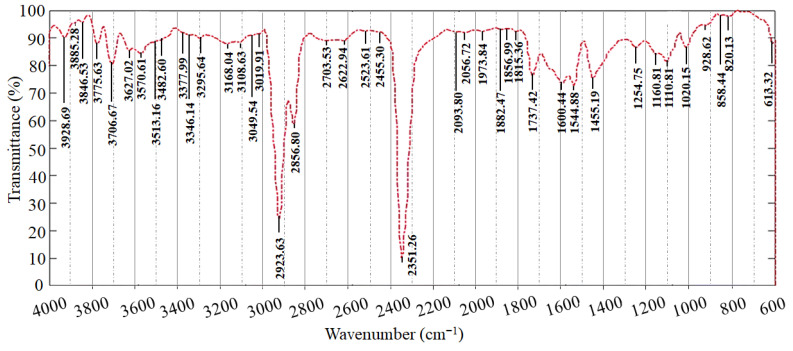
ATR chart for the MoS_2_–Al_2_O_3_ composite semiconductor layer.

Efficient deposition of MoS_2_–Al_2_O_3_ composite layer on a silicon substrate is demonstrated by the ATR spectrum in Figure 2. The observed vibrational characteristics reveal information about the structural states and interfacial interactions of both materials since they match their characteristic modes. The Al–O stretching vibration, a characteristic of crystalline α-Al_2_O_3_, is responsible for the pronounced peak at about 613.32 cm^−1^ with a high intensity (~90 units) [47,48]. This peak’s spectrum sharpness and stability suggest the creation of a well-crystallized alumina phase, which is in line with earlier research showing that α-Al_2_O_3_ formation with unique vibrational signatures is favored by calcination at high temperatures [49]. The crystalline nature of the phase affects its dielectric behavior and passivation characteristics, both of which are crucial for electronic applications. Additionally, the E_2_g (in-plane) and A_1_g (out-of-plane) vibrational modes of MoS_2_ are attributed to the peaks at 820.13 cm^−1^ and 858.44 cm^−1^ [50,51]. Small deviations from their usual locations point to substrate interactions, interfacial strain, or layer thickness effects, which are in line with research showing that few-layer MoS_2_ has different vibrational frequencies than bulk [50,51]. The presence of few-layer MoS_2_, which is known to improve electrical and optical characteristics, is confirmed by these vibrational features.

Moreover, Figure 2 also shows that there is a broad peak near 1020.15 cm^−1^. Such a peak corresponds to Si–O stretching vibrations originating from native silicon oxide on the substrate surface [52]. The minor peaks at approximately 1110.81 cm^−1^ are indicative of residual surface hydroxyl groups or physisorbed water molecules, common in surface-sensitive ATR spectra [53,54]. The overtones observed at 2351.3 cm^−1^ and 2856.8–2923.6 cm^−1^ are associated with multi-phonon or overtone vibrational modes of MoS_2_ and alumina, respectively, further confirming the composite’s vibrational complexity [55]. The Summary of key vibrational modes is shown in Table 1. Furthermore, the slight shifts and peak widths indicate potential interfacial interactions, such as strain transfer or chemical bonding, which can affect the composite’s electrical and optical properties [55]. The excellent spectral clarity and lack of superfluous characteristics indicate good material purity and low contamination, which is critical for reliable device operation.

*b*  *Raman characterization analysis*

**Figure 3:**
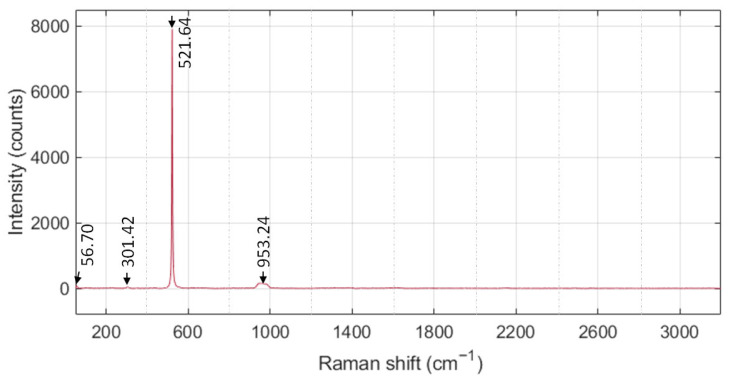
Raman chart for the MoS_2_–Al_2_O_3_ composite TF semiconductor layer.

Figure 3 shows the Raman spectrum of the co-sputtered MoS_2_–Al_2_O_3_ composite, which reveals a structurally unified film with vibrational features attributable to both constituents. A distinct low-frequency peak at 56.7 cm^−1^ is observed, corresponding to interlayer shear vibrations in few-layer MoS_2_. The persistence of this mode within the composite confirms that layered MoS_2_ domains are preserved while being structurally integrated with Al_2_O_3_, reflecting internal coupling rather than a discrete heterointerface. A prominent peak at 301.42 cm^−1^ is observed, which deviates from the canonical E mode of crystalline MoS_2_ and instead reflects a strain-modified or defect-influenced phonon associated with disordered few-layer MoS_2_. This shift is consistent with sputtered films exhibiting short-range order and lattice distortion [56]. Additionally, a peak at 521.64 cm^−1^ is attributed to Al–O vibrational modes, confirming the presence of Al_2_O_3_ within the composite. A weak and broad signal near 953.24 cm^−1^ is observed, which deviates from the sharp Raman activity typically associated with crystalline Al_2_O_3_ near 963 cm^−1^. This spectral behavior supports the presence of MoS_2_ in an amorphous phase, structurally embedded within the Al_2_O_3_ matrix [57,58]. This embedding serves a dual function: suppressing intrinsic MoS_2_ defects and modulating its electronic behavior. Effectively, Al_2_O_3_ acts as a p-type dopant within the n-type MoS_2_ framework [59,60]. Recent studies have further demonstrated that Al_2_O_3_ passivation layers can enhance MoS_2_ photodetector performance by improving stability and inducing favorable doping effects [4]. The resulting phonon shifts and strain signatures reflect interfacial coupling and charge redistribution [61], supporting enhanced internal field strength and improved carrier dynamics. Collectively, the spectral profile validates the composite’s integrity and its role in achieving superior optoelectronic performance.

*c*  *SEM characterization analysis*

The SEM characterization was also implemented as shown in Figure S1a, a side view of the manufactured structure was presented, while Figure S1b, shown in the Supplementary File, displays SEM investigation of the composite layer, which reveals a uniformly deposited, single-layer film made of MoS_2_ and Al_2_O_3_ nanosheets. Furthermore, cross-sectional SEM imaging in Figure S2b shows that this composite has a constant thickness ranging from 62.95 nm to 71.94 nm, indicating perfect control over the deposition process and ensuring conformality across the substrate surface (Figure S1a). The integrity of the film is further confirmed by the absence of pinholes or discontinuities in the SEM images, which is critically important for surface charge transport phenomena, as pinhole-free coverage prevents charge leakage pathways that could compromise device performance [62]. The smooth, pinhole-free morphology suggests a dense and continuous film, which is essential for reliable electrical conduction and mechanical stability in applications such as sensors or electronic devices [63]. Encapsulating this composite layer, a platinum (Pt) coating with a thickness ranging from 44.97 nm to 63.59 nm is observed, as shown in Figure S2a, shown in the Supplementary File, exhibiting uniform coverage without signs of agglomeration or defects as shown by Figure S1a. Controlled deposition of Pt in a surrounding frame configuration result in a stable and conductive boundary. This boundary contributes to electrical contact quality and limits chemical exposure at the composite interface [64]. The narrow thickness variation observed across the semiconducting layer highlights the precision and consistency of the RF-sputtering process. This technique enabled the formation of a pinhole-free film within the targeted nanoscale range, in line with established protocols for high-quality thin-film deposition [65]. The combined morphological features, namely the dense, pinhole-free composite layer and the uniform Pt capping, are indicative of a robust architecture poised for effective charge transport and stability in a functional device.

*d*  *EDX-Mapping*

**Figure 4:**
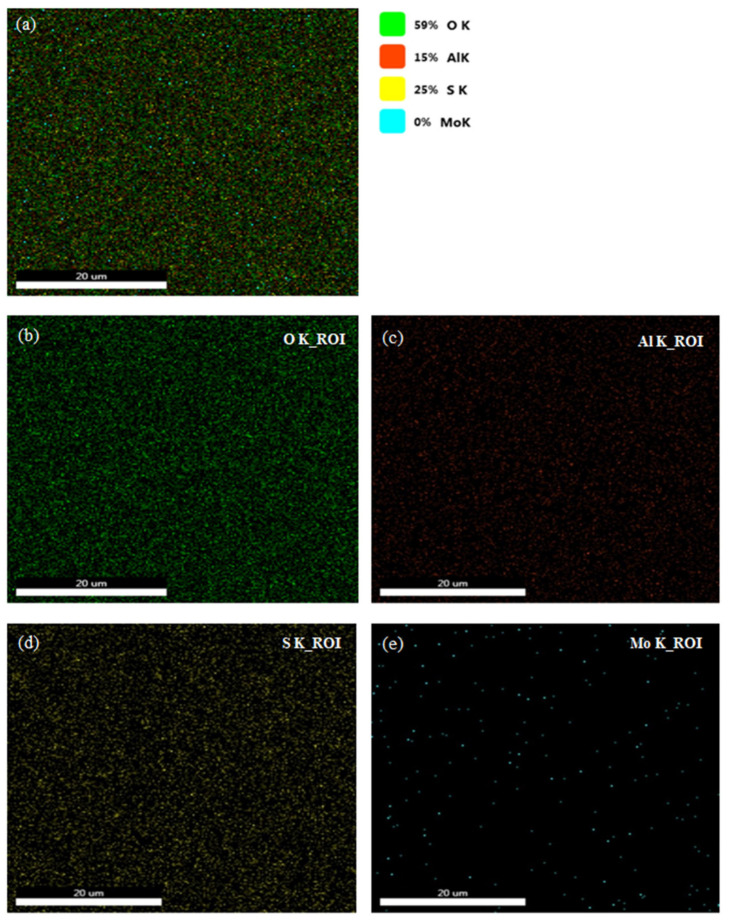
EDX mapping results of the MoS_2_–Al_2_O_3_ composite thin film: (**a**) elemental overlay illustrating the spatial distribution of constituent elements across the film surface; (**b**) Region Of Interest (ROI) mapping of oxygen (O); (**c**) ROI mapping of aluminum (Al); (**d**) ROI mapping of sulfur (S); and (**e**) ROI mapping of molybdenum (Mo), confirming the homogeneous dispersion of both oxide and chalcogenide phases.

In order to complete the verification of our proposed composite, Figure 4a illustrates the EDAX elemental mapping of the MoS_2_–Al_2_O_3_ composite thin film, revealing a spatially resolved distribution that reflects the structural and compositional characteristics of the hybrid system. The oxygen signal (O K-shell, 59%) shown in Figure 4b dominates the elemental profile, indicating the prevalence of an Al_2_O_3_ matrix. This elevated oxygen content may arise from surface hydroxylation or ambient adsorption, particularly in porous or nanostructured regions. The aluminum signal (Al K-shell, 15%) in Figure 4c confirms the incorporation of alumina. Its spatial correlation with oxygen suggests the formation of a continuous or partially enclosing Al_2_O_3_ phase. The elemental mapping further reveals that the thin film is dominated by an Al_2_O_3_ matrix, as evidenced by the strong and widespread oxygen (O K) signal together with the spatially correlated aluminum (Al K) signal, both characteristic of alumina. Additionally, the Sulphur elemental map shown in Figure 4d presents a well-defined and spatially coherent distribution, indicating that MoS_2_ is incorporated within the film either as surface-dispersed regions or embedded domains. In contrast, the Sulphur (S K) signal appears in localized regions that partially overlap with oxygen-rich areas, suggesting that MoS_2_ is present as discrete domains within the Al_2_O_3_ framework rather than as a continuous layer.

**Figure 5:**
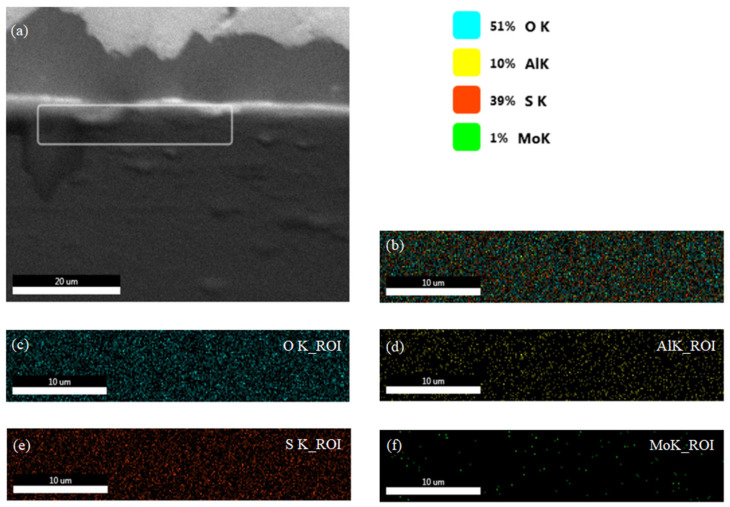
(**a**) DUT cross-section SEM image with a rectangle surrounding the cross-section under study and (**b**) elemental overlay illustrating the spatial distribution of constituent elements across the film cross-section. (**c**) Region Of Interest (ROI) mapping of oxygen, (**d**) ROI mapping of aluminum (Al), (**e**) ROI mapping of sulfur (S), and (**f**) ROI mapping of molybdenum (Mo).

Despite the clear Sulphur distribution, no molybdenum signal (Mo K-shell, 0%) is detected in the surface-sensitive EDAX map (Figure 4e). This absence can be attributed to the low Mo concentration relative to the detection limit, the presence of MoS_2_ as few-layer or monolayer domains, burial of Mo beneath the Al_2_O_3_ matrix, or limitations related to beam energy and detector sensitivity during acquisition. To confirm our expectation, complementary elemental mapping of the same region is provided in Figure 5. The composite EDAX map in Figure 5b illustrates the overall spatial distribution of O, Al, S, and Mo within the film. The oxygen elemental map in Figure 5c (O K-shell) shows a strong and continuous signal, confirming that Al_2_O_3_ forms the dominant matrix of the composite layer, while the aluminum map shown in Figure 5d (Al K-shell) closely follows the oxygen distribution, further verifying the formation of an alumina framework. The Sulphur map in Figure 5e (S K-shell) displays a well-defined and spatially coherent signal within localized regions, supporting the presence of MoS_2_ domains embedded within the Al_2_O_3_ matrix. Cross-sectional EDAX mapping performed at 30 kV, shown in Figure 5f, reveals measurable molybdenum intensity (1%, corresponding to 12.15 wt.%) together with Sulphur intensity (39%, corresponding to 0.02 wt.%), confirming that Mo is present beneath the surface and consistent with the layered MoS_2_ structure in which Mo atoms are sandwiched between Sulphur layers.

**Figure 6:**
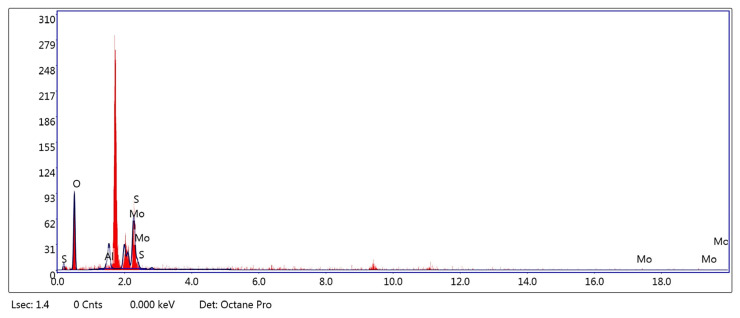
EDAX-Mapping spectrum at the cross-section.

Cross-sectional EDAX mapping combined with quantitative eZAF analysis provides clear evidence for the presence of molybdenum within the MoS_2_–Al_2_O_3_ composite film. The cross-sectional EDAX spectrum (Figure 6) shows distinct characteristic peaks of O, S, and Mo, confirming that Mo is embedded beneath the film surface rather than being surface-segregated. Furthermore, quantitative eZAF Smart Quant analysis (Table 2) reveals a measurable Mo content of ~12.15 wt.% (2.29 at%) in the cross-sectional region, while Sulphur appears at a negligible level (~0.02 wt.%). The statistically significant Mo signal, together with suppressed Sulphur, indicates that MoS_2_ is incorporated within the Al_2_O_3_ matrix and that Mo atoms are not directly exposed at the surface. The percentage was 99%, which is likely lower due to surface roughness or "shadowing" effects where the X-rays are blocked from reaching the detector by the topography of the MoS_2_–Al_2_O_3_ flakes.

**Figure 7:**
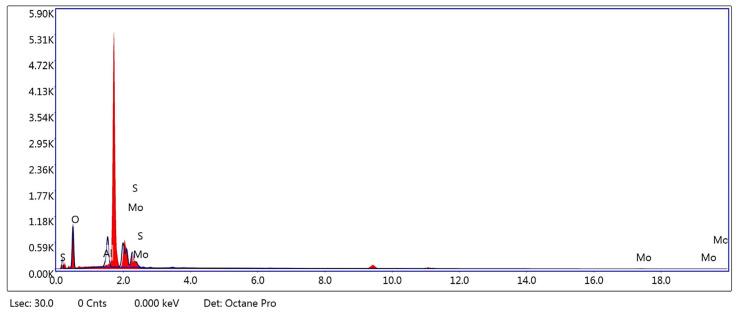
EDAX spectrum at a point at the cross-section interface.

To further verify this behavior, point EDAX analysis at the cross-sectional interface (Figure 7) is performed. The corresponding eZAF results (Table 3) show an increased Mo concentration of ~16.51 wt.% (3.25 at%) with Sulphur below the detection limit. The persistence of the Mo signal in the absence of Sulphur is fully consistent with the layered S–Mo–S crystallographic structure of MoS_2_, in which Mo atoms are sandwiched between Sulphur layers and can remain detectable in cross-sectional measurements. Cross-section EDAX-mapping total percentage was 101%, which is often higher due to backscattering or the sample being perfectly perpendicular to the beam, leading to a slight over-count in the detector’s processing algorithm. Overall, the combined spectral features (Figure 6 and Figure 7) and quantitative results (Table 2 and Table 3) provide conclusive evidence for the bulk incorporation of Mo atoms within the MoS_2_–Al_2_O_3_ composite film, consistent with the intrinsic layered structure of MoS_2_.

*e*  *XRD Analysis and Crystallinity Evaluation*

**Figure 8:**
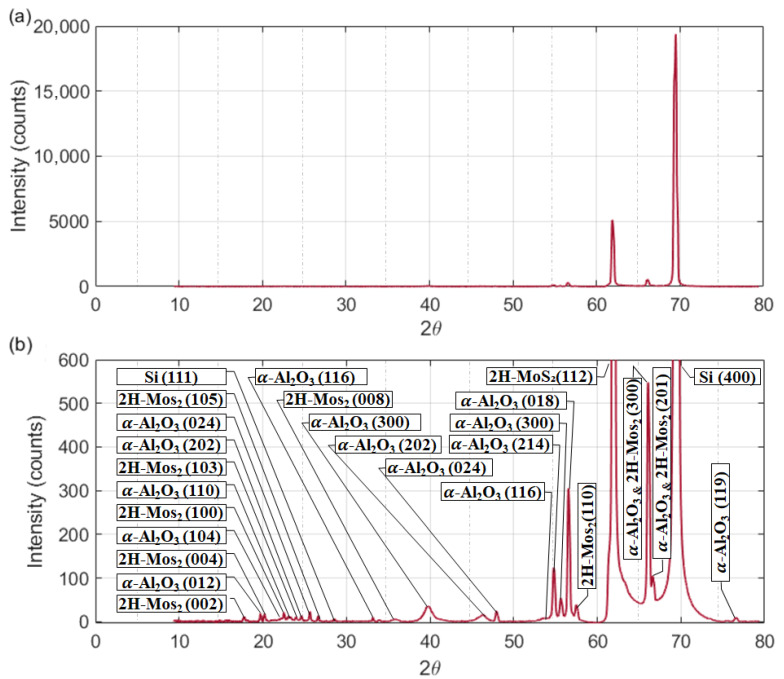
(**a**) XRD pattern of the MoS_2_–Al_2_O_3_ composite thin film deposited on p-type Si substrate, showing distinct reflections from both 2H-MoS_2_ and α-Al_2_O_3_ phases, alongside a substrate-derived Si (111) peak. The presence of sharp and well-resolved peaks confirms high crystallinity, phase purity, and structural integration across the layered chalcogenide, oxide matrix, and silicon interface. (**b**) Enlarged view of the characteristic peaks, highlighting their resolution and phase-specific assignments.

The presence of MoS_2_ is further corroborated by XRD analysis shown in Figure 8, which confirms the characteristic 2H-MoS_2_ phase. In this regard, the subsequent XRD results provide further insight into the phase composition and crystallographic features of the MoS_2_–Al_2_O_3_ composite thin film. Figure 8a presents the full-range XRD pattern of the MoS_2_–Al_2_O_3_ composite thin film, revealing a structurally coherent multiphase architecture characterized by distinct reflections attributable to both layered dichalcogenide and oxide constituents, while Figure 8b provides an enlarged view of the region containing multiple overlapping peaks that are difficult to resolve, extending from the low- to high-angle range. As shown in Figure 8b, Low-angle peaks at 17.8°, 20.2°, 23.12°, 24.04°, 26.7°, and 35.87° are assigned to the (002), (004), (100), (103), (105), and (008) planes of 2H-MoS_2_, respectively, confirming the preservation of its hexagonal layered structure and vertical stacking order. These reflections are consistent with the JCPDS card No. 37-1492 for 2H-MoS_2_ [66], confirming the presence of a well-crystallized layered dichalcogenide phase. These reflections are indicative of well-oriented MoS_2_ domains, essential for anisotropic charge transport and optoelectronic functionality. Concurrently, peaks at 19.7°, 22.5°, 23.38°, 24.73°, 25.69°, 33.17°, and 39.83° correspond to the (012), (104), (110), (202), (024), (116), and (300) planes of α-Al_2_O_3_, reflecting the presence of a thermally stable corundum phase which is consistent with JCPDS Card No. 10-0173 for α-Al_2_O_3_ (corundum phase) [67]. The peak at 28.6°, consistent with the (111) reflection of crystalline silicon (JCPDS 27-1402), confirms substrate influence [68], a common occurrence in thin film diffraction when the deposited layer is insufficiently thick to suppress underlying signals. The coexistence of MoS_2_ and Al_2_O_3_ reflections across the 2θ range, without significant peak broadening or shift, suggests minimal interfacial strain and effective phase separation. This structural integrity is further supported by EDX elemental mapping, which reveals a spatially uniform distribution of Mo, S, Al, and O across the film surface. Collectively, the diffraction and compositional data affirm the formation of a well-integrated composite thin film, suitable for applications requiring synergistic dielectric and semiconducting properties.

The high-angle region of the XRD pattern for the MoS_2_–Al_2_O_3_ composite thin film, shown in the enlarged scale in Figure 8b, reveals a series of sharp reflections indicative of well-crystallized oxide and chalcogenide phases. Peaks at 46.38° and 47.99° are consistent with the (202) and (024) planes of α-Al_2_O_3_ [67], confirming the persistence of the corundum structure at elevated diffraction angles. The reflection at 53.5° may correspond to the (116) plane of Al_2_O_3_ or a minor MoS_2_ contribution, depending on the degree of interfacial strain and stacking order. Peaks at 54.85°, 55.67°, and 56.53° are attributed to the (214), (300), and (018) planes of Al_2_O_3_, respectively [67], reinforcing the oxide’s phase purity and crystallographic coherence. The peak at 57.54° aligns with the (110) reflection of 2H-MoS_2_ [66], a key in-plane feature that confirms the retention of hexagonal symmetry within the chalcogenide domains. Finally, the reflections at 66.09° and 66.59° represent overlapping contributions from the (300) plane of Al_2_O_3_ and the (201) plane of MoS_2_, respectively [66,67], suggesting structural integration and possible interfacial ordering. The sharpness and separation of these peaks, with minimal broadening, indicate low defect density and effective phase separation, essential for the composite’s functional performance in optoelectronic and dielectric applications. The peak at 69.48°, absent from MoS_2_ and Al_2_O_3_ reference cards, matches the (400) reflection of crystalline silicon (JCPDS 27-1402) [68], confirming substrate influence. This is typical in thin film XRD when the film is semi-transparent to X-rays or insufficiently thick to suppress substrate signals.

*f*  *Surface Roughness*

**Figure 9:**
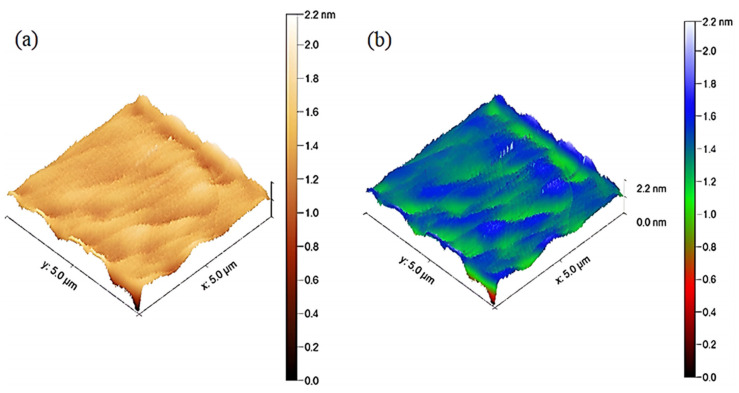
(**a**) 3D AFM topographic map of the MoS_2_–Al_2_O_3_ composite thin film acquired using a height-dependent brown-scale color gradient. (**b**) Corresponding AFM map of the same region visualized using a multicolor elevation scale for enhanced morphological contrast.

The spectroscopic (ATR, Raman) and microscopic (SEM, EDX-mapping) analyses, along with the crystallographic evidence from XRD, establish the chemical integrity and structural coherence of the MoS_2_–Al_2_O_3_ composite; the subsequent surface roughness evaluation (Figure 9) complements these findings by quantifying the nanoscale uniformity of the film morphology. Quantitative topographical assessment of the fabricated sample was performed over a 512 × 512 px matrix, revealing a statistically homogeneous surface profile with minimal morphological perturbations as shown in Figure 8a,b. The average elevation was determined to be 1.3498 nm, with a root-mean-square roughness (Sq) of 145.245 pm and a mean height deviation (Sa) of 111.937 pm, signifying nanoscale undulations confined within sub-nanometer limits. A negligible skewness value (Ssk = −0.0326), accompanied by moderate excess kurtosis (1.50996), denotes a quasi-Gaussian height distribution with slightly heavier tails—indicative of sporadic outliers but no systematic topographical asymmetry. A peak-to-valley excursion (Sz) of 2.17978 nm defines the vertical range, composed of discrete protrusions (Sp = 0.82998 nm) and depressions (Sv = 1.34980 nm). The absence of texture-induced area amplification is confirmed by the near-equivalence of projected and true surface areas (25 vs. 25.0026 µm^2^), underscoring the film’s near-planar morphology. In optoelectronic structures, these metrics collectively demonstrate a high degree of surface uniformity and smoothness essential for minimizing internal scattering effects and maximizing charge transport within the MoS_2_–Al_2_O_3_ composite layer. The 3D surface profiles visualized in Figure 9a,b further confirm the low-roughness and planar features of the composite thin film, displayed under two distinct height-mapping color schemes for enhanced interpretability.

### 3.2. Performance Analysis

The dynamic behavior of the fabricated MoS_2_–Al_2_O_3_/Si photodiode was systematically evaluated through a series of electrical and optoelectronic measurements. Key performance indicators, including photocurrent generation, temporal response, and spectral responsivity, were assessed under controlled biasing and optical excitation conditions. The analysis aimed to elucidate the role of the composite active layer in enhancing carrier transport, suppressing interfacial recombination, and extending the device’s detection capabilities. The following subsections present a detailed examination of the device’s operational characteristics, highlighting its potential for integration into low-power, broadband photodetection platforms suitable for LiDAR and environmental sensing applications.

*a*  *I–V output characteristics*

**Figure 10:**
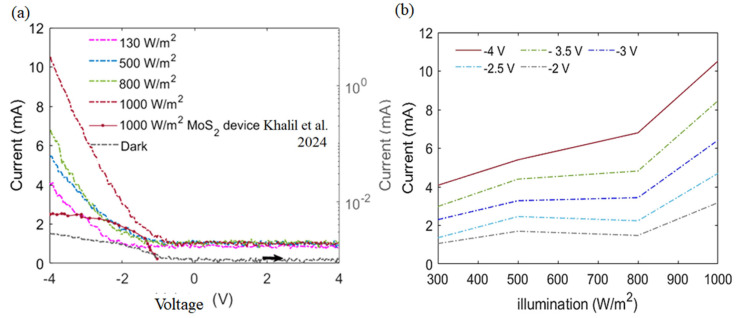
(**a**) IV–characteristics of MoS_2_–Al_2_O_3_ composite np-photodiode versus the previously fabricated MoS_2_ reported by Khalil et al. 2024 [23] and (**b**) the current versus the illumination at the different operating voltages.

In line with this comprehensive evaluation, the current–voltage (I–V) characteristics provide direct evidence of the device’s dynamic response, substantiating the role of the MoS_2_–Al_2_O_3_ composite layer in achieving efficient carrier transport and enhanced photodetection performance. The current–voltage (I–V) characteristics of the MoS_2_–Al_2_O_3_ composite PN photodiode are shown in Figure 10a in both dark and at different illumination intensities (130, 500, 800, and 1000 W/m^2^). The right axis is drawn in logarithmic scale with respect to the small dark current of 5 × 10^−2^ mA for the proposed device. The gadget clearly exhibits photovoltaic behavior, with the photocurrent rising sharply as the light intensity increases. Interestingly, the device shows a high light-to-dark current ratio with a maximum photocurrent of roughly 10 mA under 1000 W/m^2^ of illumination at a reverse bias of −4 V and a far smaller dark current (<1 mA). This behavior confirms the efficient photogeneration and collection of carriers within the composite layer. Figure 10a also shows the IV biased characteristics from 1 to −4 V at the 1000 W/m^2^ reported by Khalil et al. (2024) [23]. The superior performance of the MoS_2_–Al_2_O_3_ composite arises from enhanced dielectric modulation and reduced recombination losses. In contrast, the reference device’s higher dark current and lower photocurrent under identical conditions likely result from the lack of composite-induced passivation and interfacial tuning present in the RF-sputtered DUT. Figure 10b illustrates the dependence of current on illumination intensity at various applied reverse bias voltages (−2 to −4 V). A nearly linear increase in photocurrent is observed with increasing irradiance, particularly under higher bias voltages, which enhances the electric field across the junction and improves carrier separation [69]. This linear trend reflects the photoconductive nature of the device and its sensitivity to light intensity.

Our results show that the device measurements exhibit excellent linearity with increasing the operating voltage, especially up to the illuminance of 800 W/m^2^. At such illuminance, the current reaches 1.48 mA at a biasing voltage of 2 V. The current rises to 2.24 mA, 3.44 mA, and 4.82 mA, respectively, when the voltage is increased to 2.5 V, 3 V, and 3.5 V. At 4 V, the current reaches 6.8 mA, demonstrating improvements of nearly 95% and 350% compared to the 3 V and 2 V operating voltages. These results confirm that the MoS_2_–Al_2_O_3_ composite layer, formed as a unified hybrid structure, is a promising candidate for high-performance photodetection due to its enhanced charge transport and robust light absorption characteristics. One can understand that the previously fabricated photodiode had a lower photocurrent (I_L_) of 2.5 mA at 4 V. Compared to the previously reported device shown in Figure 10a, the photocurrent of the new composite structure is improved by 300% relative to the previously reported structure.

*b*  *Relative Spectral responsivity*

**Figure 11:**
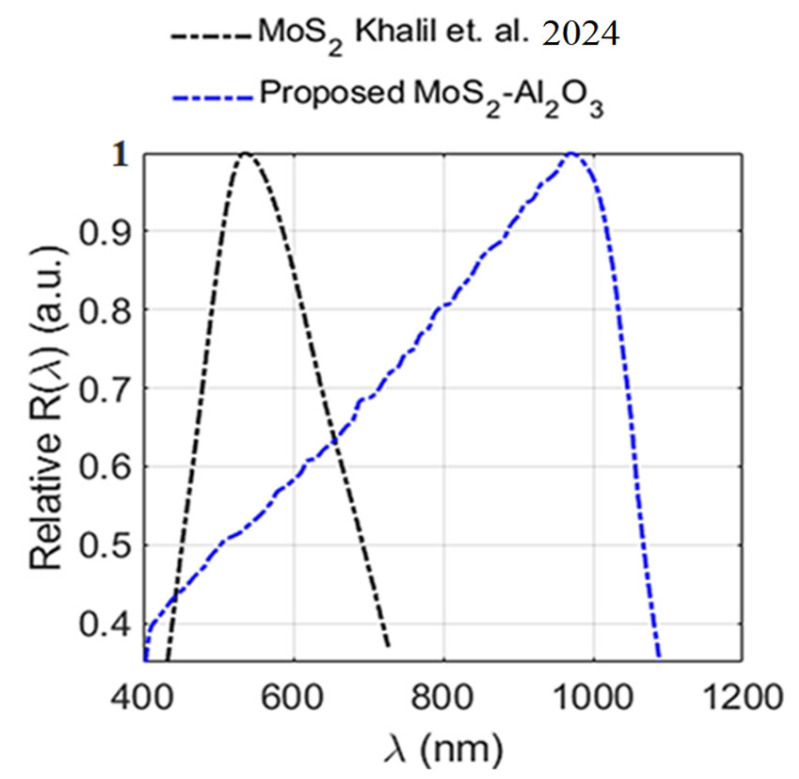
Relative spectral responsivity for fabricated MoS_2_–Al_2_O_3_ composite PN photodiode and baseline MoS_2_ PN device reported by Khalil et al. 2024 [23], highlighting extended sensitivity beyond 970 nm.

While the I–V characteristics established the efficient carrier transport and strong photocurrent generation under varying illumination, the subsequent spectral responsivity measurement (Figure 11) extends this analysis by revealing the wavelength-dependent sensitivity of the MoS_2_–Al_2_O_3_ composite photodiode in comparison with previously reported MoS_2_-based detectors. It is worth noting that accurate determination of R requires that the DUT and the calibrated reference detector be illuminated under identical optical and geometric conditions. In our measurement setup, the photodiode was mounted on a compact probe station to enable controlled biasing during spectral scans, which made identical optical alignment with the reference detector impractical. Figure 11 illustrates the relative spectral responsivity of the fabricated MoS_2_–Al_2_O_3_ composite PN photodiode (DUT) across a wavelength range of 400 nm to 1100 nm. The responsivity of the previously published MoS_2_ photodetector relative to the recommended structure is also determined. It is worth noting that the peak sensitivity wavelength of MoS_2_-based detectors typically lies around 700 nm [70]. In contrast, the composite device’s measured responsivity increases with wavelength, peaking near 970 nm before gradually declining at longer wavelengths. A maximum output responsivity is recorded at 970 nm, indicating peak spectral response in the near-infrared region. Comparatively, the MoS_2_ thin-film photodiode fabricated by Khalil et al. (2024) [23] exhibited a narrower spectral response, with a peak responsivity at 535 nm and negligible extension into the near-infrared. This limitation is attributed to the reference device’s lack of dielectric-induced modulation and defect passivation, which are present in the MoS_2_–Al_2_O_3_ composite device and play a critical role in enhancing its efficiency. As a result, the proposed composite device demonstrates extended spectral sensitivity and enhanced performance at longer wavelengths. These findings underscore the redshifted photoresponse and broader spectral coverage enabled by the composite architecture.

In the unpassivated MoS_2_ photodiode reported by Khalil et al. [23], the responsivity peaks in the visible region (~535 nm), dominated by direct optical transitions in MoS_2_. However, the relatively thick MoS_2_ layer exceeds the minority-carrier diffusion length, leading to substantial recombination of carriers generated deep within the film before collection. In contrast, the proposed MoS_2_–Al_2_O_3_ composite exhibits suppressed visible response and a pronounced infrared peak centered near 970 nm. Similar infrared enhancement has been attributed to carrier generation deep within the Si substrate, away from interface defects, enabling efficient collection [71]. Accordingly, the reduced visible responsivity in the composite is governed by surface recombination rather than limited optical absorption. The incorporation of Al_2_O_3_ plays a central role in modifying the electronic and optical response. Dielectric screening and defect passivation suppress nonradiative recombination, extend carrier diffusion length, and improve charge extraction [20,72,73]. Concurrently, Al_2_O_3_ induces mild tensile strain in MoS_2_, introducing shallow sub-gap states that broaden the absorption spectrum without significantly reducing carrier lifetime [74]. These effects collectively account for the observed redshifted and broadened spectral responsivity shown in Figure 11.

The novelty of this work lies in the use of an MoS_2_–Al_2_O_3_ composite interface that enhances charge separation and suppresses interface trap-assisted recombination. This behavior has not been reported in earlier MoS_2_ devices [4,15,20,59,72,75,76] that use dielectric layers only as surface capping or simple passivation. In our design, the Al_2_O_3_ is embedded inside the MoS_2_ matrix instead of being placed as a top layer. This produces a hybrid internal barrier that improves vertical carrier transport. It also increases responsivity and reduces dark current. The composite architecture introduces a new paradigm in which dielectric incorporation fundamentally alters the electronic behavior of two-dimensional semiconductors. It further provides a practical route for achieving higher detectivity and lower power consumption in future optoelectronic devices. Structurally, the co-sputtered composite forms a homogeneous network in which Al_2_O_3_ is uniformly embedded within the MoS_2_ matrix (Figure 11). Deposited on p-type Si, this architecture differs fundamentally from a conventional heterojunction by enabling spatially distributed carrier generation and transport within a single blended layer. MoS_2_ remains the primary photoactive material, while the surrounding Al_2_O_3_ matrix regulates surface states and facilitates carrier transport. The nanoscale dispersion of MoS_2_ domains further suppresses recombination, particularly under near-infrared illumination. Thickness-dependent bandgap modulation also contributes to the infrared response. MoS_2_ layers thicker than ~20 nm exhibit bulk-like behavior with reduced bandgap energy [77]. Extrapolating this trend, the ~70 nm MoS_2_ thickness in the present composite corresponds to an effective bandgap near ~1.05 eV, consistent with the measured photoresponsivity spectrum (Figure 10). This observation aligns with reports of bandgap narrowing in multilayer and polycrystalline MoS_2_ [78]. Analogous redshifted absorption has been reported in oxide-coupled MoS_2_ heterostructures, where band alignment and interfacial coupling enhance charge transfer and suppress recombination [79]. The enhanced electronic performance, along with the observed infrared peak and redshift in the MoS_2_–Al_2_O_3_ composite, can be attributed to a combination of optical and electronic mechanisms, as described below

I—Electronic effects (most likely dominant).

From an electronic perspective, dielectric screening is the dominant mechanism. Pristine MoS_2_ exhibits weak intrinsic screening and large exciton binding energies, limiting free-carrier generation [80,81]. Embedding MoS_2_ within a high-κ dielectric environment such as Al_2_O_3_ weakens electron–hole Coulomb interactions, reduces exciton binding energy, and enhances carrier separation [82]. Additionally, the negative fixed charges in Al_2_O_3_ modify the MoS_2_ Fermi level, increase electron density, and enhance the built-in potential at the MoS_2_/Si junction, improving carrier collection efficiency [72,73].

**Figure 12:**
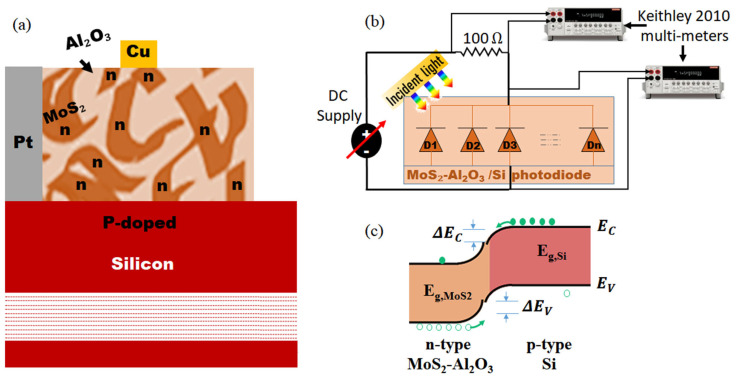
(**a**) 2D side view schematic diagram of the proposed photodetector, (**b**) the corresponding localized diode model representation of the MoS_2_AlO_3_ composite, and (**c**) energy band diagram showing how Al_2_O_3_ modulates the built-in voltage and strains the MoS_2_ for a bandgap close to the Si substrate.

As shown in (Figure 12a), at the MoS_2_–Al_2_O_3_/Si interface, Al_2_O_3_ acts as both an insulating and passivating medium, suppressing trap-assisted recombination and stabilizing interfacial chemistry. These effects are analogous to defect passivation observed in oxide-embedded semiconductor systems, where dielectric encapsulation limits grain growth and improves recombination dynamics [83,84]. Consistent with this interpretation, co-sputtered MoS_2_–Al_2_O_3_ films have been shown to exhibit enhanced absorption and modified optical constants without disrupting MoS_2_ crystallinity [20].

As presented in (Figure 12c) Strain-induced bandgap modulation provides an additional contribution. Increasing Al_2_O_3_ thickness enhances dielectric and strain coupling, progressively reducing the MoS_2_ bandgap toward ~1.0 eV at intermediate thicknesses [74]. In the present composite, local variations in strain and dielectric environment create a distribution of bandgaps and built-in potentials, which can be modeled as parallel-connected photodiodes (Figure 12b). This distributed response collectively produces the observed broadband and infrared-enhanced photoresponse.

Overall, the broadened spectral response and enhanced photocurrent of the sputtered n-MoS_2_–Al_2_O_3_/p-Si photodiode arise from synergistic dielectric screening, defect passivation, and strain-induced band modulation within a single composite layer. These mechanisms suppress recombination, enhance carrier transport, and extend sensitivity into the near-infrared, distinguishing the composite architecture from conventional MoS_2_ thin-film photodiodes.

II—Optical-photonic effects.

The nanoscale dispersion of Al_2_O_3_ also reduces surface reflectance, allowing more light to penetrate into MoS_2_-rich regions [85]. Furthermore, optimized Al_2_O_3_ coatings reduce optical reflectance and improve light harvesting in MoS_2_-based devices [86]. Further, the observed redshift and extended absorption of the MoS_2_–Al_2_O_3_ composite up to approximately 1100 nm can be attributed to an increased optical path length and enhanced photon confinement within the heterostructure. The intermixing between MoS_2_ and Al_2_O_3_ promotes internal light scattering and increases the effective absorption thickness, enabling more efficient photon absorption at longer wavelengths [87]. Furthermore, partial photonic interference and resonant scattering within the dielectric–semiconductor blend may contribute to additional enhancement and red-shifting of the absorption spectrum [88]. The extended absorption tail approaching 1100 nm may also result from carrier collection within the Si substrate, where the presence of Al_2_O_3_ improves the MoS_2_/Si interfacial light coupling and overall optical integration efficiency.

*c*  *Photo-transient response time*

**Figure 13:**
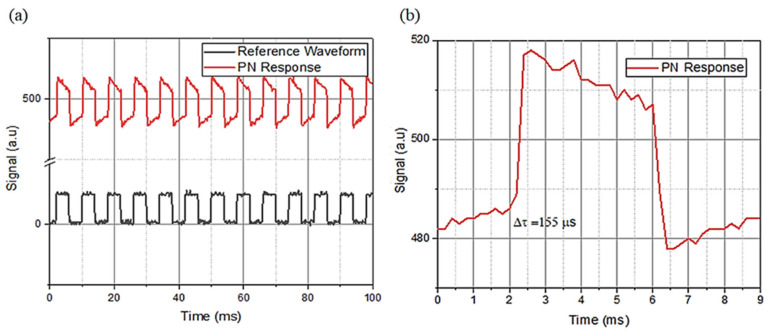
(**a**) PN sample response time, (**b**) inset of response time of PN sample showing about 155 μs.

The temporal photoresponse of the MoS_2_–Al_2_O_3_ composite-based PN photodiode was evaluated under modulated illumination to assess its dynamic behavior, as shown in Figure 12. The periodic on/off illumination produces a stable and reproducible output signal, where the p–n response (red curve) closely follows the reference waveform (black), indicating reliable switching behavior and fast carrier dynamics (Figure 13a). A magnified view of a single cycle (Figure 13b) reveals a sharp rise in the photocurrent with a response time of approximately 155 µs. This fast response is consistent with efficient carrier generation and extraction within the integrated MoS_2_–Al_2_O_3_ composite layer. Notably, the measured response time is more than an order of magnitude faster than that reported for pristine MoS_2_-based devices [23]. The improved temporal response can be attributed to the combined effects of dielectric screening and defect passivation introduced by Al_2_O_3_, which enhance the internal electric field and suppress trap-assisted recombination, thereby facilitating faster carrier extraction [72,73]. In addition, strain-related band modulation and dielectric coupling may further improve carrier transport, contributing to the observed acceleration in photoresponse [74].

The performance comparison has expanded beyond our previous analysis to provide a clearer context for the developed device. Table 4 summarizes recent MoS_2_-based photodiodes with comparable architectures reported in the literature. This table outlines the key performance indicators, including device structure type, fabrication method, peak detection wavelength, spectral responsivity trend, and response time. Through this comparison, the relative position of our MoS_2_–Al_2_O_3_ composite photodiode is established, demonstrating its competitive performance in terms of responsivity, response speed, and broadband NIR sensitivity with respect to state-of-the-art devices.

### 3.3. Application Perspective: Environmental LiDAR System

Building on the demonstrated microsecond-scale temporal photoresponse, the application perspective highlights how the MoS_2_–Al_2_O_3_ photodiode’s dynamic behavior directly translates into practical advantages for environmental LiDAR and optical-sensing systems. The demonstrated microsecond-scale response of the MoS_2_–Al_2_O_3_ photodiode provides clear advantages for near-infrared (NIR) LiDAR and optical-sensing systems. Modern eye-safe LiDAR modules operating in the 850–1000 nm window commonly rely on detectors with responsivities on the order of a few-tenths of an ampere per watt and sub-microsecond to microsecond response times; representative 850 nm photodiodes and APDs report responsivities ≈0.4–0.5 A/W with very low dark currents and very fast rise times [71]. To clarify the performance relevance of the MoS_2_–Al_2_O_3_ photodiode in NIR LiDAR and sensing applications, we discuss its suitability within the framework of current system requirements.

Practical LiDAR implementations and instrumentation reports further emphasize the importance of fast detector response and low noise for achieving a high signal-to-noise ratio in short- and mid-range detection [25]. The demonstrated microsecond-scale response of the MoS_2_–Al_2_O_3_ photodiode therefore supports accurate time-of-flight (ToF) ranging with centimeter-scale resolution at high pulse rates, consistent with recent demonstrations of MHz-class LiDAR acquisition and ToF architectures [26]. Modern eye-safe NIR LiDAR receivers designed for environmental monitoring, forestry canopy profiling, and atmospheric mapping typically operate within the 850–1000 nm window, emphasizing signal stability, high sensitivity, and low dark current rather than nanosecond-scale response times [24,25,26]. Commercial slow-scan systems (e.g., RIEGL VZ-series and Mica Sense Altum-PT) employ photodetectors with temporal responses in the tens to hundreds of microseconds optimized for long-range, low-repetition-rate scanning and extended integration [27,28].

The 150 µs rise time measured for our MoS_2_–Al_2_O_3_ photodiode corresponds to a bandwidth of approximately 1 kHz, aligning well with these slow-scan LiDAR regimes. Although this response precludes centimeter-scale pulsed time-of-flight (ToF) ranging where the intrinsic depth resolution (Δd ≈ cΔt/2) equals about 22.5 km, it remains fully compatible with phase-based or integrative LiDAR architectures, in which spatial resolution depends on beam-scanning step size and averaging rather than detector timing. The device exhibits a current density of 10 mA cm^−2^ at 970 nm and a dark current of 5 × 10^−2^ mA at 4 V bias, ensuring a high signal-to-noise ratio and minimal baseline drift during long-integration measurements is key for canopy-height retrieval and topographic profiling under low-photon conditions. Therefore, while the photodiode’s temporal response limits its use in GHz-class automotive LiDAR, its strong NIR sensitivity, low dark current, and CMOS-compatible, low-cost fabrication make it ideally suited for environmental and slow-scan LiDAR applications operating at eye-safe wavelengths and moderate repetition rates. Future optimization will aim to further reduce dark current and noise-equivalent power (NEP) and enhance temporal response through interface passivation and geometry refinement, extending its applicability toward faster-scanning and higher-precision remote-sensing architectures.

## 4. Conclusions

The MoS_2_–Al_2_O_3_ composite-based photodiode exhibits enhanced broadband and NIR photodetection performance, as evidenced by increased photocurrent, extended spectral responsivity, and faster temporal response compared with the reference device. The low dark current and high illumination current indicate effective defect passivation enabled by the Al_2_O_3_ component, while the sustained responsivity up to 1100 nm highlights the composite’s improved NIR sensitivity. A response time of 155 µs confirms accelerated carrier dynamics, consistent with reduced trap-assisted recombination and improved carrier transport within the composite layer. Overall, the experimental results demonstrate that integrating Al_2_O_3_ into the MoS_2_ matrix is an effective strategy for improving photodiode performance, positioning the MoS_2_–Al_2_O_3_ composite as a promising low-cost platform for broadband NIR detection in eye-safe LiDAR and environmental sensing applications.

## Figures and Tables

**Table 1:** Summary of key vibrational modes.

Wavenumber (cm^−1^)	Intensity	Mode/Material	Significance
613.32	~95	Al–O stretch	Crystalline α-Al_2_O_3_ formation (47, 48)
820.13	~95	MoS_2_ E_2_g	In-plane vibrational mode (50, 51)
858.44	~95	MoS_2_ A_1_g	Out-of-plane vibrational mode (47, 48)
1020.15	~94.5	Interlayer/defect modes	Possible defect or strain-related vibrations (50, 51)
1110.81	~94.2	Overtone/surface modes	Surface interactions (55)
1455.19	~93	Si–O stretch	Silicon substrate oxidation (52)
1737.42	~93	Hydroxyl/water	Surface adsorbates (53, 54)
2351.3	~93	MoS_2_ overtone/combination modes	Multi-phonon vibrational modes (55)
2856.8–2923.6	~95	Alumina overtones	Lattice vibrations of α-Al_2_O_3_ (49)

**Table 2:** eZAF Smart Quant Results for the spectrum at the cross-section.

Element	Weight %	Atomic %	Net Int.	Error %	Kratio	Z	A	F
**O K**	84.90	95.74	752.60	10.82	0.3227	1.0311	0.3687	1.0000
**AlK**	2.93	1.96	69.00	13.76	0.0106	0.9306	0.3875	1.0014
**S K**	0.02	0.01	0.70	99.99	0.0001	0.9371	0.7635	1.0031
**MoK**	12.15	2.29	9.40	81.63	0.1012	0.6854	1.0137	1.1986

**Table 3:** eZAF Smart Quant Results at a point at the cross-section interface.

Element	Weight %	Atomic %	Net Int.	Error %	Kratio	Z	A	F
**O K**	79.70	94.10	368.03	8.79	0.2472	1.0428	0.2974	1.0000
**AlK**	3.79	2.65	58.16	8.04	0.0140	0.9423	0.3897	1.0059
**MoL**	16.51	3.25	229.23	3.14	0.1357	0.7637	1.0775	0.9992
**S K**	0.00	0.00	0.03	99.99	0.0000	0.9494	0.7526	1.0123

**Table 4:** Performance of MoS_2_–Al_2_O_3_ Photodiodes compared to Other Previous Work.

Study	Structure Type	Fabrication Method	Peak Wavelength	Response Time	Current
This work	MoS_2_–Al_2_O_3_ Composite	RF Sputtering	970 nm	155 µs	20.8 mA/cm^2^ *
Xiao, Peng, et al. (2018) [89]	3D heterojunction: RGO–MoS_2_ layered on pyramid-textured Si	Solution processing of RGO and MoS_2_ nanosheets on pyramid Si	850 nm	Slower: ~1.2 ms dynamics	-
Lou, Zhenhua, et al. (2017) [90]	MoS_2_/Si heterojunction	Chemical vapor deposition (CVD) for MoS_2_ + standard Si processing	~820 nm	-	2 mA/cm^2^
Ahn, Jongtae, et al. (2020) [91]	WSe_2_/MoS_2_ heterojunction photodiode	Mechanical exfoliation and dry transfer of 2D layers	~950 nm	-	-
Liu et al. (2024) [92]	Nitrogen plasma doping and lithography	Localized doping and channel preservation	1450	~200 µs	-
Li et al., 2024 [4]	MSM MoS_2_ with Al_2_O_3_/HfO_2_ passivation	CVD and ALD	850	210 µs	-
Wang et al., 2023 [93]	ReS_2_/MoS_2_ Heterojunction	Layered stacking	635 nm	-	-
P. Desai et al. [71]	MoS_2_/p-Si heterojunction	Molybdenum oxide (MoO_3_) (approximately 0.1 g) was deposited on the surface of p-type Si using the thermal evaporation technique MoS_2_ layer was synthesized by the sulfurization process at a higher temperature and an Ar + H_2_ inert atmosphere.	860 nm	-	22.5 mA/cm^2^

* Based on our effective area A = 0.6 × 0.8 = 0.48 cm^2^.

## Data Availability

Raw data were generated at the [Cairo University, NIS, and Zewail City of Science, Technology and Innovation] large-scale facility. Derived data supporting the findings of this study are available from the corresponding author upon request.

## Supplementary Materials

The following supporting information can be downloaded at: https://www.mdpi.com/article/10.3390/s26020542/s1, Figure S1: (a) side view of the PN photodiode deposited layers and (b) SEM of the top surface of MoS_2_–Al_2_O_3_ composite TF; Figure S2: (a) Pt thickness and (b) MoS_2_–Al_2_O_3_ composite layer thickness; Table S1: ATR data; Table S2: XRD data.
