# Supplementary material for: Engineered PN MoS2–Al2O3-Based Photodiode Device for High-Performance NIR LiDAR and Sensing Applications
Source: Sensors (Basel). 2026 Jan 13;26(2):542. doi: 10.3390/s26020542 (PMC12845837; doi:10.3390/s26020542)
Supplement: Supplementary file 1 [file sensors-26-00542-s001.zip › sensors-4027805-supplementary.pdf]

Supplementary file

S1. Side view, thickness and top view SEM Results of the PN photodiode

(a)

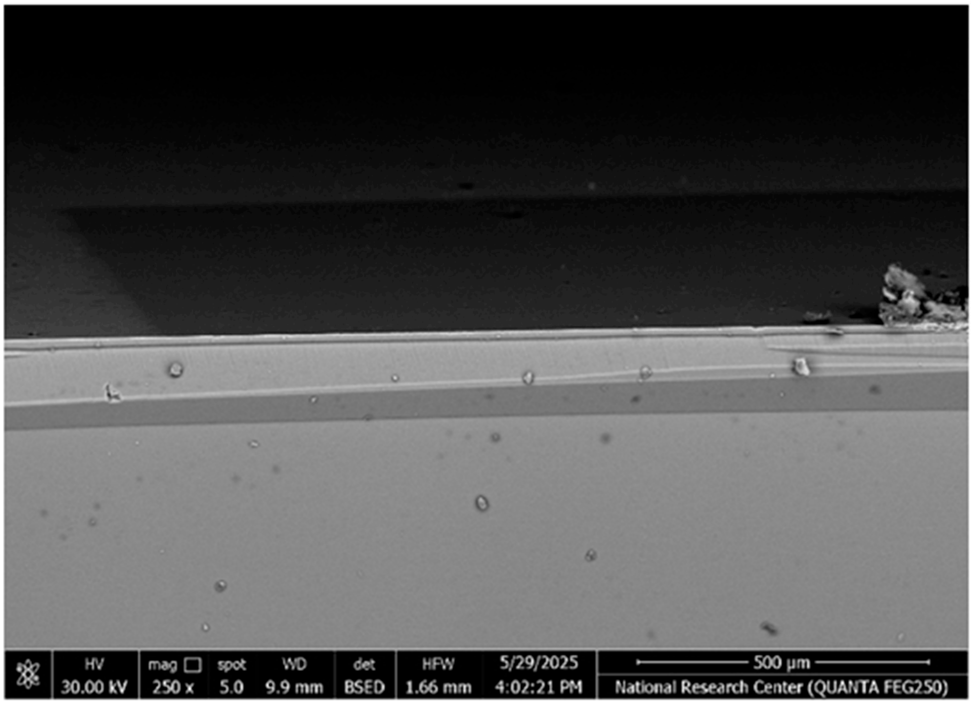

(b)

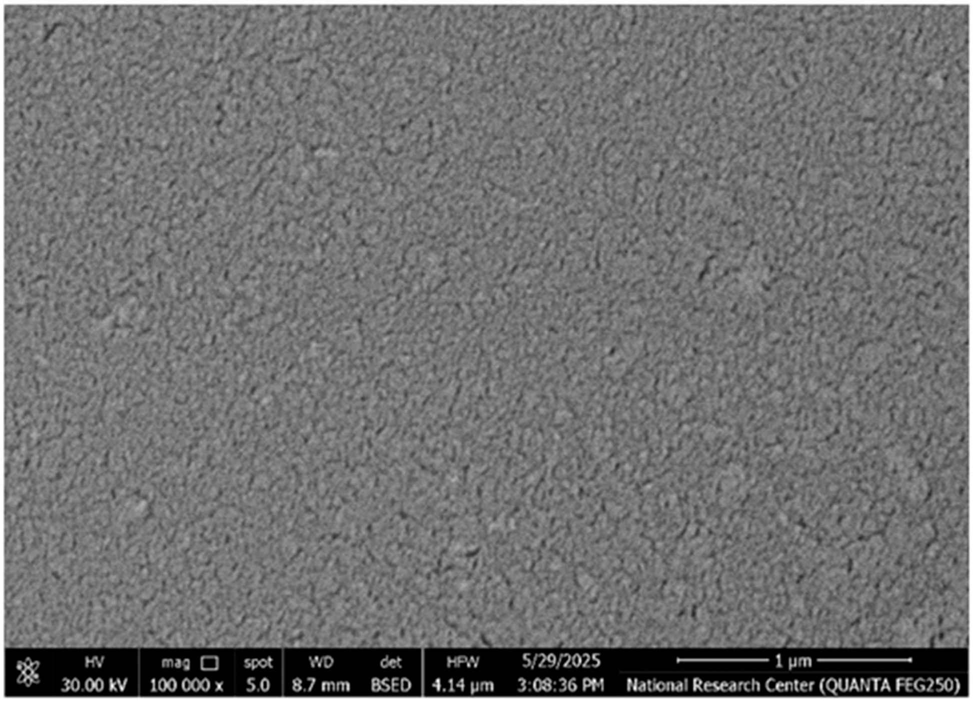

***Fig.* S1** *(a) side view of the PN photodiode deposited layers and (b) SEM of the top surface of* MoS_2–Al_2O_3 *composite TF*

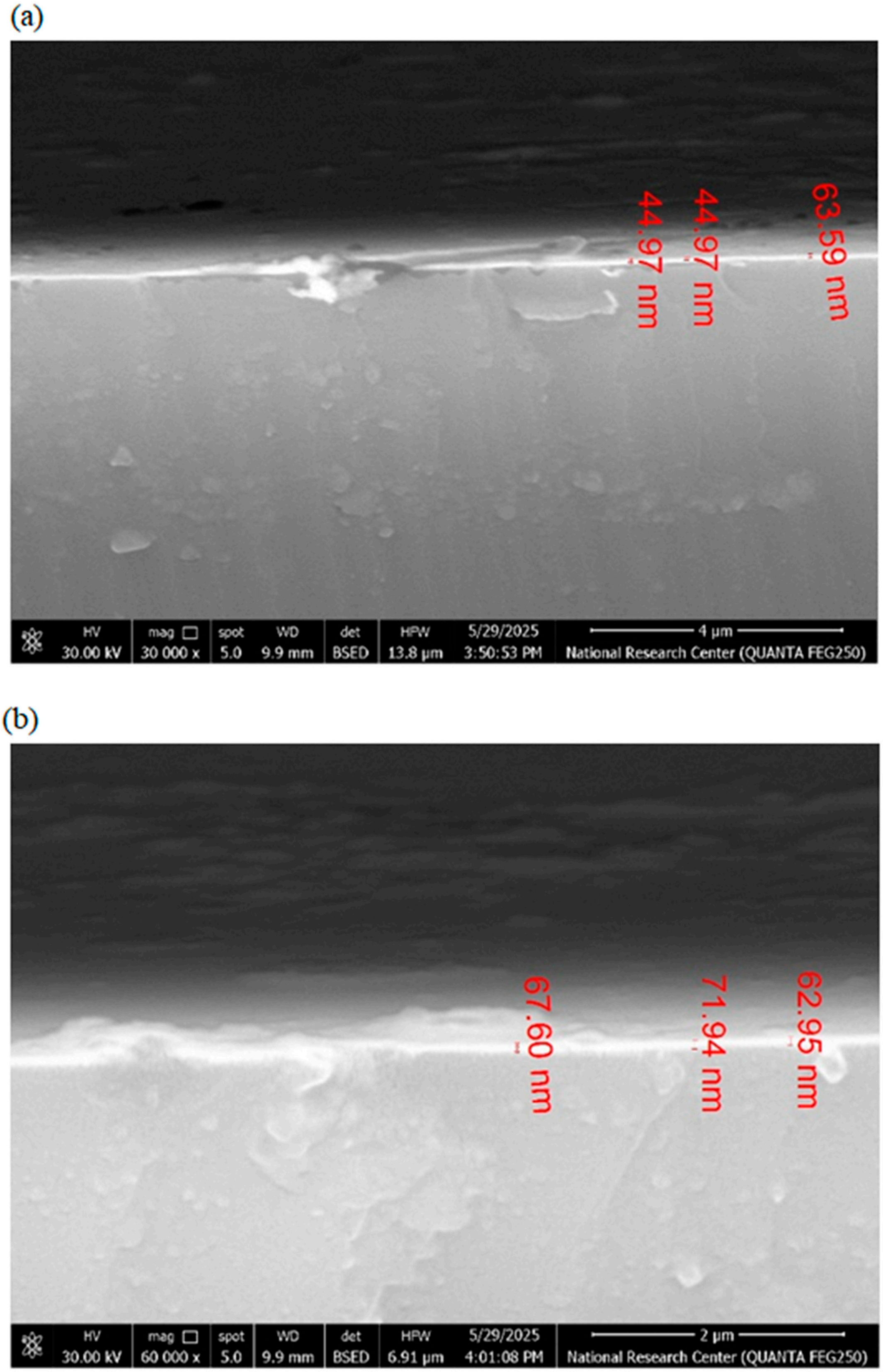

Fig. S2 *(a) Pt thickness and (b)* MoS_2*–*Al_2O_3 *composite layer thickness.*

S2. Characterization data (XRD, ATR, Raman chart)

Table S1. ATR data

Wavenumbers	Transmittance
5.79E+02	0.00E+00
5.82E+02	0.00E+00
5.86E+02	0.00E+00
5.90E+02	0.00E+00
5.94E+02	0.00E+00
5.98E+02	0.00E+00
6.02E+02	8.93E+01
6.06E+02	8.89E+01
6.09E+02	8.83E+01
6.13E+02	8.80E+01
6.17E+02	8.83E+01
6.21E+02	8.93E+01
6.25E+02	9.07E+01
6.29E+02	9.22E+01
6.33E+02	9.34E+01
6.36E+02	9.44E+01
6.40E+02	9.50E+01
6.44E+02	9.55E+01
6.48E+02	9.59E+01
6.52E+02	9.62E+01
6.56E+02	9.65E+01
6.60E+02	9.66E+01
6.63E+02	9.67E+01
6.67E+02	9.67E+01
6.71E+02	9.68E+01
6.75E+02	9.69E+01
6.79E+02	9.71E+01
6.83E+02	9.73E+01
6.87E+02	9.76E+01
6.90E+02	9.78E+01
6.94E+02	9.80E+01
6.98E+02	9.82E+01
7.02E+02	9.83E+01
7.06E+02	9.85E+01
7.10E+02	9.88E+01
7.14E+02	9.91E+01
7.17E+02	9.94E+01
7.21E+02	9.96E+01
7.25E+02	9.96E+01
7.29E+02	9.96E+01
7.33E+02	9.96E+01
7.37E+02	9.95E+01
7.41E+02	9.95E+01
7.44E+02	9.95E+01
7.48E+02	9.95E+01
7.52E+02	9.95E+01
7.56E+02	9.95E+01
7.60E+02	9.95E+01
7.64E+02	9.95E+01
7.68E+02	9.96E+01
7.71E+02	9.98E+01
7.75E+02	9.99E+01
7.79E+02	1.00E+02
7.83E+02	1.00E+02
7.87E+02	9.98E+01
7.91E+02	9.95E+01
7.95E+02	9.91E+01
7.98E+02	9.87E+01
8.02E+02	9.84E+01
8.06E+02	9.82E+01
8.10E+02	9.80E+01
8.14E+02	9.80E+01
8.18E+02	9.79E+01
8.22E+02	9.79E+01
8.25E+02	9.80E+01
8.29E+02	9.80E+01
8.33E+02	9.82E+01
8.37E+02	9.83E+01
8.41E+02	9.84E+01
8.45E+02	9.84E+01
8.49E+02	9.84E+01
8.52E+02	9.83E+01

8.56E+02	9.82E+01
8.60E+02	9.82E+01
8.64E+02	9.83E+01
8.68E+02	9.85E+01
8.72E+02	9.86E+01
8.76E+02	9.88E+01
8.79E+02	9.87E+01
8.83E+02	9.84E+01
8.87E+02	9.80E+01
8.91E+02	9.75E+01
8.95E+02	9.69E+01
8.99E+02	9.64E+01
9.03E+02	9.60E+01
9.06E+02	9.56E+01
9.10E+02	9.53E+01
9.14E+02	9.51E+01
9.18E+02	9.49E+01
9.22E+02	9.47E+01
9.26E+02	9.46E+01
9.30E+02	9.46E+01
9.33E+02	9.47E+01
9.37E+02	9.47E+01
9.41E+02	9.48E+01
9.45E+02	9.48E+01
9.49E+02	9.48E+01
9.53E+02	9.47E+01
9.57E+02	9.45E+01
9.60E+02	9.43E+01
9.64E+02	9.40E+01
9.68E+02	9.37E+01
9.72E+02	9.33E+01
9.76E+02	9.29E+01
9.80E+02	9.24E+01
9.84E+02	9.20E+01
9.87E+02	9.16E+01
9.91E+02	9.11E+01
9.95E+02	9.05E+01
9.99E+02	8.98E+01
1.00E+03	8.90E+01
1.01E+03	8.82E+01
1.01E+03	8.74E+01
1.01E+03	8.68E+01
1.02E+03	8.64E+01
1.02E+03	8.64E+01
1.03E+03	8.68E+01
1.03E+03	8.75E+01
1.03E+03	8.85E+01
1.04E+03	8.97E+01
1.04E+03	9.07E+01
1.05E+03	9.15E+01
1.05E+03	9.20E+01
1.05E+03	9.20E+01
1.06E+03	9.15E+01
1.06E+03	9.07E+01
1.06E+03	8.96E+01
1.07E+03	8.84E+01
1.07E+03	8.74E+01
1.08E+03	8.66E+01
1.08E+03	8.60E+01
1.08E+03	8.54E+01
1.09E+03	8.49E+01
1.09E+03	8.42E+01
1.10E+03	8.35E+01
1.10E+03	8.27E+01
1.10E+03	8.21E+01
1.11E+03	8.16E+01
1.11E+03	8.15E+01
1.11E+03	8.16E+01
1.12E+03	8.20E+01
1.12E+03	8.25E+01
1.13E+03	8.30E+01
1.13E+03	8.35E+01
1.13E+03	8.39E+01
1.14E+03	8.42E+01
1.14E+03	8.44E+01
1.15E+03	8.44E+01
1.15E+03	8.44E+01

1.15E+03	8.43E+01
1.16E+03	8.42E+01
1.16E+03	8.42E+01
1.16E+03	8.42E+01
1.17E+03	8.45E+01
1.17E+03	8.48E+01
1.18E+03	8.54E+01
1.18E+03	8.60E+01
1.18E+03	8.66E+01
1.19E+03	8.71E+01
1.19E+03	8.76E+01
1.20E+03	8.80E+01
1.20E+03	8.84E+01
1.20E+03	8.86E+01
1.21E+03	8.88E+01
1.21E+03	8.89E+01
1.21E+03	8.89E+01
1.22E+03	8.89E+01
1.22E+03	8.87E+01
1.23E+03	8.85E+01
1.23E+03	8.82E+01
1.23E+03	8.79E+01
1.24E+03	8.75E+01
1.24E+03	8.72E+01
1.25E+03	8.68E+01
1.25E+03	8.66E+01
1.25E+03	8.64E+01
1.26E+03	8.65E+01
1.26E+03	8.67E+01
1.27E+03	8.71E+01
1.27E+03	8.75E+01
1.27E+03	8.81E+01
1.28E+03	8.85E+01
1.28E+03	8.89E+01
1.28E+03	8.92E+01
1.29E+03	8.93E+01
1.29E+03	8.94E+01
1.30E+03	8.94E+01
1.30E+03	8.94E+01
1.30E+03	8.94E+01
1.31E+03	8.94E+01
1.31E+03	8.93E+01
1.32E+03	8.93E+01
1.32E+03	8.93E+01
1.32E+03	8.93E+01
1.33E+03	8.92E+01
1.33E+03	8.92E+01
1.33E+03	8.90E+01
1.34E+03	8.88E+01
1.34E+03	8.85E+01
1.35E+03	8.81E+01
1.35E+03	8.77E+01
1.35E+03	8.73E+01
1.36E+03	8.70E+01
1.36E+03	8.67E+01
1.37E+03	8.64E+01
1.37E+03	8.62E+01
1.37E+03	8.59E+01
1.38E+03	8.55E+01
1.38E+03	8.51E+01
1.38E+03	8.46E+01
1.39E+03	8.41E+01
1.39E+03	8.35E+01
1.40E+03	8.28E+01
1.40E+03	8.22E+01
1.40E+03	8.16E+01
1.41E+03	8.11E+01
1.41E+03	8.07E+01
1.42E+03	8.03E+01
1.42E+03	8.00E+01
1.42E+03	7.96E+01
1.43E+03	7.92E+01
1.43E+03	7.88E+01
1.43E+03	7.83E+01
1.44E+03	7.76E+01
1.44E+03	7.70E+01
1.45E+03	7.63E+01

1.45E+03	7.58E+01
1.45E+03	7.55E+01
1.46E+03	7.56E+01
1.46E+03	7.62E+01
1.47E+03	7.72E+01
1.47E+03	7.88E+01
1.47E+03	8.06E+01
1.48E+03	8.25E+01
1.48E+03	8.44E+01
1.48E+03	8.60E+01
1.49E+03	8.74E+01
1.49E+03	8.83E+01
1.50E+03	8.88E+01
1.50E+03	8.88E+01
1.50E+03	8.85E+01
1.51E+03	8.78E+01
1.51E+03	8.68E+01
1.52E+03	8.54E+01
1.52E+03	8.36E+01
1.52E+03	8.15E+01
1.53E+03	7.91E+01
1.53E+03	7.69E+01
1.54E+03	7.49E+01
1.54E+03	7.34E+01
1.54E+03	7.27E+01
1.55E+03	7.27E+01
1.55E+03	7.32E+01
1.55E+03	7.41E+01
1.56E+03	7.49E+01
1.56E+03	7.57E+01
1.57E+03	7.61E+01
1.57E+03	7.62E+01
1.57E+03	7.61E+01
1.58E+03	7.58E+01
1.58E+03	7.53E+01
1.59E+03	7.48E+01
1.59E+03	7.44E+01
1.59E+03	7.40E+01
1.60E+03	7.37E+01
1.60E+03	7.36E+01
1.60E+03	7.37E+01
1.61E+03	7.40E+01
1.61E+03	7.44E+01
1.62E+03	7.50E+01
1.62E+03	7.55E+01
1.62E+03	7.61E+01
1.63E+03	7.66E+01
1.63E+03	7.72E+01
1.64E+03	7.76E+01
1.64E+03	7.81E+01
1.64E+03	7.84E+01
1.65E+03	7.85E+01
1.65E+03	7.86E+01
1.65E+03	7.87E+01
1.66E+03	7.88E+01
1.66E+03	7.91E+01
1.67E+03	7.95E+01
1.67E+03	8.01E+01
1.67E+03	8.08E+01
1.68E+03	8.15E+01
1.68E+03	8.23E+01
1.69E+03	8.29E+01
1.69E+03	8.35E+01
1.69E+03	8.39E+01
1.70E+03	8.42E+01
1.70E+03	8.43E+01
1.70E+03	8.41E+01
1.71E+03	8.36E+01
1.71E+03	8.29E+01
1.72E+03	8.18E+01
1.72E+03	8.06E+01
1.72E+03	7.93E+01
1.73E+03	7.80E+01
1.73E+03	7.69E+01
1.74E+03	7.64E+01
1.74E+03	7.64E+01
1.74E+03	7.71E+01

1.75E+03	7.85E+01
1.75E+03	8.04E+01
1.75E+03	8.26E+01
1.76E+03	8.48E+01
1.76E+03	8.69E+01
1.77E+03	8.86E+01
1.77E+03	9.00E+01
1.77E+03	9.11E+01
1.78E+03	9.18E+01
1.78E+03	9.22E+01
1.79E+03	9.25E+01
1.79E+03	9.27E+01
1.79E+03	9.28E+01
1.80E+03	9.28E+01
1.80E+03	9.27E+01
1.81E+03	9.26E+01
1.81E+03	9.24E+01
1.81E+03	9.23E+01
1.82E+03	9.23E+01
1.82E+03	9.24E+01
1.82E+03	9.26E+01
1.83E+03	9.29E+01
1.83E+03	9.31E+01
1.84E+03	9.33E+01
1.84E+03	9.34E+01
1.84E+03	9.34E+01
1.85E+03	9.34E+01
1.85E+03	9.34E+01
1.86E+03	9.34E+01
1.86E+03	9.34E+01
1.86E+03	9.34E+01
1.87E+03	9.34E+01
1.87E+03	9.33E+01
1.87E+03	9.32E+01
1.88E+03	9.31E+01
1.88E+03	9.31E+01
1.89E+03	9.31E+01
1.89E+03	9.32E+01
1.89E+03	9.33E+01
1.90E+03	9.34E+01
1.90E+03	9.35E+01
1.91E+03	9.36E+01
1.91E+03	9.37E+01
1.91E+03	9.37E+01
1.92E+03	9.36E+01
1.92E+03	9.36E+01
1.92E+03	9.34E+01
1.93E+03	9.33E+01
1.93E+03	9.32E+01
1.94E+03	9.30E+01
1.94E+03	9.30E+01
1.94E+03	9.29E+01
1.95E+03	9.29E+01
1.95E+03	9.29E+01
1.96E+03	9.28E+01
1.96E+03	9.27E+01
1.96E+03	9.26E+01
1.97E+03	9.25E+01
1.97E+03	9.24E+01
1.97E+03	9.24E+01
1.98E+03	9.25E+01
1.98E+03	9.25E+01
1.99E+03	9.26E+01
1.99E+03	9.27E+01
1.99E+03	9.28E+01
2.00E+03	9.28E+01
2.00E+03	9.28E+01
2.01E+03	9.28E+01
2.01E+03	9.28E+01
2.01E+03	9.28E+01
2.02E+03	9.28E+01
2.02E+03	9.27E+01
2.02E+03	9.26E+01
2.03E+03	9.25E+01
2.03E+03	9.24E+01
2.04E+03	9.23E+01
2.04E+03	9.22E+01

2.04E+03	9.21E+01
2.05E+03	9.20E+01
2.05E+03	9.20E+01
2.06E+03	9.20E+01
2.06E+03	9.20E+01
2.06E+03	9.20E+01
2.07E+03	9.21E+01
2.07E+03	9.22E+01
2.08E+03	9.22E+01
2.08E+03	9.23E+01
2.08E+03	9.23E+01
2.09E+03	9.23E+01
2.09E+03	9.22E+01
2.09E+03	9.22E+01
2.10E+03	9.23E+01
2.10E+03	9.23E+01
2.11E+03	9.24E+01
2.11E+03	9.24E+01
2.11E+03	9.25E+01
2.12E+03	9.26E+01
2.12E+03	9.26E+01
2.13E+03	9.27E+01
2.13E+03	9.28E+01
2.13E+03	9.29E+01
2.14E+03	9.30E+01
2.14E+03	9.30E+01
2.14E+03	9.29E+01
2.15E+03	9.28E+01
2.15E+03	9.26E+01
2.16E+03	9.24E+01
2.16E+03	9.22E+01
2.16E+03	9.19E+01
2.17E+03	9.17E+01
2.17E+03	9.15E+01
2.18E+03	9.12E+01
2.18E+03	9.10E+01
2.18E+03	9.08E+01
2.19E+03	9.06E+01
2.19E+03	9.04E+01
2.19E+03	9.02E+01
2.20E+03	9.00E+01
2.20E+03	8.98E+01
2.21E+03	8.96E+01
2.21E+03	8.94E+01
2.21E+03	8.92E+01
2.22E+03	8.91E+01
2.22E+03	8.89E+01
2.23E+03	8.88E+01
2.23E+03	8.86E+01
2.23E+03	8.84E+01
2.24E+03	8.82E+01
2.24E+03	8.79E+01
2.24E+03	8.76E+01
2.25E+03	8.73E+01
2.25E+03	8.69E+01
2.26E+03	8.65E+01
2.26E+03	8.61E+01
2.26E+03	8.56E+01
2.27E+03	8.52E+01
2.27E+03	8.47E+01
2.28E+03	8.41E+01
2.28E+03	8.33E+01
2.28E+03	8.21E+01
2.29E+03	8.05E+01
2.29E+03	7.83E+01
2.29E+03	7.54E+01
2.30E+03	7.18E+01
2.30E+03	6.75E+01
2.31E+03	6.26E+01
2.31E+03	5.70E+01
2.31E+03	5.11E+01
2.32E+03	4.48E+01
2.32E+03	3.84E+01
2.33E+03	3.23E+01
2.33E+03	2.67E+01
2.33E+03	2.19E+01
2.34E+03	1.80E+01

2.34E+03	1.47E+01
2.35E+03	1.21E+01
2.35E+03	1.03E+01
2.35E+03	1.00E+01
2.36E+03	1.23E+01
2.36E+03	1.79E+01
2.36E+03	2.68E+01
2.37E+03	3.75E+01
2.37E+03	4.83E+01
2.38E+03	5.82E+01
2.38E+03	6.65E+01
2.38E+03	7.31E+01
2.39E+03	7.82E+01
2.39E+03	8.20E+01
2.40E+03	8.47E+01
2.40E+03	8.66E+01
2.40E+03	8.79E+01
2.41E+03	8.87E+01
2.41E+03	8.92E+01
2.41E+03	8.95E+01
2.42E+03	8.99E+01
2.42E+03	9.03E+01
2.43E+03	9.07E+01
2.43E+03	9.11E+01
2.43E+03	9.15E+01
2.44E+03	9.18E+01
2.44E+03	9.20E+01
2.45E+03	9.21E+01
2.45E+03	9.22E+01
2.45E+03	9.22E+01
2.46E+03	9.22E+01
2.46E+03	9.22E+01
2.46E+03	9.22E+01
2.47E+03	9.23E+01
2.47E+03	9.24E+01
2.48E+03	9.25E+01
2.48E+03	9.25E+01
2.48E+03	9.26E+01
2.49E+03	9.26E+01
2.49E+03	9.26E+01
2.50E+03	9.27E+01
2.50E+03	9.27E+01
2.50E+03	9.27E+01
2.51E+03	9.27E+01
2.51E+03	9.27E+01
2.51E+03	9.26E+01
2.52E+03	9.25E+01
2.52E+03	9.25E+01
2.53E+03	9.25E+01
2.53E+03	9.26E+01
2.53E+03	9.26E+01
2.54E+03	9.28E+01
2.54E+03	9.28E+01
2.55E+03	9.29E+01
2.55E+03	9.29E+01
2.55E+03	9.29E+01
2.56E+03	9.28E+01
2.56E+03	9.27E+01
2.56E+03	9.25E+01
2.57E+03	9.24E+01
2.57E+03	9.22E+01
2.58E+03	9.20E+01
2.58E+03	9.18E+01
2.58E+03	9.15E+01
2.59E+03	9.12E+01
2.59E+03	9.09E+01
2.60E+03	9.06E+01
2.60E+03	9.03E+01
2.60E+03	8.99E+01
2.61E+03	8.96E+01
2.61E+03	8.93E+01
2.62E+03	8.91E+01
2.62E+03	8.90E+01
2.62E+03	8.90E+01
2.63E+03	8.90E+01
2.63E+03	8.91E+01
2.63E+03	8.92E+01

2.64E+03	8.92E+01
2.64E+03	8.93E+01
2.65E+03	8.93E+01
2.65E+03	8.93E+01
2.65E+03	8.93E+01
2.66E+03	8.93E+01
2.66E+03	8.93E+01
2.67E+03	8.92E+01
2.67E+03	8.92E+01
2.67E+03	8.92E+01
2.68E+03	8.92E+01
2.68E+03	8.92E+01
2.68E+03	8.92E+01
2.69E+03	8.92E+01
2.69E+03	8.91E+01
2.70E+03	8.91E+01
2.70E+03	8.91E+01
2.70E+03	8.90E+01
2.71E+03	8.91E+01
2.71E+03	8.91E+01
2.72E+03	8.92E+01
2.72E+03	8.92E+01
2.72E+03	8.93E+01
2.73E+03	8.94E+01
2.73E+03	8.94E+01
2.73E+03	8.95E+01
2.74E+03	8.96E+01
2.74E+03	8.97E+01
2.75E+03	8.98E+01
2.75E+03	9.00E+01
2.75E+03	9.02E+01
2.76E+03	9.04E+01
2.76E+03	9.06E+01
2.77E+03	9.09E+01
2.77E+03	9.11E+01
2.77E+03	9.14E+01
2.78E+03	9.16E+01
2.78E+03	9.16E+01
2.78E+03	9.16E+01
2.79E+03	9.15E+01
2.79E+03	9.13E+01
2.80E+03	9.10E+01
2.80E+03	9.06E+01
2.80E+03	9.02E+01
2.81E+03	8.98E+01
2.81E+03	8.92E+01
2.82E+03	8.84E+01
2.82E+03	8.73E+01
2.82E+03	8.56E+01
2.83E+03	8.32E+01
2.83E+03	8.01E+01
2.83E+03	7.63E+01
2.84E+03	7.20E+01
2.84E+03	6.76E+01
2.85E+03	6.37E+01
2.85E+03	6.07E+01
2.85E+03	5.90E+01
2.86E+03	5.88E+01
2.86E+03	5.98E+01
2.87E+03	6.17E+01
2.87E+03	6.39E+01
2.87E+03	6.58E+01
2.88E+03	6.72E+01
2.88E+03	6.76E+01
2.89E+03	6.69E+01
2.89E+03	6.51E+01
2.89E+03	6.21E+01
2.90E+03	5.79E+01
2.90E+03	5.27E+01
2.90E+03	4.67E+01
2.91E+03	4.03E+01
2.91E+03	3.41E+01
2.92E+03	2.90E+01
2.92E+03	2.56E+01
2.92E+03	2.44E+01
2.93E+03	2.55E+01
2.93E+03	2.86E+01

2.94E+03	3.30E+01
2.94E+03	3.79E+01
2.94E+03	4.30E+01
2.95E+03	4.80E+01
2.95E+03	5.28E+01
2.95E+03	5.77E+01
2.96E+03	6.26E+01
2.96E+03	6.77E+01
2.97E+03	7.29E+01
2.97E+03	7.79E+01
2.97E+03	8.24E+01
2.98E+03	8.62E+01
2.98E+03	8.91E+01
2.99E+03	9.12E+01
2.99E+03	9.24E+01
2.99E+03	9.30E+01
3.00E+03	9.32E+01
3.00E+03	9.27E+01
3.00E+03	9.23E+01
3.01E+03	9.19E+01
3.01E+03	9.16E+01
3.02E+03	9.15E+01
3.02E+03	9.15E+01
3.02E+03	9.15E+01
3.03E+03	9.15E+01
3.03E+03	9.14E+01
3.04E+03	9.13E+01
3.04E+03	9.12E+01
3.04E+03	9.11E+01
3.05E+03	9.10E+01
3.05E+03	9.10E+01
3.05E+03	9.10E+01
3.06E+03	9.10E+01
3.06E+03	9.10E+01
3.07E+03	9.09E+01
3.07E+03	9.08E+01
3.07E+03	9.06E+01
3.08E+03	9.04E+01
3.08E+03	9.01E+01
3.09E+03	8.98E+01
3.09E+03	8.95E+01
3.09E+03	8.92E+01
3.10E+03	8.88E+01
3.10E+03	8.86E+01
3.10E+03	8.84E+01
3.11E+03	8.83E+01
3.11E+03	8.84E+01
3.12E+03	8.85E+01
3.12E+03	8.86E+01
3.12E+03	8.87E+01
3.13E+03	8.87E+01
3.13E+03	8.87E+01
3.14E+03	8.86E+01
3.14E+03	8.85E+01
3.14E+03	8.85E+01
3.15E+03	8.84E+01
3.15E+03	8.83E+01
3.16E+03	8.82E+01
3.16E+03	8.81E+01
3.16E+03	8.80E+01
3.17E+03	8.79E+01
3.17E+03	8.79E+01
3.17E+03	8.80E+01
3.18E+03	8.81E+01
3.18E+03	8.83E+01
3.19E+03	8.84E+01
3.19E+03	8.85E+01
3.19E+03	8.87E+01
3.20E+03	8.88E+01
3.20E+03	8.90E+01
3.21E+03	8.93E+01
3.21E+03	8.95E+01
3.21E+03	8.96E+01
3.22E+03	8.98E+01
3.22E+03	8.99E+01
3.22E+03	9.00E+01
3.23E+03	9.01E+01

3.23E+03	9.03E+01
3.24E+03	9.05E+01
3.24E+03	9.07E+01
3.24E+03	9.08E+01
3.25E+03	9.09E+01
3.25E+03	9.09E+01
3.26E+03	9.10E+01
3.26E+03	9.10E+01
3.26E+03	9.10E+01
3.27E+03	9.11E+01
3.27E+03	9.11E+01
3.27E+03	9.10E+01
3.28E+03	9.08E+01
3.28E+03	9.06E+01
3.29E+03	9.03E+01
3.29E+03	9.00E+01
3.29E+03	8.99E+01
3.30E+03	8.99E+01
3.30E+03	9.00E+01
3.31E+03	9.02E+01
3.31E+03	9.05E+01
3.31E+03	9.07E+01
3.32E+03	9.09E+01
3.32E+03	9.10E+01
3.32E+03	9.11E+01
3.33E+03	9.12E+01
3.33E+03	9.12E+01
3.34E+03	9.11E+01
3.34E+03	9.10E+01
3.34E+03	9.09E+01
3.35E+03	9.09E+01
3.35E+03	9.10E+01
3.36E+03	9.12E+01
3.36E+03	9.15E+01
3.36E+03	9.18E+01
3.37E+03	9.19E+01
3.37E+03	9.20E+01
3.37E+03	9.20E+01
3.38E+03	9.20E+01
3.38E+03	9.20E+01
3.39E+03	9.22E+01
3.39E+03	9.24E+01
3.39E+03	9.28E+01
3.40E+03	9.31E+01
3.40E+03	9.34E+01
3.41E+03	9.36E+01
3.41E+03	9.36E+01
3.41E+03	9.36E+01
3.42E+03	9.35E+01
3.42E+03	9.33E+01
3.43E+03	9.29E+01
3.43E+03	9.23E+01
3.43E+03	9.16E+01
3.44E+03	9.13E+01
3.44E+03	9.09E+01
3.44E+03	9.06E+01
3.45E+03	9.04E+01
3.45E+03	9.04E+01
3.46E+03	9.03E+01
3.46E+03	9.01E+01
3.46E+03	8.99E+01
3.47E+03	8.97E+01
3.47E+03	8.94E+01
3.48E+03	8.92E+01
3.48E+03	8.92E+01
3.48E+03	8.91E+01
3.49E+03	8.92E+01
3.49E+03	8.91E+01
3.49E+03	8.90E+01
3.50E+03	8.88E+01
3.50E+03	8.86E+01
3.51E+03	8.85E+01
3.51E+03	8.84E+01
3.51E+03	8.84E+01
3.52E+03	8.84E+01
3.52E+03	8.84E+01
3.53E+03	8.83E+01

3.53E+03	8.82E+01
3.53E+03	8.80E+01
3.54E+03	8.78E+01
3.54E+03	8.75E+01
3.54E+03	8.72E+01
3.55E+03	8.68E+01
3.55E+03	8.63E+01
3.56E+03	8.57E+01
3.56E+03	8.51E+01
3.56E+03	8.46E+01
3.57E+03	8.43E+01
3.57E+03	8.42E+01
3.58E+03	8.44E+01
3.58E+03	8.46E+01
3.58E+03	8.49E+01
3.59E+03	8.52E+01
3.59E+03	8.54E+01
3.59E+03	8.55E+01
3.60E+03	8.57E+01
3.60E+03	8.60E+01
3.61E+03	8.62E+01
3.61E+03	8.63E+01
3.61E+03	8.62E+01
3.62E+03	8.59E+01
3.62E+03	8.56E+01
3.63E+03	8.54E+01
3.63E+03	8.54E+01
3.63E+03	8.58E+01
3.64E+03	8.64E+01
3.64E+03	8.73E+01
3.64E+03	8.82E+01
3.65E+03	8.90E+01
3.65E+03	8.97E+01
3.66E+03	9.03E+01
3.66E+03	9.09E+01
3.66E+03	9.14E+01
3.67E+03	9.18E+01
3.67E+03	9.17E+01
3.68E+03	9.12E+01
3.68E+03	8.99E+01
3.68E+03	8.82E+01
3.69E+03	8.61E+01
3.69E+03	8.42E+01
3.70E+03	8.25E+01
3.70E+03	8.14E+01
3.70E+03	8.07E+01
3.71E+03	8.05E+01
3.71E+03	8.07E+01
3.71E+03	8.12E+01
3.72E+03	8.21E+01
3.72E+03	8.33E+01
3.73E+03	8.50E+01
3.73E+03	8.70E+01
3.73E+03	8.93E+01
3.74E+03	9.16E+01
3.74E+03	9.33E+01
3.75E+03	9.42E+01
3.75E+03	9.41E+01
3.75E+03	9.33E+01
3.76E+03	9.20E+01
3.76E+03	9.07E+01
3.76E+03	8.95E+01
3.77E+03	8.86E+01
3.77E+03	8.80E+01
3.78E+03	8.78E+01
3.78E+03	8.81E+01
3.78E+03	8.88E+01
3.79E+03	9.00E+01
3.79E+03	9.15E+01
3.80E+03	9.32E+01
3.80E+03	9.48E+01
3.80E+03	9.62E+01
3.81E+03	9.72E+01
3.81E+03	9.79E+01
3.81E+03	9.82E+01
3.82E+03	9.83E+01
3.82E+03	9.82E+01

3.83E+03	9.80E+01
3.83E+03	9.76E+01
3.83E+03	9.70E+01
3.84E+03	9.64E+01
3.84E+03	9.59E+01
3.85E+03	9.55E+01
3.85E+03	9.56E+01
3.85E+03	9.59E+01
3.86E+03	9.63E+01
3.86E+03	9.65E+01
3.86E+03	9.66E+01
3.87E+03	9.64E+01
3.87E+03	9.60E+01
3.88E+03	9.57E+01
3.88E+03	9.54E+01
3.88E+03	9.54E+01
3.89E+03	9.54E+01
3.89E+03	9.54E+01
3.90E+03	9.52E+01
3.90E+03	9.48E+01
3.90E+03	9.42E+01
3.91E+03	9.34E+01
3.91E+03	9.25E+01
3.91E+03	9.17E+01
3.92E+03	9.10E+01
3.92E+03	9.05E+01
3.93E+03	9.02E+01
3.93E+03	9.02E+01
3.93E+03	9.04E+01
3.94E+03	9.09E+01
3.94E+03	9.14E+01
3.95E+03	9.20E+01
3.95E+03	9.25E+01
3.95E+03	9.30E+01
3.96E+03	9.33E+01
3.96E+03	9.35E+01
3.96E+03	9.38E+01
3.97E+03	9.40E+01
3.97E+03	9.43E+01
3.98E+03	9.45E+01
3.98E+03	9.45E+01
3.98E+03	9.43E+01
3.99E+03	9.39E+01
3.99E+03	9.34E+01
4.00E+03	9.27E+01
4.00E+03	9.22E+01
4.00E+03	0.00E+00

2.2 Raman chart data

Raman shift (cm^{-1})	Intensity (counts)
50.292	7.22155
50.8191	20.6681
51.3441	32.9511
51.8711	45.0337
52.3961	54.8506
52.9231	62.4066
53.4479	68.4217
53.9749	72.753
54.5018	75.4469
55.0266	76.7689
55.5535	76.7213
56.0782	75.4444
56.605	73.1097
57.1296	69.9456
57.6564	65.0412
58.181	59.5174
58.7077	53.4206
59.2322	46.9689
59.7588	40.5906

60.2833	34.1806
60.8099	27.7071
61.3343	20.6713
61.8608	13.525
62.3852	6.37149
62.9116	2.41647
63.4359	2.2922
63.9623	2.7567
64.4865	3.41908
65.0129	4.08767
65.5371	4.89588
66.0633	5.73358
66.5875	6.572
67.1137	7.24831
67.6377	7.48496
68.1618	7.53825
68.6879	7.46664
69.2119	7.94263
69.738	9.12049
70.2619	8.99477
70.7879	8.54637
71.3117	7.5005
71.8356	6.15115
72.3615	4.76924
72.8852	3.44501
73.409	2.38628
73.9348	1.71929
74.4585	1.41921
74.9843	1.35708
75.5079	1.82216
76.0315	5.21475
76.5572	6.29539
77.0807	7.4082
77.6042	8.31787
78.1298	8.93901
78.6532	9.04247
79.1767	8.9048
79.7022	8.74421
80.2255	8.47001
80.7488	8.09428
81.2743	7.60059
81.7975	7.06185
82.3208	6.48867
82.844	5.87192
83.3693	5.37809
83.8924	5.22123
84.4156	4.90492
84.9408	4.59997
85.4638	4.31946
85.9869	4.12703
86.5099	3.97148
87.035	4.13329
87.5579	4.26296
88.0808	4.30799
88.6037	4.61731
89.1266	5.12271
89.6515	6.22641
90.1743	7.51579
90.6971	8.78285
91.2198	10.0779
91.7447	11.3098
92.2673	11.8007

92.79	12.4182
93.3126	12.9267
93.8352	13.0321
94.3578	13.0287
94.8824	12.9711
95.4049	12.9238
95.9274	12.9208
96.4499	12.9398
96.9723	13.3088
97.4947	13.7655
98.0191	14.169
98.5415	14.8336
99.0638	16.0054
99.586	17.2049
100.108	18.4549
100.63	19.5767
101.153	20.6009
101.675	20.8074
102.197	20.9975
102.721	20.8272
103.243	20.2192
103.765	19.4582
104.287	18.2358
104.809	16.9858
105.331	15.8765
105.853	15.8268
106.375	16.4655
106.897	17.3715
107.419	17.7463
107.941	18.1791
108.462	18.2119
108.984	18.0792
109.506	17.0502
110.027	16.2069
110.549	15.5629
111.071	14.9997
111.592	14.4704
112.114	13.9961
112.635	13.5936
113.157	13.2703
113.678	12.693
114.2	12.1998
114.721	11.7041
115.243	11.2966
115.764	10.9513
116.285	10.7387
116.807	10.793
117.328	11.5222
117.849	11.5591
118.37	11.4342
118.892	11.0019
119.413	10.5919
119.934	10.1947
120.455	9.7963
120.976	9.49095
121.495	9.24294
122.016	9.00957
122.537	8.75894
123.058	8.48239
123.579	8.60741
124.1	8.74566
124.621	8.96966

125.142	9.27759
125.662	9.55372
126.181	9.89461
126.702	10.1972
127.222	10.5586
127.743	10.8324
128.264	10.8381
128.784	10.0222
129.303	9.38874
129.824	9.15006
130.344	9.20531
130.865	9.21215
131.385	9.19641
131.903	9.12353
132.424	8.64698
132.944	7.89048
133.465	6.90644
133.985	5.84117
134.503	4.59582
135.023	3.39898
135.544	2.23585
136.064	1.1961
136.582	0.194537
137.102	-0.370807
137.622	-0.751853
138.143	-1.03545
138.66	-0.727815
139.181	-0.00938225
139.701	0.934563
140.221	2.06616
140.738	3.18052
141.258	4.40112
141.778	5.25184
142.296	6.07324
142.816	6.77967
143.336	7.31853
143.855	7.66435
144.373	7.79303
144.893	7.96273
145.413	7.19112
145.93	6.30875
146.45	5.30066
146.969	4.25974
147.487	3.249
148.006	2.29834
148.524	2.23172
149.043	1.92596
149.563	1.91278
150.08	1.9292
150.6	2.12435
151.119	2.46471
151.636	2.88977
152.156	3.52681
152.673	4.40169
153.192	5.43677
153.711	6.59535
154.228	7.75595
154.748	9.1861
155.265	10.5103
155.784	11.5859
156.301	11.7398
156.82	11.1535

157.339	10.3694
157.856	9.3999
158.375	7.87221
158.892	6.35951
159.411	4.89506
159.927	3.57216
160.446	2.52444
160.963	1.92933
161.482	1.46377
161.998	1.19327
162.517	1.30733
163.034	1.80759
163.552	2.94882
164.069	3.30141
164.588	3.17398
165.104	3.09436
165.623	3.15975
166.139	3.22122
166.658	3.31187
167.174	3.37213
167.69	3.47029
168.209	3.74993
168.725	4.00634
169.244	4.43489
169.76	5.01785
170.278	5.64895
170.794	6.27655
171.31	6.67291
171.829	6.89503
172.345	6.32862
172.863	5.92976
173.379	5.35635
173.895	4.89272
174.413	4.03797
174.929	3.37541
175.445	2.96739
175.963	2.87967
176.479	3.15802
176.997	3.30962
177.513	3.87041
178.028	4.48971
178.546	5.12339
179.062	5.7886
179.578	6.3956
180.093	6.8965
180.611	7.26133
181.127	7.45824
181.642	7.56807
182.16	7.59986
182.676	7.40796
183.191	7.25384
183.709	7.07401
184.224	6.89142
184.739	6.80993
185.255	6.91119
185.772	7.55155
186.288	8.48267
186.803	9.12998
187.318	9.66887
187.835	9.86979
188.351	10.0273
188.866	10.1261

189.381	10.2161
189.898	10.2548
190.413	10.3092
190.928	10.2677
191.443	10.4036
191.958	10.5887
192.476	10.7359
192.991	10.7849
193.505	10.5425
194.02	10.0156
194.535	8.92928
195.05	7.97821
195.567	7.26759
196.082	6.26771
196.596	5.45311
197.111	4.95098
197.626	4.87484
198.141	5.33804
198.655	5.89672
199.172	6.62978
199.686	7.49701
200.201	8.30681
200.716	9.44969
201.23	10.5649
201.745	11.5779
202.259	12.5017
202.773	13.1925
203.288	13.0245
203.802	12.6738
204.319	12.2307
204.833	11.7094
205.347	11.1269
205.861	10.766
206.376	10.2429
206.89	9.6804
207.404	9.00479
207.918	8.44977
208.432	7.79515
208.946	6.96315
209.46	6.18971
209.974	5.47924
210.488	4.86717
211.002	4.3779
211.516	4.08251
212.03	4.2206
212.544	4.54691
213.058	5.20431
213.572	6.24534
214.085	7.48364
214.599	9.27881
215.113	11.1948
215.626	13.123
216.14	14.2615
216.654	15.4413
217.167	16.4021
217.681	17.1519
218.192	17.6982
218.706	17.6436
219.219	16.9744
219.733	16.0668
220.246	15.0729
220.76	13.9125

221.273	12.953
221.787	12.0232
222.3	11.2428
222.813	10.7086
223.324	10.5914
223.838	10.7891
224.351	11.6947
224.864	12.7127
225.377	13.7123
225.89	14.6623
226.403	15.3506
226.914	16.0601
227.427	16.7292
227.94	17.3084
228.453	17.8545
228.966	18.3207
229.479	18.7502
229.99	19.1027
230.503	19.3451
231.016	19.5114
231.529	19.6186
232.039	19.6974
232.552	19.6582
233.065	19.5754
233.577	19.4264
234.09	19.1722
234.601	18.8769
235.113	18.4286
235.626	17.9012
236.139	17.2405
236.649	16.4317
237.161	15.4343
237.674	14.2373
238.184	13.0211
238.697	11.7402
239.209	10.3155
239.721	8.99617
240.232	7.51224
240.744	6.36715
241.256	6.25334
241.766	5.84407
242.279	5.42217
242.791	5.19241
243.301	4.87949
243.813	4.65643
244.325	4.62178
244.835	4.64081
245.347	4.72352
245.859	4.87309
246.369	5.04406
246.881	5.25697
247.393	5.5047
247.903	5.83394
248.415	6.15401
248.925	6.57162
249.436	6.97841
249.948	7.44629
250.458	7.94519
250.97	8.44364
251.479	8.90785
251.991	8.7512
252.5	8.42613

253.012	7.68664
253.524	7.05613
254.033	6.3937
254.545	5.81762
255.054	5.26119
255.566	4.75904
256.075	4.35455
256.586	3.98994
257.096	3.82637
257.607	3.69911
258.116	3.79826
258.628	3.92599
259.137	4.46602
259.648	4.96163
260.157	5.58118
260.668	6.21425
261.178	6.83684
261.689	7.35672
262.198	6.82817
262.709	6.30495
263.218	6.08413
263.729	6.15074
264.238	6.18265
264.749	6.33128
265.258	6.41906
265.766	6.4737
266.277	6.44897
266.786	6.34763
267.297	6.0594
267.806	5.77395
268.314	5.29682
268.825	4.85495
269.334	4.32401
269.845	3.75969
270.353	3.19608
270.862	2.71801
271.372	2.16732
271.881	1.72799
272.391	1.24894
272.9	0.847536
273.408	0.954735
273.919	0.972018
274.427	1.45434
274.935	1.86093
275.446	2.36272
275.954	2.97261
276.462	3.69184
276.973	4.42124
277.481	5.01248
277.989	5.42432
278.497	5.45552
279.007	5.27131
279.515	5.00547
280.023	4.77538
280.534	4.47704
281.042	4.20977
281.549	3.89887
282.057	3.54938
282.567	3.30195
283.075	3.35012
283.583	3.57432
284.091	3.81734

284.601	4.20579
285.109	5.50311
285.616	6.68054
286.124	7.65572
286.632	8.30436
287.142	8.73106
287.649	8.98803
288.157	9.19515
288.664	9.42992
289.172	10.0302
289.682	10.1563
290.189	10.5513
290.696	10.2048
291.204	10.0527
291.711	10.1356
292.219	10.7537
292.728	11.6671
293.235	12.7815
293.743	14.0295
294.25	15.405
294.757	16.568
295.264	17.9685
295.772	19.191
296.279	19.5257
296.786	19.3926
297.295	20.2226
297.802	21.512
298.309	23.3844
298.816	25.9302
299.323	28.7425
299.83	31.1216
300.337	33.4254
300.844	35.1666
301.351	36.7244
301.858	38.1243
302.365	39.5612
302.871	41.4043
303.378	43.0098
303.885	43.8148
304.392	43.76
304.898	42.6221
305.405	41.9081
305.912	40.9122
306.418	39.6585
306.925	38.0419
307.431	36.3322
307.938	34.3318
308.444	32.0894
308.951	29.544
309.457	26.555
309.964	22.9729
310.47	20.3939
310.976	18.4037
311.483	17.2609
311.989	15.8759
312.495	14.4338
313.002	13.1918
313.508	12.2095
314.014	11.6669
314.52	11.3425
315.024	11.1788
315.53	11.2971

316.036	10.9678
316.542	10.2281
317.048	9.54686
317.554	9.01146
318.06	8.28922
318.566	6.76744
319.072	4.97938
319.576	3.33875
320.082	2.04356
320.588	1.25027
321.094	0.694299
321.599	0.67748
322.105	0.60445
322.609	0.863019
323.114	1.17281
323.62	1.65924
324.126	1.4647
324.631	-0.712963
325.135	-1.16457
325.64	-1.34998
326.146	-1.38174
326.651	-1.22096
327.157	-0.835575
327.66	-0.247749
328.166	0.603693
328.671	1.85385
329.176	3.07256
329.68	3.75031
330.185	4.30099
330.69	4.64849
331.195	4.78235
331.698	4.67173
332.204	4.37541
332.709	3.85157
333.212	3.00759
333.717	1.78414
334.222	1.48904
334.727	1.46729
335.23	1.64723
335.735	1.59663
336.24	1.46645
336.743	1.329
337.248	1.58665
337.753	2.09468
338.255	2.52893
338.76	2.49426
339.263	2.23917
339.768	1.90742
340.273	1.51245
340.775	1.22325
341.28	0.844782
341.785	0.560999
342.287	0.452354
342.792	0.31485
343.294	0.597575
343.799	1.21393
344.303	2.12407
344.806	4.12796
345.31	6.2986
345.813	7.36166
346.317	8.30025
346.819	9.15913

347.324	9.68528
347.828	10.1145
348.33	10.5387
348.835	10.9777
349.337	11.3243
349.841	11.4426
350.343	11.2249
350.847	10.9706
351.349	10.6642
351.853	10.4058
352.355	10.2088
352.859	10.1123
353.361	10.0237
353.865	9.93074
354.367	9.6727
354.871	9.38651
355.373	8.88442
355.877	8.32488
356.379	7.76551
356.881	7.43028
357.384	7.14036
357.886	6.83675
358.39	6.2382
358.892	5.52766
359.395	4.56001
359.897	3.58717
360.398	2.76798
360.902	2.52651
361.404	2.5133
361.907	2.77363
362.409	3.0929
362.91	3.34634
363.414	3.55109
363.915	3.4967
364.418	3.1536
364.92	2.53441
365.421	2.32202
365.924	2.31989
366.426	2.33296
366.927	2.4436
367.43	2.53314
367.931	2.61884
368.432	2.7295
368.936	2.88764
369.437	3.01789
369.938	3.20704
370.441	3.30753
370.942	3.49118
371.443	3.52658
371.944	3.63455
372.447	3.74629
372.948	3.8649
373.448	4.3735
373.949	4.86269
374.452	5.48681
374.953	5.98665
375.454	6.60727
375.954	7.22856
376.457	7.95601
376.958	8.70071
377.458	9.57594
377.959	9.36608

378.462	5.20076
378.962	4.06097
379.463	3.01004
379.963	2.10574
380.464	1.36048
380.966	0.839102
381.467	0.418783
381.967	0.202199
382.468	0.150864
382.968	0.168924
383.468	0.318454
383.971	0.657515
384.471	1.01075
384.971	1.66036
385.471	2.33992
385.972	3.04529
386.472	3.77607
386.972	4.47573
387.472	5.20676
387.974	5.82188
388.474	6.32695
388.974	6.73303
389.474	7.03491
389.974	7.48157
390.474	7.48154
390.974	7.36946
391.474	7.25422
391.974	7.02207
392.474	6.74479
392.973	6.45294
393.473	6.14636
393.973	5.84206
394.473	5.4908
394.972	5.15424
395.472	4.77676
395.972	4.57063
396.471	4.31292
396.971	4.0131
397.47	3.81738
397.97	3.57279
398.469	3.36214
398.969	3.14334
399.468	2.99591
399.968	2.81254
400.467	2.72316
400.966	2.57131
401.466	2.45412
401.965	2.33806
402.464	2.19002
402.964	2.37022
403.463	2.64094
403.962	3.14957
404.461	3.86056
404.96	4.66256
405.46	5.3423
405.959	6.00266
406.458	6.63658
406.955	7.28804
407.454	7.88055
407.953	8.54918
408.452	8.83798
408.951	8.9867

409.45	9.13887
409.948	9.15655
410.447	9.16797
410.944	9.1812
411.443	9.23473
411.942	9.27361
412.44	9.34044
412.939	9.64794
413.438	10.09
413.934	10.6042
414.433	11.1219
414.932	11.603
415.43	12.0782
415.929	12.505
416.425	12.9131
416.924	13.2262
417.422	13.4676
417.921	13.3152
418.417	12.9294
418.915	12.488
419.414	12.0259
419.912	11.4045
420.408	10.7666
420.907	10.2172
421.405	9.7624
421.903	10.0362
422.399	10.492
422.897	10.9456
423.396	11.3273
423.892	10.8668
424.39	9.87268
424.888	9.33797
425.386	9.30223
425.882	9.30959
426.38	9.08458
426.878	8.82193
427.374	8.27661
427.872	7.71379
428.37	7.30505
428.865	7.14025
429.363	7.38627
429.859	7.79114
430.357	8.41139
430.854	9.22697
431.35	10.1707
431.848	11.4664
432.345	13.0333
432.841	14.333
433.339	15.0865
433.834	15.6905
434.332	16.0745
434.827	16.3033
435.325	16.38
435.822	16.3211
436.318	16.1648
436.815	15.9632
437.31	15.5034
437.808	15.0263
438.303	14.4564
438.8	14.2972
439.296	14.69
439.793	14.9996

440.288	15.1432
440.785	15.2704
441.282	15.2469
441.777	15.0114
442.273	14.5494
442.77	13.5488
443.265	12.1883
443.762	10.7055
444.257	9.12248
444.754	8.03545
445.249	7.07442
445.746	6.22676
446.24	5.52614
446.737	4.83208
447.232	4.14861
447.729	3.36646
448.224	2.66126
448.718	2.13962
449.215	1.44585
449.71	1.63506
450.206	1.78024
450.701	2.29235
451.196	3.54609
451.692	4.06069
452.187	3.84055
452.683	3.09874
453.178	2.07669
453.672	0.79405
454.169	-0.514055
454.663	-1.81766
455.158	-3.10821
455.654	-2.97064
456.148	-2.86504
456.643	-2.58562
457.139	-2.29797
457.633	-1.93935
458.127	-1.5533
458.624	-1.09714
459.118	-0.64264
459.612	-0.0782205
460.108	0.459727
460.602	1.08954
461.096	1.7225
461.59	2.17696
462.086	2.24804
462.58	1.3889
463.074	1.11264
463.568	1.2867
464.064	1.60302
464.558	1.88379
465.052	1.96566
465.546	2.04034
466.042	1.98724
466.535	1.93299
467.029	1.93723
467.523	1.99272
468.016	2.22237
468.512	2.529
469.006	2.99122
469.499	3.51057
469.993	4.15174
470.487	4.52976

470.98	3.82571
471.476	2.91511
471.969	2.05096
472.463	1.99581
472.956	2.0218
473.449	2.09472
473.943	1.97075
474.436	1.49271
474.931	1.21097
475.425	1.13225
475.918	1.44299
476.411	1.98957
476.904	2.81947
477.398	3.86443
477.891	5.04498
478.384	6.23727
478.877	7.34137
479.37	8.34989
479.863	9.17136
480.358	9.56368
480.851	9.97624
481.344	10.4751
481.837	11.2827
482.33	12.0401
482.823	12.8682
483.316	13.6665
483.809	14.5127
484.301	15.163
484.794	16.5314
485.287	17.8094
485.78	18.6831
486.272	18.9555
486.765	19.1291
487.258	19.2655
487.75	19.5296
488.243	19.8112
488.735	20.0618
489.228	20.353
489.72	20.5839
490.211	20.8351
490.703	21.0048
491.196	21.2611
491.688	21.6537
492.181	22.5582
492.673	24.3752
493.165	26.2585
493.658	28.098
494.15	29.8381
494.642	31.3808
495.134	32.4529
495.625	33.1767
496.117	33.4152
496.609	33.3752
497.101	32.3194
497.593	34.35
498.085	36.6851
498.577	39.1256
499.067	41.6365
499.559	44.2503
500.051	47.0892
500.543	50.2777
501.035	53.9931

501.527	57.9596
502.017	62.0421
502.508	66.1928
503	70.2266
503.492	74.2186
503.984	78.3095
504.473	82.6321
504.965	87.8776
505.457	93.2617
505.948	101.623
506.438	111.595
506.93	119.767
507.421	126.763
507.913	135.795
508.402	144.497
508.894	155.55
509.385	167.25
509.875	182.412
510.366	198.453
510.857	217.821
511.349	238.802
511.838	264.807
512.329	296.45
512.82	328.356
513.31	366.299
513.801	409.033
514.292	462.629
514.781	525.271
515.272	602.068
515.763	693.044
516.253	843.238
516.744	1010.91
517.233	1207.69
517.724	1480.82
518.215	1831.79
518.704	2312.34
519.194	2965.15
519.685	3856.16
520.174	5007.63
520.665	6354.25
521.154	7576.23
521.645	7919.17
522.133	7602.41
522.624	6249.47
523.115	4962.83
523.604	3835.08
524.094	2950.43
524.583	2276.98
525.073	1836.66
525.562	1475.89
526.052	1218.15
526.541	1022.76
527.031	849.298
527.52	728.847
528.01	632.148
528.499	558.903
528.989	493.683
529.478	442.48
529.968	394.198
530.456	355.14
530.947	319.325
531.435	285.891

531.925	261.312
532.413	240.501
532.901	222.949
533.391	206.75
533.88	193.924
534.37	181.666
534.858	172.175
535.348	163.295
535.836	153.837
536.324	145.321
536.814	136.312
537.302	126.707
537.792	119.127
538.28	108.996
538.767	102.312
539.257	96.607
539.745	92.3955
540.233	89.0386
540.723	85.9524
541.21	83.1334
541.698	80.4189
542.188	78.1124
542.675	76.0219
543.163	73.6776
543.653	71.124
544.14	67.6283
544.628	64.7424
545.117	61.6973
545.605	58.8501
546.092	56.4422
546.58	54.0912
547.069	51.8465
547.556	49.4189
548.044	47.2978
548.533	45.6996
549.02	44.2691
549.508	43.0066
549.995	42.0076
550.484	41.2531
550.971	40.6426
551.458	40.2529
551.946	40.011
552.433	39.848
552.922	39.7938
553.409	39.8807
553.896	40.0942
554.383	40.2417
554.87	40.1465
555.359	39.6738
555.846	38.9257
556.333	37.9606
556.82	36.8728
557.307	35.683
557.793	34.4008
558.28	33.0483
558.769	31.7285
559.256	30.4663
559.742	29.3285
560.229	28.3861
560.716	27.8436
561.202	28.0104
561.689	28.3772

562.176	28.8848
562.662	29.2389
563.151	29.4784
563.637	29.665
564.124	29.8179
564.61	28.6707
565.097	26.4332
565.583	24.1473
566.07	21.7935
566.556	19.3806
567.042	16.8774
567.45	15.4267
567.942	14.8845
568.434	14.3414
568.924	13.8195
569.417	13.2923
569.909	12.7992
570.401	12.3756
570.891	11.9207
571.384	11.5181
571.876	11.113
572.366	10.771
572.858	10.4634
573.35	10.1533
573.84	9.89334
574.332	9.74707
574.824	9.65038
575.314	9.54416
575.806	9.42851
576.298	9.33269
576.788	9.21769
577.28	9.35672
577.771	9.61266
578.261	9.82801
578.753	9.86164
579.243	9.66045
579.734	9.15377
580.226	8.79135
580.716	8.49973
581.208	8.46087
581.697	8.46822
582.189	8.55368
582.68	8.79983
583.17	8.93092
583.661	9.44343
584.151	9.95072
584.642	10.4977
585.132	10.9909
585.623	11.5081
586.112	11.9729
586.604	12.3976
587.095	12.7467
587.584	13.0088
588.076	13.0426
588.565	12.8902
589.056	12.4431
589.545	11.7517
590.036	11.086
590.526	10.2454
591.017	9.3461
591.506	8.36772
591.997	7.4635

592.486	8.61677
592.977	8.32438
593.466	8.02344
593.955	7.74455
594.446	7.44218
594.935	7.04041
595.426	6.60674
595.914	6.27035
596.405	5.9404
596.894	6.2777
597.385	6.81103
597.874	7.40666
598.362	7.69746
598.853	7.90317
599.342	8.11684
599.832	8.27221
600.321	8.42956
600.809	8.56397
601.3	8.69739
601.788	8.86458
602.277	9.01787
602.767	9.202
603.256	9.3692
603.746	9.53783
604.235	9.74742
604.723	9.74561
605.213	10.0978
605.702	10.4618
606.19	10.8366
606.68	11.2655
607.169	11.7036
607.657	12.1587
608.145	12.6008
608.635	12.9574
609.123	13.3157
609.611	13.6263
610.101	13.8575
610.589	13.7498
611.077	13.7046
611.565	13.7109
612.055	13.6132
612.543	13.5407
613.031	13.3565
613.519	13.1878
614.009	13.0344
614.497	13.0611
614.985	13.6423
615.473	14.3211
615.962	15.2164
616.45	16.08
616.938	16.7974
617.425	17.5236
617.913	18.1725
618.403	18.7839
618.89	19.3294
619.378	19.8226
619.865	20.3441
620.353	20.5879
620.84	20.6916
621.33	20.6464
621.817	20.4612
622.305	20.1218

622.792	19.666
623.28	19.1952
623.767	18.621
624.254	17.9981
624.744	17.3583
625.231	16.7988
625.718	16.1929
626.205	15.4199
626.692	14.6156
627.18	13.851
627.667	13.13
628.154	12.5577
628.641	12.2156
629.128	12.666
629.617	13.0863
630.104	13.5169
630.591	13.8866
631.078	14.1524
631.565	14.3101
632.052	14.3825
632.539	14.3178
633.025	14.1587
633.512	13.8323
633.999	13.3825
634.486	12.7412
634.973	11.9722
635.459	10.9114
635.946	9.77577
636.433	8.73316
636.919	7.81756
637.406	7.75095
637.892	7.70169
638.379	7.98836
638.865	8.48618
639.352	9.06648
639.839	9.77263
640.325	10.3345
640.811	10.7471
641.298	11.1602
641.784	11.3811
642.268	11.2078
642.755	9.68872
643.241	8.60772
643.727	8.35806
644.214	8.43367
644.7	8.72517
645.186	9.30964
645.672	10.209
646.158	12.5631
646.644	13.9384
647.13	14.7462
647.615	15.1724
648.101	15.3292
648.587	15.525
649.073	15.6659
649.559	15.4982
650.044	15.2855
650.528	15.0513
651.014	14.8377
651.5	14.5891
651.986	14.1795
652.472	13.7386

652.958	13.3208
653.441	12.935
653.927	12.6166
654.413	12.7884
654.898	12.9163
655.384	13.0319
655.868	12.7083
656.353	12.0374
656.839	10.8746
657.324	10.467
657.808	10.5245
658.293	11.1175
658.779	11.7508
659.264	12.3004
659.748	12.7543
660.233	13.1488
660.718	13.6136
661.204	14.0815
661.687	14.4662
662.172	14.6208
662.658	13.2903
663.141	13.1553
663.626	13.0588
664.111	12.8163
664.594	12.5903
665.079	12.3281
665.565	12.0938
666.048	11.7178
666.533	11.737
667.018	11.8174
667.501	11.9072
667.986	12.0708
668.471	12.2096
668.953	12.2836
669.438	12.3832
669.923	12.4652
670.406	12.496
670.891	12.5838
671.374	12.5488
671.858	12.6143
672.343	12.7317
672.826	12.8142
673.311	12.9378
673.793	13.0208
674.278	13.1205
674.762	13.1971
675.245	13.2594
675.73	13.319
676.212	13.3672
676.697	13.454
677.179	13.2994
677.664	13.1618
678.146	12.9609
678.63	12.7802
679.113	12.564
679.597	12.2203
680.079	11.9635
680.564	11.75
681.046	11.6686
681.53	11.6519
682.012	12.3402
682.497	12.854

682.979	13.355
683.463	13.7176
683.945	14.0076
684.429	14.1832
684.911	13.7979
685.395	13.4219
685.877	12.8906
686.361	12.2168
686.843	11.4331
687.325	10.4407
687.809	9.46146
688.291	8.54523
688.775	7.78763
689.257	7.58797
689.74	7.27513
690.222	7.37975
690.704	7.59485
691.188	7.85132
691.669	8.11262
692.151	8.35963
692.635	8.56325
693.116	8.6977
693.6	8.63375
694.082	8.46252
694.563	8.08685
695.047	7.60439
695.528	6.99699
696.01	6.0765
696.493	5.17833
696.974	4.31488
697.456	3.54898
697.939	2.90603
698.421	2.98007
698.902	3.25789
699.383	3.55821
699.866	3.9337
700.348	4.26866
700.829	4.79484
701.312	5.09729
701.793	5.26843
702.274	5.33416
702.755	5.36001
703.239	5.33507
703.72	5.24838
704.201	5.14946
704.682	5.0178
705.163	4.83621
705.646	4.64819
706.127	4.434
706.607	4.22229
707.088	3.98045
707.571	3.75066
708.052	3.48696
708.533	3.34213
709.014	3.03348
709.494	2.77344
709.975	2.4986
710.458	2.21092
710.939	1.9666
711.419	1.69379
711.9	1.43727
712.38	1.18109

712.861	0.943233
713.342	0.727078
713.824	0.516272
714.305	0.164283
714.785	0
715.266	-0.0306878
715.746	-0.030726
716.226	0.000927925
716.707	0.106123
717.187	0.332901
717.667	0.572154
718.15	0.730289
718.63	0.899186
719.11	1.06231
719.59	1.2173
720.071	1.37013
720.551	1.50698
721.031	1.6395
721.511	1.60121
721.991	1.59073
722.471	1.61294
722.951	1.62819
723.431	1.64332
723.911	1.70884
724.391	1.64383
724.871	1.53881
725.351	1.37941
725.831	1.22503
726.311	1.02692
726.791	0.832644
727.27	0.608831
727.75	0.358973
728.23	0.113251
728.71	-0.211153
729.189	-0.370029
729.669	-0.555171
730.149	-0.711109
730.628	-0.844629
731.108	-0.548576
731.586	-0.479136
732.065	-0.496969
732.545	-0.508212
733.024	-0.568027
733.504	-0.596304
733.983	-0.852705
734.463	-1.16133
734.942	-1.70414
735.421	-2.10043
735.901	-2.51285
736.378	-2.7605
736.857	-2.99853
737.337	-3.13008
737.816	-2.8941
738.295	-2.54805
738.774	-1.93704
739.253	-1.25257
739.731	-0.49858
740.21	0.291133
740.689	1.10323
741.168	1.88192
741.647	2.5808
742.124	2.95197

742.603	3.32814
743.082	3.36958
743.561	3.16585
744.04	2.74454
744.517	1.8289
744.996	0.786925
745.474	-0.290893
745.953	-1.21899
746.43	-1.43457
746.909	-1.60363
747.388	-1.62543
747.866	-1.44342
748.343	-1.21119
748.822	-0.876925
749.3	-0.689575
749.779	-0.423404
750.255	0.213625
750.734	0.910224
751.213	1.55993
751.689	2.11127
752.168	2.53648
752.646	2.8493
753.123	2.80057
753.601	2.64605
754.079	2.44425
754.556	2.23467
755.034	2.01602
755.512	1.80758
755.989	1.68483
756.467	1.51616
756.945	1.44752
757.421	1.29333
757.9	1.33203
758.376	1.38249
758.854	1.5228
759.332	1.71569
759.808	1.88845
760.286	2.105
760.762	2.31514
761.24	2.51909
761.718	2.71674
762.194	2.91106
762.672	3.09613
763.148	3.26638
763.626	3.42859
764.102	3.55967
764.58	3.6434
765.055	3.6169
765.533	3.58414
766.011	3.50926
766.487	3.26857
766.964	3.13772
767.44	2.95791
767.918	2.75809
768.393	2.5494
768.871	2.2849
769.347	1.91191
769.824	1.47661
770.3	1.09521
770.775	0.762316
771.253	0.61967
771.728	1.50861

772.206	2.19216
772.681	2.85426
773.158	3.32259
773.634	3.86615
774.111	4.28317
774.586	4.72002
775.062	5.04576
775.539	5.29792
776.014	5.47141
776.491	5.43198
776.967	5.35302
777.442	5.11621
777.919	4.44956
778.394	3.71057
778.871	2.99726
779.346	2.29289
779.821	1.70167
780.298	1.76667
780.773	1.77474
781.248	1.90332
781.725	2.16369
782.2	2.56001
782.675	3.04632
783.152	3.62987
783.627	4.28169
784.101	5.16198
784.578	5.97892
785.053	6.37021
785.528	5.79137
786.004	4.92472
786.479	3.77427
786.954	2.45242
787.428	1.56598
787.905	1.29829
788.38	1.43714
788.854	1.74204
789.329	2.32944
789.805	2.91328
790.28	3.8266
790.754	5.1837
791.229	6.65894
791.705	7.46156
792.179	7.72593
792.654	7.59007
793.128	7.4452
793.602	7.27812
794.079	7.00332
794.553	5.46869
795.027	4.7472
795.501	4.21697
795.976	3.87041
796.452	3.67544
796.926	3.68115
797.4	3.7695
797.874	3.89496
798.348	3.96835
798.822	3.92017
799.296	4.3185
799.772	4.73772
800.246	4.87061
800.72	4.84806
801.194	4.76951

801.668	4.51102
802.142	4.22127
802.616	3.82564
803.089	3.28479
803.563	2.74098
804.037	2.30862
804.513	2.02012
804.986	2.03139
805.46	2.5374
805.934	3.22163
806.407	3.95144
806.881	4.704
807.355	5.40392
807.828	5.98291
808.302	6.50575
808.775	7.0544
809.249	7.32865
809.722	7.37589
810.196	7.4308
810.669	7.38364
811.142	7.3383
811.616	7.22876
812.089	7.17122
812.562	7.09289
813.036	7.03994
813.509	7.04671
813.982	7.29872
814.455	7.88476
814.929	8.46048
815.402	9.00949
815.875	9.61859
816.348	10.1946
816.819	10.7583
817.292	11.17
817.765	11.6223
818.238	11.8163
818.711	11.5169
819.184	11.1632
819.657	10.7475
820.13	10.3445
820.603	9.86067
821.076	9.40976
821.547	8.93875
822.02	8.51877
822.492	8.07748
822.965	7.86636
823.438	7.63192
823.911	7.49125
824.383	7.34798
824.854	7.15028
825.327	6.98962
825.799	6.81266
826.272	6.66516
826.744	6.52666
827.215	6.40042
827.688	6.30353
828.16	6.19913
828.633	6.12549
829.105	6.08872
829.575	6.06219
830.048	6.01628
830.52	6.01003

830.993	6.28868
831.463	6.5634
831.935	6.87201
832.407	7.38465
832.88	7.74278
833.35	8.08312
833.822	8.347
834.294	8.57878
834.765	8.74701
835.237	8.4451
835.709	8.06774
836.181	7.59724
836.651	7.08261
837.123	6.41447
837.595	5.68686
838.065	4.85766
838.537	3.98177
839.009	3.02079
839.479	1.93548
839.951	0.950839
840.422	0.566237
840.892	0.92175
841.364	1.3791
841.834	2.12023
842.306	2.80744
842.777	3.30647
843.247	3.81388
843.719	4.31593
844.189	4.76764
844.66	5.23006
845.132	5.66065
845.601	6.10643
846.073	6.56229
846.542	6.98227
847.014	7.3091
847.485	7.63197
847.955	7.88307
848.426	8.06937
848.896	8.20778
849.367	8.32401
849.837	8.39736
850.308	8.35813
850.777	8.30451
851.249	8.21947
851.718	8.04136
852.189	7.90487
852.658	7.66598
853.13	7.40036
853.599	7.13698
854.07	6.85468
854.539	6.5782
855.01	6.30324
855.479	6.04662
855.95	5.80647
856.419	5.62521
856.888	5.67964
857.359	5.76828
857.828	5.96796
858.299	6.19727
858.768	6.55687
859.239	6.92605
859.708	7.58204

860.177	8.43461
860.647	9.14697
861.116	9.63893
861.587	9.20971
862.056	8.9847
862.524	8.74802
862.995	8.51823
863.464	8.27954
863.932	8.08927
864.403	7.85987
864.872	7.65567
865.34	7.39826
865.811	7.32581
866.279	8.66333
866.748	8.81909
867.218	8.76955
867.687	8.67906
868.155	8.56012
868.625	8.41356
869.094	8.24813
869.562	8.14025
870.033	8.08122
870.501	8.16896
870.969	8.23843
871.437	8.26383
871.908	8.05997
872.376	7.32559
872.844	6.64971
873.312	6.04674
873.782	5.61154
874.25	5.38463
874.719	5.26234
875.187	5.34107
875.657	5.55654
876.125	5.8367
876.593	6.46987
877.061	7.12964
877.529	7.85649
877.996	8.59855
878.466	9.33786
878.934	10.0524
879.402	10.7169
879.87	11.3048
880.338	11.5528
880.805	11.5894
881.275	11.4931
881.743	11.222
882.211	10.9089
882.678	10.7318
883.146	10.4432
883.613	10.067
884.081	9.68887
884.549	9.28031
885.018	8.93184
885.486	8.65693
885.953	8.45885
886.421	8.39042
886.888	8.48577
887.356	8.79527
887.823	9.41074
888.29	10.0787
888.758	10.7591

889.225	11.099
889.692	11.378
890.16	11.2981
890.627	11.1384
891.094	10.9387
891.561	10.668
892.029	10.4497
892.496	10.1865
892.963	9.89652
893.43	9.76265
893.897	9.64946
894.364	9.7193
894.831	9.77989
895.298	9.81772
895.765	10.0652
896.232	10.0977
896.699	10.0618
897.166	10.0534
897.633	9.99061
898.1	9.96199
898.567	9.88484
899.033	9.83185
899.5	9.72977
899.967	9.63221
900.434	9.47401
900.9	9.38343
901.365	9.59262
901.832	9.83259
902.298	10.2987
902.765	10.6784
903.232	11.0181
903.698	11.3935
904.165	11.6873
904.631	11.9649
905.096	12.1594
905.562	12.293
906.029	11.963
906.495	11.5807
906.962	11.1485
907.428	10.3849
907.893	9.55311
908.359	8.69801
908.825	8.09444
909.291	7.56857
909.758	7.89845
910.222	7.98295
910.688	8.20425
911.154	8.33581
911.621	8.59354
912.085	8.95584
912.551	9.42812
913.017	10.1704
913.483	11.1691
913.947	11.4403
914.413	11.2572
914.879	10.5286
915.345	9.56831
915.809	8.64904
916.275	7.64515
916.741	5.62745
917.207	6.16632
917.671	7.07653

918.136	8.25631
918.602	10.2234
919.066	11.9446
919.532	13.4594
919.998	14.9236
920.461	15.0078
920.927	15.2052
921.393	15.3993
921.856	15.5699
922.322	15.5747
922.786	15.6696
923.251	15.6601
923.717	15.9426
924.18	16.322
924.646	16.791
925.111	17.3073
925.575	17.9372
926.04	18.8091
926.504	19.8138
926.969	21.3418
927.432	23.2177
927.898	26.1428
928.363	31.5099
928.826	34.9289
929.292	39.0552
929.755	43.1704
930.22	47.295
930.683	51.2915
931.148	55.3004
931.612	59.2083
932.077	63.1206
932.54	66.9088
933.005	70.6813
933.468	74.485
933.933	78.2978
934.396	82.1418
934.861	86.0578
935.324	89.9843
935.789	94.0578
936.252	98.1421
936.717	102.458
937.179	107.11
937.644	111.667
938.107	116.363
938.57	121.205
939.035	126.064
939.497	130.911
939.962	135.544
940.425	139.359
940.889	142.937
941.352	145.942
941.815	147.948
942.279	149.379
942.742	150.301
943.204	150.963
943.669	151.074
944.132	150.834
944.596	149.839
945.058	149.035
945.521	147.961
945.985	147.083
946.448	146.329

946.91	145.816
947.374	145.524
947.837	145.501
948.299	145.621
948.763	145.964
949.225	146.427
949.688	147.131
950.15	147.97
950.614	149.009
951.076	150.095
951.538	151.237
952	152.063
952.465	151.974
952.927	152.182
953.389	152.372
953.851	152.431
954.315	152.34
954.777	152.02
955.239	151.315
955.701	150.395
956.164	149.411
956.626	148.662
957.088	148.09
957.55	147.389
958.012	146.67
958.475	146.462
958.937	146.793
959.399	147.812
959.861	148.264
960.322	148.401
960.784	148.769
961.246	149.314
961.709	149.731
962.171	150.056
962.632	150.194
963.094	150.324
963.555	150.276
964.017	150.106
964.478	149.885
964.94	149.553
965.403	149.033
965.865	148.479
966.326	147.857
966.787	147.13
967.249	146.318
967.71	145.503
968.171	144.459
968.633	143.429
969.094	142.397
969.555	141.362
970.016	140.326
970.477	139.345
970.938	138.331
971.4	137.707
971.861	137.202
972.322	136.942
972.783	136.884
973.244	137.004
973.705	137.102
974.166	137.146
974.627	137.109
975.088	137.054

975.548	136.727
976.009	136.256
976.47	135.751
976.931	135.406
977.392	135.088
977.853	134.671
978.313	133.967
978.774	132.905
979.235	131.674
979.695	130.112
980.154	128.73
980.615	128.097
981.076	127.475
981.536	127.009
981.997	126.687
982.457	126.365
982.918	126.079
983.378	125.697
983.837	125.085
984.297	124.254
984.758	123.095
985.218	121.599
985.678	119.745
986.139	117.472
986.597	114.857
987.057	111.89
987.518	108.688
987.978	105.49
988.438	102.322
988.896	98.9526
989.357	95.5201
989.817	92.0183
990.277	88.379
990.737	84.7449
991.195	80.9991
991.655	77.2608
992.115	73.5543
992.575	69.9633
993.033	66.6249
993.493	63.0128
993.953	59.4835
994.413	56.0848
994.871	52.8232
995.331	49.6422
995.791	46.5673
996.249	43.6394
996.709	40.9597
997.168	38.4947
997.626	36.5276
998.086	34.5538
998.546	32.6509
999.003	30.9317
999.463	29.5947
999.923	28.4979
1000.38	27.4463
1000.84	26.68
1001.3	26.0905
1001.76	25.5146
1002.22	24.8759
1002.67	24.144
1003.13	23.3772
1003.59	22.5774

1004.05	21.6814
1004.51	20.6789
1004.97	19.3822
1005.43	17.8121
1005.89	16.1876
1006.06	15.487
1006.53	14.6739
1006.99	13.9974
1007.45	13.466
1007.92	13.1003
1008.38	12.87
1008.84	12.7786
1009.31	12.8022
1009.77	12.6655
1010.24	12.6448
1010.7	12.4051
1011.16	11.7156
1011.63	11.0895
1012.09	10.8102
1012.55	10.7796
1013.02	10.8326
1013.48	10.9337
1013.94	10.7291
1014.4	10.5823
1014.87	10.692
1015.33	11.0216
1015.79	11.7747
1016.26	11.9977
1016.72	12.1799
1017.18	12.2861
1017.65	12.4271
1018.11	12.2684
1018.57	12.0369
1019.03	11.794
1019.5	11.5324
1019.96	11.265
1020.42	10.9969
1020.89	10.7409
1021.35	10.4863
1021.81	10.2599
1022.28	10.0375
1022.74	9.81536
1023.2	9.49714
1023.66	9.50363
1024.12	9.5236
1024.59	9.63519
1025.05	9.77591
1025.51	9.95085
1025.97	10.1486
1026.44	10.3529
1026.9	10.5437
1027.36	10.7448
1027.82	10.9658
1028.29	11.3161
1028.75	11.3424
1029.21	11.3339
1029.67	11.0479
1030.13	10.8161
1030.6	10.5812
1031.06	10.3214
1031.52	10.0518
1031.98	9.84093

1032.44	9.7505
1032.9	9.71018
1033.37	9.69051
1033.83	10.0833
1034.29	10.3059
1034.75	10.5113
1035.21	10.7029
1035.67	10.8621
1036.14	10.9891
1036.6	11.0804
1037.06	11.1229
1037.52	11.1218
1037.98	10.7531
1038.44	10.4512
1038.91	10.034
1039.37	9.42856
1039.83	8.86177
1040.29	8.34731
1040.75	8.38017
1041.21	9.67184
1041.67	8.93956
1042.13	8.03226
1042.59	6.87492
1043.06	5.44605
1043.52	4.28095
1043.98	3.27175
1044.44	2.03653
1044.9	2.36523
1045.36	2.78653
1045.82	3.14776
1046.28	3.51592
1046.74	3.95079
1047.2	4.21944
1047.67	4.36455
1048.13	4.5108
1048.59	4.56856
1049.05	4.63618
1049.51	4.64809
1049.97	4.65729
1050.43	4.64035
1050.89	4.60838
1051.35	4.57291
1051.81	4.51333
1052.27	4.46529
1052.73	4.3885
1053.19	4.33161
1053.65	4.24521
1054.11	4.18038
1054.57	4.08718
1055.03	4.01381
1055.49	3.91504
1055.95	3.83381
1056.41	3.72986
1056.87	3.6686
1057.33	3.56824
1057.79	3.49917
1058.25	3.40843
1058.71	3.30738
1059.17	3.21295
1059.63	3.11044
1060.09	3.01552
1060.55	2.91526

1061.01	2.82641
1061.47	2.73811
1061.93	2.65768
1062.39	2.48953
1062.85	1.42212
1063.31	0.743932
1063.77	0.138859
1064.23	-0.40054
1064.69	-0.279114
1065.15	-0.0532241
1065.61	0.233217
1066.07	0.623434
1066.53	1.05275
1066.98	1.45316
1067.44	1.75284
1067.9	1.92068
1068.36	2.13313
1068.82	2.23713
1069.28	2.37743
1069.74	2.45347
1070.2	2.53199
1070.66	2.6166
1071.12	2.69401
1071.57	2.76253
1072.03	2.84538
1072.49	2.89974
1072.95	2.98684
1073.41	3.06096
1073.87	3.1609
1074.33	3.24385
1074.79	3.32663
1075.25	3.54609
1075.7	3.74148
1076.16	3.93877
1076.62	4.1275
1077.08	4.29664
1077.54	4.46008
1078	4.59628
1078.45	4.7144
1078.91	4.81292
1079.37	4.8965
1079.83	4.95092
1080.29	4.99154
1080.75	4.99739
1081.2	4.9926
1081.66	4.94681
1082.12	4.89403
1082.58	4.81442
1083.04	4.71607
1083.5	4.58404
1083.95	4.42249
1084.41	4.27968
1084.87	3.97759
1085.33	3.67781
1085.78	3.36351
1086.24	3.0372
1086.7	2.71448
1087.16	2.41227
1087.62	2.11072
1088.07	1.83506
1088.53	1.60136
1088.99	1.24942

1089.45	1.51252
1089.9	1.92664
1090.36	2.32691
1090.82	2.75597
1091.28	3.2077
1091.73	3.66676
1092.19	4.11027
1092.65	4.54756
1093.11	4.95805
1093.56	5.36278
1094.02	5.60157
1094.48	5.77339
1094.94	5.9623
1095.39	6.12098
1095.85	6.26941
1096.31	6.39573
1096.76	6.52022
1097.22	6.59172
1097.68	6.61943
1098.13	6.59485
1098.59	6.55014
1099.05	6.46649
1099.5	6.18046
1099.96	5.91113
1100.42	5.57454
1100.88	5.25306
1101.33	4.88902
1101.79	4.53213
1102.24	4.16143
1102.7	3.78623
1103.16	3.43333
1103.62	3.07304
1104.07	2.75199
1104.53	2.45258
1104.98	2.63391
1105.44	2.57335
1105.9	2.77018
1106.35	3.00534
1106.81	3.29312
1107.27	3.61811
1107.72	3.94102
1108.18	4.30642
1108.63	4.8276
1109.09	4.99786
1109.55	5.15435
1110	5.30209
1110.46	5.08515
1110.92	5.01261
1111.37	4.88816
1111.83	4.77636
1112.28	4.64734
1112.74	4.53005
1113.19	4.40417
1113.65	4.26816
1114.11	4.15682
1114.56	3.99445
1115.02	3.97613
1115.47	3.87052
1115.93	3.77121
1116.38	3.65433
1116.84	3.52087
1117.29	3.40899

1117.75	3.28362
1118.21	2.96044
1118.66	2.6777
1119.12	2.33805
1119.57	2.03748
1120.03	1.70603
1120.48	1.40634
1120.94	1.11269
1121.39	0.841754
1121.85	0.588506
1122.3	0.406349
1122.76	0.249438
1123.21	0.153012
1123.67	0.585921
1124.12	0.873353
1124.58	1.22037
1125.03	1.68289
1125.49	2.15781
1125.94	2.77005
1126.4	3.4343
1126.85	4.10694
1127.31	4.80385
1127.76	5.45928
1128.22	5.65798
1128.67	5.12072
1129.13	4.28065
1129.58	3.06673
1130.04	2.00971
1130.49	1.08992
1130.94	0.249834
1131.4	-0.533857
1131.85	-0.760366
1132.31	2.17971
1132.76	2.3765
1133.22	2.59045
1133.67	1.96669
1134.13	1.87216
1134.58	1.97984
1135.03	2.2809
1135.49	2.61357
1135.94	2.91231
1136.4	2.98411
1136.85	2.76909
1137.3	2.32823
1137.76	2.20589
1138.21	2.32627
1138.67	2.51841
1139.12	2.72813
1139.57	2.90861
1140.03	2.86526
1140.48	2.72831
1140.93	2.54377
1141.39	2.3651
1141.84	2.21296
1142.3	2.10822
1142.75	2.05527
1143.2	2.11942
1143.66	2.35539
1144.11	2.79365
1144.56	3.08921
1145.02	3.13927
1145.47	2.78777

1145.92	2.32311
1146.38	1.77256
1146.83	1.16137
1147.28	0.850667
1147.74	0.611939
1148.19	0.406109
1148.64	0.0954442
1149.1	-0.230549
1149.55	-0.534889
1150	-0.532728
1150.46	-0.458918
1150.91	-0.339613
1151.36	-0.202717
1151.82	-0.0222096
1152.27	0.218728
1152.72	0.588986
1153.17	0.989645
1153.63	1.33691
1154.08	1.40902
1154.53	1.29175
1154.98	0.999671
1155.44	1.36459
1155.89	1.68104
1156.34	1.88469
1156.8	1.94581
1157.25	1.86978
1157.7	1.31664
1158.15	1.0074
1158.6	0.91621
1159.06	0.887082
1159.51	0.895689
1159.96	0.93498
1160.41	1.19438
1160.87	1.61526
1161.32	2.05328
1161.77	2.4269
1162.22	2.62452
1162.67	2.76381
1163.13	2.91566
1163.58	3.27934
1164.03	3.5125
1164.48	3.60654
1164.93	3.52913
1165.39	3.24731
1165.84	2.77329
1166.29	2.11547
1166.74	1.26163
1167.19	0.645784
1167.65	0.109262
1168.1	-0.461495
1168.55	-0.968646
1169	-0.90269
1169.45	-0.753995
1169.9	-0.713905
1170.36	-0.613786
1170.81	-0.437152
1171.26	-0.145749
1171.71	0.281579
1172.16	0.889929
1172.61	1.52179
1173.06	2.09534
1173.52	2.59772

1173.97	3.03394
1174.42	3.40939
1174.87	3.69083
1175.32	3.91975
1175.77	4.04884
1176.22	4.1302
1176.67	4.1078
1177.12	4.03713
1177.58	3.86517
1178.03	3.64427
1178.48	3.33154
1178.93	2.97052
1179.38	2.53682
1179.83	2.06646
1180.28	1.55585
1180.73	1.05155
1181.18	0.544645
1181.63	0.386811
1182.08	1.25428
1182.53	1.7576
1182.98	1.97283
1183.44	2.20372
1183.89	2.51681
1184.34	2.89218
1184.79	3.23152
1185.24	3.42434
1185.69	3.5524
1186.14	3.60095
1186.59	3.58767
1187.04	3.53421
1187.49	3.46512
1187.94	3.37113
1188.39	3.2002
1188.84	2.95912
1189.29	2.7589
1189.74	2.65374
1190.19	2.58749
1190.64	2.47856
1191.09	2.27551
1191.54	1.96722
1191.99	1.53207
1192.44	0.983651
1192.89	0.248422
1193.34	-0.474471
1193.79	-0.690452
1194.24	-0.632354
1194.69	-0.26042
1195.14	0.37761
1195.59	0.368081
1196.04	0.501312
1196.49	0.727478
1196.94	0.964819
1197.39	1.16268
1197.84	1.30515
1198.29	1.38773
1198.74	1.40248
1199.18	1.37467
1199.63	1.14323
1200.08	0.928518
1200.53	0.77229
1200.98	0.70701
1201.43	0.766816

1201.88	0.902013
1202.33	1.11216
1202.78	1.38416
1203.23	1.67475
1203.68	2.23464
1204.12	2.84036
1204.57	3.44378
1205.02	4.01556
1205.47	4.53018
1205.92	4.98259
1206.37	5.37981
1206.82	5.66196
1207.27	5.60937
1207.72	5.28336
1208.16	4.69461
1208.61	4.07782
1209.06	3.43962
1209.51	2.81488
1209.96	2.20169
1210.41	1.61423
1210.86	1.09349
1211.3	0.681174
1211.75	0.528086
1212.2	0.502043
1212.65	0.499444
1213.1	0.862614
1213.55	1.33834
1213.99	1.99579
1214.44	2.53418
1214.89	3.17325
1215.34	3.81576
1215.79	4.37601
1216.24	4.87172
1216.68	5.35789
1217.13	5.47319
1217.58	5.74952
1218.03	5.92689
1218.48	6.06464
1218.92	6.17399
1219.37	6.26891
1219.82	6.34157
1220.27	6.41254
1220.72	6.45955
1221.16	6.49632
1221.61	6.5303
1222.06	6.64835
1222.51	6.83671
1222.95	6.68601
1223.4	6.53169
1223.85	6.36759
1224.3	6.22293
1224.74	6.08672
1225.19	5.97972
1225.64	5.90034
1226.09	5.83966
1226.53	5.80013
1226.98	5.81168
1227.43	5.89407
1227.87	5.91816
1228.32	6.28577
1228.77	6.67026
1229.22	7.11467

1229.66	7.53997
1230.11	7.9554
1230.56	8.37178
1231	8.76566
1231.45	9.11189
1231.9	9.45851
1232.34	9.62261
1232.79	9.48299
1233.24	9.27208
1233.69	9.03809
1234.13	8.76376
1234.58	8.49058
1235.03	8.22589
1235.47	7.9553
1235.92	7.70946
1236.37	7.48104
1236.81	7.25906
1237.26	7.1454
1237.71	7.14452
1238.15	7.25992
1238.6	7.41259
1239.04	7.65125
1239.49	7.97347
1239.94	8.24322
1240.38	8.8132
1240.83	9.35304
1241.28	9.93981
1241.72	10.5223
1242.17	11.0555
1242.61	11.531
1243.06	11.8767
1243.51	12.2055
1243.95	12.0961
1244.4	11.8546
1244.84	11.5526
1245.29	11.0523
1245.74	10.4329
1246.18	9.67699
1246.63	8.83932
1247.07	7.92709
1247.52	6.94416
1247.96	5.892
1248.41	4.83068
1248.86	3.74221
1249.3	3.33358
1249.75	3.8101
1250.19	4.4431
1250.64	5.3972
1251.08	6.58397
1251.53	7.79283
1251.98	9.38042
1252.42	10.7848
1252.87	11.8861
1253.31	12.6803
1253.76	13.1448
1254.2	13.3014
1254.65	13.1377
1255.09	12.5991
1255.54	11.3266
1255.98	10.9922
1256.43	10.7019
1256.87	10.4923

1257.32	10.351
1257.76	10.2891
1258.21	10.2463
1258.65	9.98674
1259.1	9.68426
1259.54	9.41856
1259.99	9.27144
1260.43	9.32153
1260.88	9.62815
1261.32	9.78671
1261.77	9.51041
1262.21	9.15736
1262.65	8.8875
1263.1	9.15248
1263.55	9.51207
1263.99	9.784
1264.43	10.1622
1264.88	10.4525
1265.32	10.5206
1265.77	10.4139
1266.21	10.1514
1266.66	9.84591
1267.1	9.55189
1267.54	9.27186
1267.99	9.01021
1268.43	8.77404
1268.88	8.56681
1269.32	8.38774
1269.76	8.24349
1270.21	8.10752
1270.65	7.99441
1271.1	8.3001
1271.54	8.6666
1271.99	9.07984
1272.43	9.51719
1272.87	9.97606
1273.32	10.4311
1273.76	10.8706
1274.2	11.2707
1274.65	11.6007
1275.09	11.8794
1275.53	11.6148
1275.98	11.5926
1276.42	11.2761
1276.87	10.8834
1277.31	10.4306
1277.75	9.96046
1278.2	9.47335
1278.64	8.98602
1279.08	8.5484
1279.53	8.14755
1279.97	8.2585
1280.41	8.40003
1280.86	8.62178
1281.3	8.87719
1281.74	9.17756
1282.18	9.50373
1282.63	9.50914
1283.07	9.55069
1283.51	9.28888
1283.96	9.04665
1284.4	8.79074

1284.84	8.53134
1285.29	8.31353
1285.73	8.1414
1286.17	8.10177
1286.61	8.06416
1287.06	8.27462
1287.5	8.39363
1287.94	8.81204
1288.39	9.24671
1288.83	9.71852
1289.27	10.1802
1289.71	10.6003
1290.16	10.934
1290.6	10.7868
1291.04	10.7012
1291.48	10.4739
1291.93	10.0872
1292.37	10.1436
1292.81	10.3451
1293.25	10.6191
1293.7	10.7688
1294.14	11.3569
1294.58	11.5581
1295.02	11.7357
1295.46	11.7695
1295.91	11.3317
1296.35	10.9955
1296.79	10.8503
1297.23	10.891
1297.67	11.1486
1298.12	11.5529
1298.56	11.8938
1299	11.8042
1299.44	11.5942
1299.88	10.9988
1300.33	10.8553
1300.77	10.9523
1301.21	11.2988
1301.65	11.7584
1302.09	12.4739
1302.53	13.0462
1302.98	13.3339
1303.42	13.1427
1303.86	13.3743
1304.3	13.1101
1304.74	12.5181
1305.18	12.2164
1305.63	12.1087
1306.07	12.1576
1306.51	12.3162
1306.95	12.5559
1307.39	12.6912
1307.83	12.7485
1308.27	13.3089
1308.71	13.8845
1309.15	14.3904
1309.6	14.8351
1310.04	15.1707
1310.48	15.3494
1310.92	15.3029
1311.36	15.0021
1311.8	14.4012

1312.24	13.5371
1312.68	13.2141
1313.12	12.9853
1313.56	12.685
1314	13.3039
1314.45	13.9156
1314.89	14.4982
1315.33	15.0764
1315.77	15.6573
1316.21	16.2398
1316.65	16.821
1317.09	17.4495
1317.53	17.6413
1317.97	17.6928
1318.41	17.6744
1318.85	17.5918
1319.29	17.4701
1319.73	17.2886
1320.17	17.0694
1320.61	16.7871
1321.05	16.445
1321.49	15.9984
1321.93	15.1767
1322.37	13.9245
1322.81	12.841
1323.25	11.8939
1323.69	11.0145
1324.13	10.6875
1324.57	11.0315
1325.01	11.2409
1325.45	11.6783
1325.89	12.7288
1326.33	13.0318
1326.77	13.2586
1327.21	13.7504
1327.65	14.4317
1328.09	15.0602
1328.53	15.4707
1328.97	15.6849
1329.41	15.7196
1329.85	15.4694
1330.29	15.0922
1330.73	14.9946
1331.17	14.9519
1331.61	14.9054
1332.05	14.8772
1332.49	14.3726
1332.93	13.7719
1333.36	13.1498
1333.8	12.2016
1334.24	11.6321
1334.68	11.2347
1335.12	10.6753
1335.56	10.2343
1336	10.0916
1336.44	8.9265
1336.88	8.98948
1337.32	9.34138
1337.76	10.2656
1338.2	11.2705
1338.63	12.3056
1339.07	13.3676

1339.51	14.6556
1339.95	15.671
1340.39	16.5849
1340.83	17.2257
1341.27	17.4878
1341.71	17.4309
1342.14	17.0699
1342.58	16.509
1343.02	16.151
1343.46	15.744
1343.9	15.3447
1344.34	14.844
1344.78	14.6854
1345.21	14.392
1345.65	14.3225
1346.09	14.2364
1346.53	14.1364
1346.97	13.9936
1347.41	13.6831
1347.84	13.3332
1348.28	12.9795
1348.72	12.5981
1349.16	12.3854
1349.6	12.2958
1350.03	12.426
1350.47	12.2841
1350.91	12.314
1351.35	12.5392
1351.79	13.0412
1352.22	13.0569
1352.66	12.9394
1353.1	12.7278
1353.54	12.4239
1353.97	12.0027
1354.41	11.4901
1354.85	10.8905
1355.29	10.2572
1355.73	9.96408
1356.16	9.13503
1356.6	8.58328
1357.04	8.39187
1357.47	8.36249
1357.91	8.40297
1358.35	8.50175
1358.79	8.65287
1359.22	8.81764
1359.66	8.99073
1360.1	9.04123
1360.54	9.28984
1360.97	9.16435
1361.41	8.61749
1361.85	7.88641
1362.29	7.9068
1362.72	8.02657
1363.16	7.71633
1363.6	7.52033
1364.03	7.49547
1364.47	7.61352
1364.91	7.88198
1365.34	8.22224
1365.78	8.4702
1366.22	8.72458

1366.65	8.96662
1367.09	9.31242
1367.53	10.0656
1367.97	10.6023
1368.4	11.0954
1368.84	11.5913
1369.27	12.2899
1369.71	12.5665
1370.15	12.8347
1370.58	13.1415
1371.02	13.481
1371.46	13.8862
1371.89	14.2782
1372.33	14.8252
1372.77	15.5255
1373.2	16.2496
1373.64	16.7842
1374.08	17.2932
1374.51	17.7391
1374.95	18.0639
1375.38	18.3001
1375.82	18.4362
1376.26	18.495
1376.69	18.5131
1377.13	18.7023
1377.56	19.1148
1378	19.6049
1378.43	19.6395
1378.87	19.6276
1379.31	19.3447
1379.74	18.894
1380.18	18.2329
1380.61	17.3962
1381.05	16.5977
1381.49	16.039
1381.92	15.6872
1382.36	15.9616
1382.79	16.4881
1383.23	17.2066
1383.66	18.1132
1384.1	19.242
1384.53	20.6883
1384.97	22.0893
1385.41	23.0192
1385.84	23.7083
1386.28	24.1593
1386.71	24.3993
1387.15	24.4639
1387.58	24.3568
1388.02	24.243
1388.45	24.1562
1388.89	23.718
1389.32	22.9131
1389.76	22.5039
1390.19	22.0961
1390.63	21.7103
1391.06	21.3626
1391.5	20.9248
1391.93	20.7221
1392.37	20.1944
1392.8	19.637
1393.24	19.47

1393.67	18.168
1394.11	15.8811
1394.54	18.3833
1394.98	21.1133
1395.41	19.3758
1395.84	17.8742
1396.28	17.5948
1396.71	16.9523
1397.15	16.7074
1397.58	16.2141
1398.02	15.7228
1398.45	14.9235
1398.89	13.3395
1399.32	13.0572
1399.75	13.4334
1400.19	13.4433
1400.62	13.3917
1401.06	13.3231
1401.49	13.1878
1401.92	13.0371
1402.36	12.8853
1402.79	12.8169
1403.23	12.6188
1403.66	12.3641
1404.1	12.0517
1404.53	11.6825
1404.96	11.4124
1405.4	10.9877
1405.83	10.5792
1406.26	10.1865
1406.7	9.74106
1407.13	9.09111
1407.57	8.66397
1408	8.20975
1408.43	7.53295
1408.87	6.63041
1409.3	5.50516
1409.73	4.2713
1410.17	3.14497
1410.6	2.31006
1411.03	1.84984
1411.47	1.7884
1411.9	2.06858
1412.33	2.69966
1412.77	4.52939
1413.2	6.42472
1413.63	6.21639
1414.07	3.77366
1414.5	1.15496
1414.93	0.267868
1415.37	-0.601321
1415.8	-1.44063
1416.23	-2.02522
1416.67	-2.54599
1417.1	-3.05298
1417.53	-3.54439
1417.96	-4.04159
1418.26	-4.13557
1418.7	-3.86096
1419.14	-3.578
1419.57	-3.27326
1420.01	-2.94224

1420.45	-2.61947
1420.89	-2.30688
1421.33	-1.98409
1421.76	-1.68921
1422.2	-1.3775
1422.64	-1.09548
1423.08	-0.832865
1423.51	-0.559304
1423.95	-0.362885
1424.39	-0.135612
1424.83	0.0580919
1425.26	0.249768
1425.7	0.415426
1426.14	0.567604
1426.58	0.68369
1427.01	0.793016
1427.45	0.923916
1427.89	1.03742
1428.33	1.14336
1428.77	1.23474
1429.2	1.3117
1429.64	1.36724
1430.08	1.41316
1430.51	1.4506
1430.95	1.45266
1431.39	1.4413
1431.82	1.40421
1432.26	1.32981
1432.7	1.16903
1433.14	0.955238
1433.57	0.758576
1434.01	0.562366
1434.45	0.765624
1434.88	1.27256
1435.32	1.94059
1435.76	2.74716
1436.19	3.64954
1436.63	4.61829
1437.07	5.52072
1437.5	6.53481
1437.94	7.47176
1438.38	8.2913
1438.81	8.87473
1439.25	9.22813
1439.69	9.38571
1440.12	8.78771
1440.56	8.13127
1441	7.38497
1441.43	6.66687
1441.87	6.08278
1442.31	5.575
1442.74	4.98941
1443.18	4.92549
1443.61	5.03295
1444.05	5.157
1444.49	5.39218
1444.92	5.62097
1445.36	5.88176
1445.79	6.17309
1446.23	6.44106
1446.67	6.765
1447.1	6.8793

1447.54	6.97643
1447.97	7.13458
1448.41	7.34249
1448.85	7.39052
1449.28	7.30079
1449.72	7.26265
1450.15	7.24366
1450.59	7.28663
1451.02	7.30547
1451.46	7.30456
1451.9	7.31023
1452.33	7.36239
1452.77	7.43519
1453.2	7.47215
1453.64	7.33432
1454.07	7.39289
1454.51	7.47688
1454.94	7.58504
1455.38	7.66714
1455.81	7.80447
1456.25	7.90285
1456.69	7.98318
1457.12	8.13653
1457.56	7.93992
1457.99	7.75615
1458.43	7.48682
1458.86	7.04882
1459.3	6.64664
1459.73	6.10088
1460.17	5.68973
1460.6	5.32763
1461.04	5.04042
1461.47	4.7103
1461.91	4.65957
1462.34	4.82925
1462.78	4.97618
1463.21	5.12484
1463.65	5.30893
1464.08	5.46166
1464.52	5.65102
1464.95	5.74261
1465.38	5.77574
1465.82	5.83915
1466.25	5.53795
1466.69	5.33577
1467.12	5.06333
1467.56	4.77828
1467.99	4.44791
1468.43	4.09461
1468.86	3.7396
1469.29	3.3295
1469.73	2.98702
1470.16	2.65389
1470.6	2.38013
1471.03	2.08841
1471.47	1.8725
1471.9	1.72143
1472.33	1.56537
1472.77	1.51045
1473.2	1.46904
1473.64	1.49107
1474.07	1.49714

1474.5	1.59096
1474.94	1.70507
1475.37	1.84354
1475.81	2.02494
1476.24	2.20408
1476.67	2.4142
1477.11	2.65465
1477.54	2.85253
1477.97	3.16858
1478.41	3.43715
1478.84	3.73831
1479.28	3.99611
1479.71	4.26666
1480.14	4.50274
1480.58	4.71992
1481.01	4.89904
1481.44	5.03675
1481.88	5.13427
1482.31	5.10035
1482.74	4.80996
1483.18	4.36619
1483.61	3.7766
1484.04	3.05881
1484.48	2.38405
1484.91	1.6967
1485.34	1.05479
1485.77	0.520749
1486.21	0.0656023
1486.64	0.481895
1487.07	0.842341
1487.51	1.34423
1487.94	2.28314
1488.37	3.32239
1488.81	4.37792
1489.24	5.09274
1489.67	5.59677
1490.1	6.00145
1490.54	6.22746
1490.97	6.17479
1491.4	5.79018
1491.84	5.16732
1492.27	4.5574
1492.7	4.20497
1493.13	4.29692
1493.57	4.43246
1494	4.07454
1494.43	3.80462
1494.86	3.84696
1495.3	4.15808
1495.73	4.69905
1496.16	5.30141
1496.59	5.86055
1497.02	6.40961
1497.46	6.90834
1497.89	7.33166
1498.32	7.61271
1498.75	7.83122
1499.19	7.93958
1499.62	7.88059
1500.05	7.75844
1500.48	7.74367
1500.91	8.14661

1501.35	8.12007
1501.78	7.91379
1502.21	7.04113
1502.64	6.83371
1503.07	6.87539
1503.51	7.09472
1503.94	7.36104
1504.37	7.69738
1504.8	8.07161
1505.23	8.41149
1505.66	8.77184
1506.1	9.18505
1506.53	9.35574
1506.96	8.90412
1507.39	8.23602
1507.82	7.43601
1508.25	5.73295
1508.68	5.15025
1509.12	4.50026
1509.55	4.00668
1509.98	3.635
1510.41	3.58025
1510.84	3.24468
1511.27	3.05784
1511.7	2.97683
1512.14	3.09726
1512.57	3.45998
1513	3.94473
1513.43	4.54656
1513.86	5.24473
1514.29	5.94617
1514.72	6.5653
1515.15	6.51327
1515.58	6.26293
1516.01	5.77495
1516.45	5.17472
1516.88	4.66784
1517.31	4.25845
1517.74	4.05779
1518.17	4.00669
1518.6	4.18259
1519.03	4.63081
1519.46	5.18955
1519.89	5.92961
1520.32	6.45437
1520.75	6.84961
1521.18	7.07867
1521.61	7.21668
1522.04	7.11615
1522.47	6.78556
1522.9	6.4392
1523.33	6.03301
1523.77	5.62825
1524.19	5.20142
1524.63	4.69734
1525.06	4.3798
1525.49	4.27471
1525.92	4.46789
1526.35	4.75727
1526.78	5.08946
1527.21	5.45109
1527.64	5.8036

1528.07	6.16782
1528.5	6.50976
1528.93	6.84028
1529.36	7.00562
1529.79	7.19817
1530.22	7.33269
1530.65	7.4776
1531.08	7.54221
1531.5	7.52787
1531.93	7.45395
1532.36	7.2882
1532.79	7.13745
1533.22	6.92162
1533.65	6.74084
1534.08	6.36061
1534.51	5.96825
1534.94	5.50025
1535.37	5.02538
1535.8	4.59678
1536.23	4.17948
1536.66	3.8399
1537.09	3.56017
1537.52	3.75788
1537.95	4.00234
1538.38	4.76442
1538.81	5.52463
1539.23	6.27741
1539.66	6.96976
1540.09	7.57537
1540.52	8.15706
1540.95	8.07334
1541.38	8.06021
1541.81	7.99329
1542.24	7.90375
1542.67	7.77629
1543.1	7.62624
1543.53	7.57429
1543.95	7.4611
1544.38	7.29883
1544.81	7.46222
1545.24	7.83917
1545.67	8.20593
1546.1	8.59152
1546.53	9.00108
1546.96	9.37399
1547.38	9.67414
1547.81	9.87929
1548.24	10.0142
1548.67	10.1063
1549.1	9.62108
1549.53	9.072
1549.95	8.52644
1550.38	8.01971
1550.81	7.46657
1551.24	6.93466
1551.67	6.39057
1552.1	5.87341
1552.52	5.4273
1552.95	5.33095
1553.38	5.25609
1553.81	5.24677
1554.24	5.31034

1554.66	5.38918
1555.09	5.50971
1555.52	5.63393
1555.95	5.7958
1556.38	5.96934
1556.8	6.15422
1557.23	6.39039
1557.66	6.63074
1558.09	6.90138
1558.52	7.18341
1558.94	7.47954
1559.37	7.77192
1559.8	8.08481
1560.23	8.34366
1560.65	8.46398
1561.08	8.74198
1561.51	9.00858
1561.94	9.30577
1562.36	9.57491
1562.79	9.84763
1563.22	10.125
1563.64	10.3774
1564.07	10.6344
1564.5	10.8697
1564.93	11.084
1565.35	11.3103
1565.78	11.4974
1566.21	11.7068
1566.64	11.8828
1567.06	12.0477
1567.49	12.1298
1567.92	12.2458
1568.34	12.3452
1568.77	12.3083
1569.2	12.254
1569.63	12.0506
1570.05	11.7722
1570.48	11.6239
1570.91	11.4834
1571.33	11.341
1571.76	11.2183
1572.19	11.125
1572.61	10.9916
1573.04	11.0771
1573.47	11.1521
1573.89	11.5064
1574.32	11.9763
1574.75	12.3638
1575.17	12.8988
1575.6	13.1438
1576.03	13.4034
1576.45	13.591
1576.88	13.7959
1577.31	13.8526
1577.73	13.7369
1578.16	13.6994
1578.58	13.607
1579.01	13.4723
1579.44	13.4738
1579.86	13.4468
1580.29	13.4444
1580.71	13.4696

1581.14	13.4694
1581.57	13.6668
1581.99	13.7372
1582.42	13.8203
1582.84	13.9245
1583.27	14.0877
1583.7	14.0793
1584.12	14.0631
1584.55	14.0444
1584.97	14.0104
1585.4	13.964
1585.83	13.7062
1586.25	13.5082
1586.68	13.5599
1587.1	13.6194
1587.53	13.706
1587.95	13.8239
1588.38	13.9248
1588.8	14.253
1589.23	14.5103
1589.65	14.7099
1590.08	14.9116
1590.51	14.9682
1590.93	14.9737
1591.36	14.9202
1591.78	14.8668
1592.21	14.7315
1592.63	14.411
1593.06	14.1003
1593.48	13.746
1593.91	13.6126
1594.33	13.4441
1594.76	13.2967
1595.18	13.1686
1595.61	13.0314
1596.03	13.0874
1596.46	13.0505
1596.88	12.9496
1597.31	12.8896
1597.73	12.4356
1598.16	11.9153
1598.58	11.1154
1599.01	10.4699
1599.43	9.77831
1599.86	9.12952
1600.28	8.46005
1600.71	8.01766
1601.13	8.00056
1601.56	8.28968
1601.98	8.67347
1602.41	9.06343
1602.83	9.58144
1603.25	9.92674
1603.68	10.319
1604.1	10.6848
1604.53	11.0484
1604.95	11.4231
1605.38	11.7716
1605.8	12.1519
1606.22	12.3271
1606.65	12.582
1607.07	12.9138

1607.5	13.245
1607.92	13.677
1608.34	14.1069
1608.77	14.5452
1609.19	15.2802
1609.62	16.2044
1610.04	17.0703
1610.46	17.6751
1610.89	18.2571
1611.31	18.7254
1611.74	18.5947
1612.16	18.1096
1612.58	17.6234
1613.01	17.0453
1613.43	16.4359
1613.85	15.6036
1614.28	14.7866
1614.7	13.9624
1615.13	13.2849
1615.55	12.647
1615.97	12.0398
1616.4	11.7303
1616.82	11.3276
1617.24	11.3171
1617.67	11.5688
1618.09	12.107
1618.51	12.7229
1618.94	13.3733
1619.36	14.2235
1619.78	15.0657
1620.21	15.9592
1620.63	16.7946
1621.05	17.476
1621.48	18.1312
1621.9	18.7479
1622.32	19.2484
1622.74	19.6184
1623.17	19.7549
1623.59	19.8551
1624.01	19.4324
1624.44	18.8788
1624.86	18.2403
1625.28	17.5685
1625.7	16.8923
1626.13	16.2347
1626.55	15.5711
1626.97	15.25
1627.39	14.916
1627.82	14.673
1628.24	14.5108
1628.66	14.2919
1629.09	14.4722
1629.51	14.6664
1629.93	14.9193
1630.35	15.1757
1630.77	15.459
1631.2	15.7324
1631.62	15.9573
1632.04	16.1873
1632.46	16.3187
1632.89	16.1188
1633.31	15.9112

1633.73	15.6458
1634.15	15.3338
1634.58	15.0146
1635	14.662
1635.42	14.2517
1635.84	13.8447
1636.26	13.3766
1636.69	12.9002
1637.11	12.3996
1637.53	11.9024
1637.95	11.3859
1638.37	11.0098
1638.79	10.5833
1639.22	10.4381
1639.64	9.97717
1640.06	9.59204
1640.48	9.17512
1640.9	8.79043
1641.33	8.43686
1641.75	8.11571
1642.17	7.82416
1642.59	7.64003
1643.01	7.3615
1643.43	7.13774
1643.85	6.98286
1644.28	7.01275
1644.7	7.1524
1645.12	7.32847
1645.54	7.97365
1645.96	8.43644
1646.38	8.95711
1646.8	9.16615
1647.22	8.96263
1647.65	8.54196
1648.07	7.93146
1648.49	7.25006
1648.91	6.54362
1649.33	5.82554
1649.75	5.1869
1650.17	4.52041
1650.59	3.87835
1651.01	3.23516
1651.43	2.5473
1651.86	2.35192
1652.28	2.15442
1652.7	2.09187
1653.12	2.14583
1653.54	2.14491
1653.96	2.19033
1654.38	2.25483
1654.8	2.31519
1655.22	2.40439
1655.64	2.46855
1656.06	2.5549
1656.48	2.51007
1656.9	2.57261
1657.32	2.32004
1657.74	2.09815
1658.16	1.80253
1658.58	1.49083
1659.01	1.24561
1659.43	0.962662

1659.85	0.671659
1660.27	0.443169
1660.69	0.337112
1661.11	0.235577
1661.53	0.326605
1661.95	0.637358
1662.37	1.04805
1662.79	1.3961
1663.21	1.72701
1663.63	2.06598
1664.05	2.37183
1664.47	2.69112
1664.89	2.94803
1665.31	3.21069
1665.73	3.1534
1666.15	3.11074
1666.57	3.10518
1666.98	3.07969
1667.41	2.89741
1667.82	2.66092
1668.24	2.38789
1668.66	2.07426
1669.08	1.75357
1669.5	1.42379
1669.92	1.13165
1670.34	0.797983
1670.76	0.603905
1671.18	0.433428
1671.6	0.451677
1672.02	0.640108
1672.44	0.888687
1672.86	1.13579
1673.28	1.44583
1673.7	2.10512
1674.11	2.30657
1674.54	2.51508
1674.95	2.64881
1675.37	2.57944
1675.79	2.44718
1676.21	2.39166
1676.63	2.26437
1677.05	2.18387
1677.47	2.11372
1677.89	2.06177
1678.31	1.99566
1678.72	1.93328
1679.14	2.00085
1679.56	2.13857
1679.98	2.3668
1680.4	2.53617
1680.82	2.7497
1681.24	2.94742
1681.66	3.08131
1682.08	3.43526
1682.49	3.2908
1682.91	3.1331
1683.33	2.53923
1683.75	2.14054
1684.17	1.70909
1684.59	1.33233
1685	0.955175
1685.42	0.601135

1685.84	0.541172
1686.26	0.739096
1686.68	0.931753
1687.1	1.1155
1687.51	1.31523
1687.93	1.52868
1688.35	1.7554
1688.77	1.96988
1689.19	2.18292
1689.6	2.36907
1690.02	2.52166
1690.44	2.67294
1690.86	2.65578
1691.28	2.51068
1691.69	2.49604
1692.11	2.24808
1692.53	1.96584
1692.95	1.6506
1693.37	1.29902
1693.78	0.960032
1694.2	0.602484
1694.62	0.244709
1695.04	-0.15243
1695.45	-0.476229
1695.87	-0.548477
1696.29	-0.228767
1696.71	0.0160198
1697.12	0.365438
1697.54	0.659891
1697.96	1.026
1698.38	1.33551
1698.79	1.83946
1699.21	2.10429
1699.63	2.15953
1700.04	1.84914
1700.46	1.60221
1700.88	1.37249
1701.3	1.04958
1701.71	0.752923
1702.13	0.53194
1702.55	0.225692
1702.96	0.379439
1703.38	0.931504
1703.8	1.33748
1704.21	1.81933
1704.63	2.28528
1705.05	2.74664
1705.47	3.17349
1705.88	3.57059
1706.3	3.6845
1706.72	3.78572
1707.13	3.79145
1707.55	3.79612
1707.97	3.76838
1708.38	3.57094
1708.8	3.18904
1709.22	2.80513
1709.63	2.36534
1710.05	1.91958
1710.47	1.44102
1710.88	1.01533
1711.3	0.530622

1711.72	0.134437
1712.13	-0.268396
1712.55	-0.245509
1712.96	-0.0696058
1713.38	-0.0108957
1713.8	0.185332
1714.21	0.392301
1714.63	0.630004
1715.05	0.899747
1715.46	1.17999
1715.88	1.49673
1716.29	1.84184
1716.71	2.12904
1717.13	2.4192
1717.54	2.70416
1717.96	2.80744
1718.37	2.87643
1718.79	2.99632
1719.21	3.02942
1719.62	3.07798
1720.04	3.13859
1720.45	3.11441
1720.87	3.01799
1721.28	2.94253
1721.7	2.81412
1722.12	2.70029
1722.53	2.51316
1722.95	2.31669
1723.36	2.09621
1723.78	2.15209
1724.19	2.23257
1724.61	2.44382
1725.02	2.62479
1725.44	2.79005
1725.86	3.05091
1726.27	3.27764
1726.69	3.53321
1727.1	3.79543
1727.52	4.08194
1727.93	4.25834
1728.35	4.45242
1728.76	4.58164
1729.18	4.5845
1729.59	4.6206
1730.01	4.68342
1730.42	4.69644
1730.84	4.71312
1731.25	4.7129
1731.67	4.69476
1732.08	4.67378
1732.5	4.63042
1732.91	4.6133
1733.33	4.69476
1733.74	4.61198
1734.15	4.48137
1734.57	4.36322
1734.98	4.22349
1735.4	4.08366
1735.81	3.9399
1736.23	3.79511
1736.64	3.65308
1737.06	3.50731

1737.47	3.37386
1737.89	3.24047
1738.3	3.10837
1738.71	3.0156
1739.13	2.97377
1739.54	2.92516
1739.96	2.87885
1740.37	2.93517
1740.79	2.97062
1741.2	3.03236
1741.62	3.12287
1742.03	3.32767
1742.44	3.61262
1742.86	3.87297
1743.27	4.1488
1743.68	4.45329
1744.1	4.78223
1744.51	5.08999
1744.93	5.35168
1745.34	5.56646
1745.76	5.80365
1746.17	5.80152
1746.58	5.51499
1747	5.24295
1747.41	4.94424
1747.82	4.57617
1748.24	4.13883
1748.65	3.75417
1749.06	3.54477
1749.48	3.31526
1749.89	3.29887
1750.3	3.25605
1750.72	3.20194
1751.13	3.25007
1751.55	3.34267
1751.96	3.52737
1752.37	3.59635
1752.79	3.61102
1753.2	3.65999
1753.61	3.65678
1754.03	3.69984
1754.44	3.69495
1754.85	3.72772
1755.27	3.7166
1755.68	3.72866
1756.09	3.53643
1756.51	3.51347
1756.92	3.51003
1757.33	3.52966
1757.74	3.6828
1758.16	3.91401
1758.57	4.12405
1758.98	4.58174
1759.4	4.89701
1759.81	5.21757
1760.22	5.53688
1760.63	5.68255
1761.05	5.90236
1761.46	5.87377
1761.87	5.4484
1762.28	5.11595
1762.7	4.75878

1763.11	4.36448
1763.52	4.00618
1763.93	3.69877
1764.35	3.3881
1764.76	3.68592
1765.17	4.16487
1765.58	4.67594
1766	5.16541
1766.41	5.73621
1766.82	6.26659
1767.23	6.71503
1767.65	7.1844
1768.06	7.50826
1768.47	7.51213
1768.88	7.46968
1769.29	7.2169
1769.71	6.69324
1770.12	6.19479
1770.53	5.68315
1770.94	5.19572
1771.35	4.77903
1771.77	4.44282
1772.18	5.05324
1772.59	5.55484
1773	7.95661
1773.41	7.5321
1773.83	7.31019
1774.24	7.34612
1774.65	7.62767
1775.06	8.18853
1775.47	9.00987
1775.88	8.98345
1776.29	7.98315
1776.71	5.90946
1777.12	5.01533
1777.53	4.05706
1777.94	4.10053
1778.35	8.08498
1778.76	9.0735
1779.18	9.36668
1779.59	7.30631
1780	7.53662
1780.41	9.10841
1780.82	9.50644
1781.23	9.67906
1781.64	9.01126
1782.05	8.42929
1782.47	8.0729
1782.88	7.70866
1783.29	7.30939
1783.7	6.92401
1784.11	6.57332
1784.52	6.25331
1784.93	6.10611
1785.34	5.93819
1785.75	5.78591
1786.16	5.64095
1786.58	5.52426
1786.99	5.41956
1787.4	5.38051
1787.81	5.35616
1788.22	5.33439

1788.63	5.24006
1789.04	5.17607
1789.45	5.15668
1789.86	5.22571
1790.27	5.38438
1790.68	5.67898
1791.09	6.02059
1791.5	6.35647
1791.91	6.68252
1792.32	6.94285
1792.73	7.15772
1793.14	7.00299
1793.56	6.88233
1793.96	6.76719
1794.37	6.62162
1794.79	6.37745
1795.2	6.13542
1795.61	5.94149
1796.02	5.74186
1796.43	5.43157
1796.84	5.10247
1797.25	4.84639
1797.66	4.67176
1798.06	4.61175
1798.48	4.79739
1798.89	4.97899
1799.3	5.08074
1799.7	5.11225
1800.12	5.11743
1800.53	5.10325
1800.93	5.0929
1801.34	5.06357
1801.75	4.8602
1802.16	4.62122
1802.57	4.33185
1802.98	4.13761
1803.39	3.92025
1803.8	3.74384
1804.21	3.49262
1804.62	3.22773
1805.03	2.94306
1805.44	2.54246
1805.85	2.07705
1806.26	1.79644
1806.67	1.79881
1807.08	1.80357
1807.49	1.76061
1807.9	1.74863
1808.31	1.69368
1808.31	1.55108
1808.72	0.730838
1809.14	1.03049
1809.55	1.62966
1809.96	2.18659
1810.38	2.67707
1810.79	3.15029
1811.21	3.79174
1811.62	4.30737
1812.03	4.73134
1812.45	4.95279
1812.86	5.13659
1813.28	4.83358

1813.69	4.4548
1814.1	4.27862
1814.52	4.3124
1814.93	4.47965
1815.35	4.31052
1815.76	4.38524
1816.17	4.49988
1816.59	4.63135
1817	4.5701
1817.42	4.78101
1817.83	4.8613
1818.24	4.81052
1818.66	4.99442
1819.07	5.19252
1819.48	5.53337
1819.9	5.74513
1820.31	5.81158
1820.72	6.0099
1821.14	5.96527
1821.55	5.58373
1821.96	5.46002
1822.38	5.10634
1822.79	4.7338
1823.2	4.51345
1823.62	4.24885
1824.03	4.22886
1824.44	4.33235
1824.86	4.33311
1825.27	4.1893
1825.68	4.16164
1826.09	4.11911
1826.51	4.07866
1826.92	4.20516
1827.33	4.51037
1827.75	4.88202
1828.16	5.06451
1828.57	5.2221
1828.99	5.17201
1829.4	4.85555
1829.81	4.643
1830.22	4.33671
1830.64	3.3822
1831.05	2.89955
1831.46	2.77637
1831.87	2.78914
1832.29	2.8323
1832.7	2.98077
1833.11	3.1177
1833.52	3.30057
1833.94	3.59642
1834.35	4.30098
1834.76	4.47758
1835.17	4.69798
1835.59	4.91189
1836	5.2609
1836.41	5.34226
1836.82	5.21576
1837.23	4.6622
1837.65	4.01958
1838.06	3.83374
1838.47	3.80276
1838.88	3.66975

1839.3	3.75305
1839.71	3.7722
1840.12	3.39255
1840.53	3.10887
1840.94	2.7387
1841.36	2.38523
1841.77	2.29567
1842.18	2.30952
1842.59	2.17248
1843	2.13286
1843.41	2.29822
1843.83	2.34905
1844.24	2.69164
1844.65	3.02374
1845.06	3.47398
1845.47	3.91348
1845.88	4.36962
1846.3	4.58336
1846.71	4.7289
1847.12	4.76805
1847.53	4.79803
1847.94	4.65762
1848.35	4.14635
1848.76	3.70614
1849.18	3.18576
1849.59	2.94263
1850	2.87231
1850.41	2.66127
1850.82	2.72355
1851.23	2.98308
1851.64	3.22205
1852.05	3.577
1852.47	4.13262
1852.88	4.43411
1853.29	4.89351
1853.7	5.34458
1854.11	5.73215
1854.52	5.8661
1854.93	5.93226
1855.34	6.01632
1855.75	6.01659
1856.16	5.57151
1856.57	5.06037
1856.99	4.53779
1857.4	4.24652
1857.81	4.19991
1858.22	3.93244
1858.63	3.73075
1859.04	3.71684
1859.45	3.78744
1859.86	4.03984
1860.27	4.08202
1860.68	4.31
1861.09	4.42383
1861.5	4.36216
1861.91	4.46763
1862.32	4.46879
1862.73	4.30255
1863.14	4.38451
1863.55	4.42945
1863.96	4.66954
1864.37	4.82681

1864.79	5.09508
1865.19	5.28132
1865.6	5.52666
1866.02	5.79343
1866.42	5.85294
1866.84	5.62325
1867.25	5.42599
1867.65	5.084
1868.07	5.19853
1868.47	5.13833
1868.88	5.31528
1869.3	5.21167
1869.7	5.11407
1870.11	4.91565
1870.52	4.59238
1870.93	4.13221
1871.34	3.96395
1871.75	3.86325
1872.16	3.79158
1872.57	3.4456
1872.98	3.1677
1873.39	3.19749
1873.8	3.57943
1874.21	3.82887
1874.62	3.95071
1875.03	4.04483
1875.44	4.31502
1875.85	4.53429
1876.26	4.77934
1876.67	4.99432
1877.08	5.22748
1877.49	4.85767
1877.9	4.60124
1878.3	4.14202
1878.71	3.89844
1879.12	3.78208
1879.53	3.60791
1879.94	3.43626
1880.35	3.15465
1880.76	2.98629
1881.17	3.24105
1881.58	3.40433
1881.99	3.49648
1882.4	3.57025
1882.8	3.5821
1883.21	3.68417
1883.62	3.55445
1884.03	3.59724
1884.44	3.76235
1884.85	3.65855
1885.26	3.68504
1885.67	3.45004
1886.07	3.64545
1886.48	4.08472
1886.89	4.60609
1887.3	5.01362
1887.71	5.20697
1888.12	5.32773
1888.53	5.41628
1888.93	5.41624
1889.34	5.19093
1889.75	4.40569

1890.16	3.919
1890.57	3.4236
1890.97	2.97848
1891.38	2.75167
1891.79	2.77378
1892.2	2.98371
1892.61	3.30184
1893.02	3.28008
1893.42	3.63574
1893.83	3.9154
1894.24	4.15652
1894.65	4.66247
1895.06	5.10171
1895.46	5.58047
1895.87	5.96888
1896.28	6.2871
1896.69	6.29221
1897.1	6.15338
1897.51	6.23614
1897.91	6.20095
1898.32	6.5092
1898.73	6.58335
1899.13	6.54144
1899.54	6.10789
1899.95	5.67634
1900.36	4.99524
1900.77	4.16397
1901.17	3.41288
1901.58	2.98989
1901.99	2.88702
1902.4	2.80673
1902.8	2.92671
1903.21	2.86986
1903.62	2.87434
1904.03	2.83097
1904.43	3.00433
1904.84	3.13552
1905.25	3.48292
1905.66	3.75905
1906.06	3.85279
1906.47	4.07967
1906.88	4.10882
1907.28	3.89694
1907.69	3.51857
1908.1	3.35594
1908.5	3.27616
1908.91	3.03874
1909.32	2.87837
1909.72	2.72803
1910.13	2.69898
1910.54	2.58199
1910.95	2.61799
1911.35	2.72458
1911.76	2.86959
1912.17	3.06589
1912.57	3.32268
1912.98	3.58757
1913.39	3.93838
1913.79	4.10496
1914.2	4.2229
1914.61	4.22452
1915.01	4.12537

1915.42	3.92206
1915.83	3.87363
1916.23	3.62717
1916.64	3.2083
1917.05	2.81858
1917.45	2.6067
1917.86	2.71608
1918.26	2.54208
1918.67	2.11821
1919.08	2.41409
1919.48	3.33759
1919.89	3.74574
1920.29	4.92834
1920.7	5.33136
1921.11	5.398
1921.51	4.38791
1921.92	3.99526
1922.32	3.56869
1922.73	3.33665
1923.14	3.23176
1923.54	3.36659
1923.95	3.44497
1924.36	3.43862
1924.76	3.36243
1925.17	3.37504
1925.57	3.53562
1925.98	3.56087
1926.38	3.56353
1926.79	3.52281
1927.19	3.51553
1927.6	3.53242
1928.01	3.40918
1928.41	3.54966
1928.82	3.61844
1929.22	3.60596
1929.63	3.89471
1930.03	4.30907
1930.44	4.86288
1930.84	5.27948
1931.25	5.59523
1931.66	5.84693
1932.06	5.84526
1932.47	5.86854
1932.87	5.80042
1933.28	6.03936
1933.68	6.26106
1934.09	6.17351
1934.49	5.90104
1934.9	5.73458
1935.3	5.45623
1935.71	5.28214
1936.11	5.09645
1936.52	4.85851
1936.92	4.66334
1937.33	4.56832
1937.73	4.48745
1938.14	4.32887
1938.54	4.14429
1938.95	3.93105
1939.35	3.61166
1939.76	3.31298
1940.16	3.2204

1940.57	3.52298
1940.97	3.45771
1941.37	3.3761
1941.78	3.09418
1942.18	2.80319
1942.59	2.62995
1942.99	2.73505
1943.4	2.98545
1943.8	3.1679
1944.21	3.40288
1944.61	3.53277
1945.01	3.83709
1945.42	4.23796
1945.82	4.37537
1946.23	4.62554
1946.63	4.65888
1947.04	4.48946
1947.44	4.18308
1947.84	4.07906
1948.25	4.11895
1948.65	3.77557
1949.06	2.56469
1949.46	1.97892
1949.86	1.67865
1950.27	1.77542
1950.67	2.30329
1951.08	3.06177
1951.48	3.7905
1951.88	4.50081
1952.29	4.88251
1952.69	5.10088
1953.1	5.17608
1953.5	5.69975
1953.9	5.94663
1954.31	6.16335
1954.71	6.45946
1955.11	6.60628
1955.52	6.90084
1955.92	7.04176
1956.33	6.84159
1956.73	6.70864
1957.13	6.58495
1957.54	6.38616
1957.94	6.24752
1958.34	6.14207
1958.75	6.00306
1959.15	5.74468
1959.55	5.50179
1959.96	5.24308
1960.36	5.19613
1960.76	5.11428
1961.17	4.82864
1961.57	4.52134
1961.97	4.08458
1962.38	3.6961
1962.78	3.62296
1963.18	3.48675
1963.58	3.63995
1963.99	3.75356
1964.39	3.9355
1964.79	4.31455
1965.19	4.57154

1965.6	4.56939
1966	4.76916
1966.4	4.90499
1966.81	5.16582
1967.21	5.21735
1967.61	5.29937
1968.01	5.15391
1968.42	4.866
1968.82	4.37683
1969.22	4.06957
1969.63	4.0778
1970.03	3.9052
1970.43	4.03955
1970.83	4.16015
1971.23	4.32668
1971.64	4.8099
1972.04	5.11072
1972.44	5.34956
1972.84	5.45229
1973.25	5.61863
1973.65	5.72161
1974.05	5.79355
1974.45	5.64199
1974.85	5.6294
1975.26	5.46952
1975.66	5.25014
1976.06	4.99677
1976.46	4.89102
1976.87	4.72691
1977.27	4.30627
1977.67	4.00999
1978.07	3.6683
1978.47	3.52998
1978.88	3.43264
1979.28	3.39958
1979.68	3.65826
1980.08	3.65884
1980.48	3.6746
1980.88	3.97352
1981.29	3.88741
1981.69	3.74244
1982.09	3.70213
1982.49	3.57003
1982.89	3.471
1983.29	3.18038
1983.7	2.92256
1984.1	2.64564
1984.5	2.86196
1984.9	2.77246
1985.3	2.71375
1985.7	2.79542
1986.1	2.85523
1986.51	2.88012
1986.91	2.85843
1987.31	2.79255
1987.71	2.76728
1988.11	2.6239
1988.51	2.25654
1988.91	2.25672
1989.31	2.05102
1989.72	2.07812
1990.12	2.3242

1990.52	2.73093
1990.92	3.28717
1991.32	3.51366
1991.72	3.82214
1992.12	3.97089
1992.52	4.27093
1992.92	4.34804
1993.32	4.34868
1993.72	4.55624
1994.13	4.49934
1994.53	4.52436
1994.93	4.35613
1995.33	4.49134
1995.73	4.57455
1996.13	4.55875
1996.53	4.38541
1996.93	4.6075
1997.33	4.93223
1997.73	5.31319
1998.13	5.55852
1998.53	5.41943
1998.93	5.28579
1999.33	5.02465
1999.73	4.70328
2000.13	4.2192
2000.53	3.88507
2000.93	3.67057
2001.33	3.69413
2001.74	3.60467
2002.13	3.36649
2002.53	3.29195
2002.94	3.38915
2003.33	3.56347
2003.74	3.89761
2004.14	4.06277
2004.53	4.11632
2004.94	4.37961
2005.34	4.81631
2005.73	5.09976
2006.14	5.29223
2006.54	5.2555
2006.93	5.24748
2007.33	4.91998
2007.73	4.69202
2008.13	4.55714
2008.53	4.44478
2008.93	4.42786
2009.33	4.59002
2009.73	4.47837
2010.13	4.2293
2010.53	4.24268
2010.93	4.16491
2011.33	4.21215
2011.73	4.22787
2012.13	4.21924
2012.53	4.21404
2012.93	4.5733
2013.33	5.00366
2013.73	5.35396
2014.13	5.49271
2014.53	5.48071
2014.92	5.35362

2015.32	4.88934
2015.72	4.56802
2016.12	4.4731
2016.52	4.19838
2016.92	3.77257
2017.32	3.44016
2017.72	2.91881
2018.12	2.47593
2018.52	2.39407
2018.92	2.21014
2019.32	2.27336
2019.71	2.52652
2020.11	2.91015
2020.51	3.41247
2020.91	3.8154
2021.31	4.00088
2021.71	4.22311
2022.11	4.37494
2022.51	4.54856
2022.9	4.57243
2023.3	4.36577
2023.7	4.23183
2024.1	3.90747
2024.5	3.39665
2024.9	2.94821
2025.3	2.6146
2025.69	2.2694
2026.09	1.89253
2026.49	1.83086
2026.89	1.77855
2027.29	2.19341
2027.69	2.35069
2028.08	2.41381
2028.48	2.59842
2028.88	2.54656
2029.28	2.32305
2029.68	2.135
2030.07	2.15237
2030.47	1.92412
2030.87	1.7756
2031.27	1.82516
2031.67	1.75169
2032.07	1.65499
2032.46	1.84087
2032.86	1.85937
2033.26	1.85347
2033.66	1.86364
2034.06	1.86158
2034.45	2.1095
2034.85	1.82341
2035.25	2.0859
2035.65	2.27812
2036.04	2.58223
2036.44	3.10516
2036.84	3.5798
2037.24	4.07889
2037.63	4.62865
2038.03	4.84411
2038.43	4.85903
2038.83	4.61698
2039.22	4.52999
2039.62	4.15867

2040.02	3.36005
2040.42	3.03936
2040.81	2.41099
2041.21	2.06761
2041.61	1.78418
2042.01	1.52261
2042.4	1.52137
2042.8	1.3898
2043.2	1.351
2043.59	1.47793
2043.99	1.74262
2044.39	2.24887
2044.78	2.72074
2045.18	3.28644
2045.58	3.78427
2045.98	4.3756
2046.37	4.7312
2046.77	4.8884
2047.17	4.86931
2047.56	4.92743
2047.96	4.90608
2048.36	4.70592
2048.76	4.24061
2049.15	3.94934
2049.55	4.01928
2049.95	4.03955
2050.34	4.35027
2050.74	4.48858
2051.14	4.60969
2051.53	4.83868
2051.93	5.42073
2052.32	5.93727
2052.72	6.1791
2053.12	6.25508
2053.51	6.25358
2053.91	6.14992
2054.31	5.96029
2054.7	5.385
2055.1	4.97527
2055.5	4.41604
2055.89	3.97669
2056.29	3.58798
2056.68	3.2599
2057.08	2.85539
2057.48	2.60206
2057.87	2.32084
2058.27	2.22023
2058.66	2.24768
2059.06	1.95615
2059.46	1.63582
2059.85	1.60705
2060.25	1.60021
2060.64	1.41562
2061.04	1.3165
2061.44	1.41434
2061.83	1.54164
2062.23	1.92397
2062.62	2.0068
2063.02	2.19012
2063.41	2.1685
2063.81	1.9716
2064.21	1.87875

2064.6	1.74534
2065	1.47612
2065.39	1.31404
2065.79	1.01838
2066.18	0.713559
2066.58	0.5878
2066.97	0.34481
2067.37	-0.03403
2067.77	-0.0287828
2068.16	0.169771
2068.56	0.403205
2068.95	0.965586
2069.35	1.73419
2069.74	2.47088
2070.14	3.0514
2070.53	3.23403
2070.93	3.31647
2071.32	3.10988
2071.72	2.84222
2072.11	2.47777
2072.51	2.06436
2072.9	1.88413
2073.3	1.71074
2073.69	1.54315
2074.09	1.54076
2074.48	1.30338
2074.88	1.27142
2075.27	1.10616
2075.67	1.11107
2076.06	1.11746
2076.46	1.10849
2076.85	1.1905
2077.25	1.2877
2077.64	1.49832
2078.03	1.79263
2078.43	2.21104
2078.82	2.58537
2079.22	2.51522
2079.61	2.4497
2080.01	2.36
2080.4	2.53442
2080.8	2.82876
2081.19	2.59489
2081.59	2.55708
2081.98	2.27444
2082.37	1.84262
2082.77	1.50167
2083.16	1.12319
2083.56	0.666663
2083.95	0.0217228
2084.34	-0.317363
2084.74	-0.713309
2085.13	-0.895986
2085.53	-0.626572
2085.92	-0.676543
2086.31	-0.397597
2086.71	-0.264979
2087.1	-0.0405588
2087.5	0.365562
2087.89	0.70439
2088.28	1.17271
2088.68	1.53857

2089.07	1.75886
2089.47	2.19967
2089.86	2.41666
2090.25	2.65387
2090.65	2.84887
2091.04	2.98601
2091.43	3.14461
2091.83	3.26387
2092.22	3.41094
2092.62	3.31399
2093.01	3.53618
2093.4	3.59027
2093.8	3.69372
2094.19	3.83834
2094.58	3.84803
2094.98	3.42784
2095.37	3.05648
2095.76	3.09688
2096.16	1.88152
2096.55	0.767948
2096.94	-0.570443
2097.33	-1.21634
2097.73	1.27972
2098.12	1.41721
2098.51	1.43051
2098.91	1.37764
2099.3	1.22641
2099.69	1.09306
2100.09	1.07549
2100.48	1.06448
2100.87	1.18476
2101.27	1.40599
2101.66	1.64327
2102.05	2.03852
2102.44	2.24849
2102.84	2.1189
2103.23	2.02961
2103.62	1.94557
2104.02	1.76399
2104.41	1.71319
2104.8	1.51613
2105.19	1.50648
2105.59	1.37997
2105.98	1.0872
2106.37	0.868666
2106.76	0.686872
2107.16	0.366544
2107.55	0.185287
2107.94	0.348628
2108.33	0.615959
2108.73	0.982564
2109.12	1.49661
2109.51	1.69304
2109.9	1.94069
2110.29	2.10355
2110.69	2.11492
2111.08	2.22148
2111.47	2.24581
2111.86	2.28879
2112.26	2.21233
2112.65	1.85415
2113.04	1.67991

2113.43	1.58774
2113.82	1.76069
2114.22	1.73381
2114.61	1.78491
2115	1.98341
2115.39	2.54025
2115.78	3.14281
2116.17	3.35754
2116.57	3.56139
2116.96	3.61268
2117.35	3.64041
2117.74	3.81595
2118.13	3.92451
2118.53	3.76892
2118.92	3.76183
2119.31	3.82432
2119.7	3.61485
2120.09	3.3922
2120.48	3.47275
2120.87	3.45918
2121.27	3.6187
2121.66	3.80641
2122.05	3.82896
2122.44	3.88147
2122.83	3.65656
2123.22	3.45097
2123.61	3.07763
2124.01	2.52706
2124.4	2.30777
2124.79	2.07815
2125.18	1.93188
2125.57	1.98745
2125.96	2.23535
2126.35	2.2272
2126.74	2.3656
2127.14	2.46366
2127.52	2.4439
2127.92	2.73026
2128.31	3.21872
2128.7	3.8274
2129.09	4.51434
2129.48	5.26268
2129.87	5.53073
2130.26	5.8675
2130.65	5.74817
2131.04	6.0452
2131.43	6.13942
2131.82	6.16251
2132.21	6.29309
2132.61	6.51744
2133	6.75074
2133.39	6.8256
2133.78	7.18011
2134.17	7.67142
2134.56	8.44566
2134.95	8.99193
2135.34	9.50506
2135.73	10.3513
2136.12	11.4794
2136.51	12.1669
2136.9	13.2925
2137.29	13.7785

2137.68	13.6759
2138.07	13.615
2138.46	13.3277
2138.85	12.994
2139.24	12.8142
2139.63	12.557
2140.02	11.8652
2140.41	11.2443
2140.8	10.6561
2141.19	10.3322
2141.58	10.2297
2141.97	10.2177
2142.36	10.3761
2142.75	10.8277
2143.14	11.2471
2143.53	11.5246
2143.92	11.6584
2144.31	11.6828
2144.7	11.75
2145.09	11.6475
2145.48	10.7188
2145.87	10.6971
2146.26	10.6761
2146.65	10.7161
2147.04	10.738
2147.43	10.895
2147.82	11.0148
2148.21	11.0804
2148.59	11.3119
2148.99	11.5393
2149.37	11.8062
2149.76	11.9617
2150.15	12.0715
2150.54	12.0643
2150.93	12.0085
2151.32	11.8051
2151.71	11.6962
2152.1	11.3976
2152.49	11.0896
2152.88	11.1373
2153.27	11.3026
2153.66	11.6712
2154.04	12.0078
2154.43	12.3445
2154.82	12.7732
2155.21	12.967
2155.6	13.0793
2155.99	13.3207
2156.38	13.2815
2156.77	13.1891
2157.16	13.0896
2157.54	12.9399
2157.93	12.9452
2158.32	12.8694
2158.71	12.6411
2159.1	12.415
2159.49	12.1482
2159.88	11.6901
2160.27	11.3495
2160.65	11.0661
2161.04	10.7822
2161.43	10.6188

2161.82	10.5253
2162.21	10.4443
2162.6	10.264
2162.98	10.0711
2163.37	9.87364
2163.76	9.70335
2164.15	9.49989
2164.54	9.25544
2164.92	9.10814
2165.31	8.75555
2165.7	8.14761
2166.09	7.54908
2166.48	7.0337
2166.87	6.70886
2167.25	6.43188
2167.64	6.30676
2168.03	6.19671
2168.42	6.17858
2168.81	6.09001
2169.19	5.95667
2169.58	5.78298
2169.97	5.54656
2170.36	5.27106
2170.74	5.00132
2171.13	5.07303
2171.52	5.42175
2171.91	5.88732
2172.3	6.4731
2172.68	6.31704
2173.07	5.81018
2173.46	5.25201
2173.85	4.70888
2174.23	4.17114
2174.62	3.65694
2175.01	3.12531
2175.4	2.63512
2175.78	1.97181
2176.17	1.45451
2176.56	1.23466
2176.95	1.05431
2177.33	0.938644
2177.72	0.881193
2178.11	0.835512
2178.5	0.798198
2178.88	0.774254
2179.27	0.772242
2179.66	0.754724
2180.04	0.747265
2180.43	0.715651
2180.67	0.718986
2181.06	0.90993
2181.45	1.06755
2181.85	1.24604
2182.24	1.22414
2182.63	1.25077
2183.02	1.31831
2183.41	1.36551
2183.8	1.40381
2184.2	1.44158
2184.59	1.45798
2184.98	1.49237
2185.37	1.49571

2185.76	1.53072
2186.15	1.52879
2186.55	1.56201
2186.94	1.55988
2187.33	1.58959
2187.72	1.58464
2188.11	1.62819
2188.5	1.62403
2188.89	1.67939
2189.29	1.5871
2189.68	1.52359
2190.07	1.41143
2190.46	1.3064
2190.85	1.17157
2191.24	1.02827
2191.63	0.872735
2192.02	0.547436
2192.42	0.396911
2192.81	0.349344
2193.2	0.31933
2193.59	0.307595
2193.98	0.314459
2194.37	0.585566
2194.76	0.778858
2195.15	0.901921
2195.54	1.07196
2195.94	1.21914
2196.33	1.36449
2196.72	1.50089
2197.11	1.62072
2197.5	1.75131
2197.89	1.76105
2198.28	1.81314
2198.67	1.88512
2199.06	1.91022
2199.45	1.96003
2199.84	2.01249
2200.23	2.05274
2200.62	2.09687
2201.02	2.12892
2201.4	2.16698
2201.8	2.18827
2202.19	2.29603
2202.58	2.28242
2202.97	2.28071
2203.36	2.24756
2203.75	2.22938
2204.14	2.18049
2204.53	2.14636
2204.92	2.08789
2205.31	2.03198
2205.7	1.98294
2206.09	1.92429
2206.48	1.87927
2206.87	1.82596
2207.26	1.79014
2207.65	1.75041
2208.04	1.72913
2208.43	1.71148
2208.82	1.72519
2209.21	1.74753
2209.6	1.79901

2209.99	1.86908
2210.38	1.9555
2210.77	2.26169
2211.16	2.41721
2211.55	2.62014
2211.94	2.92882
2212.33	3.15662
2212.72	3.4111
2213.11	3.66317
2213.5	3.93226
2213.89	4.17682
2214.28	4.26171
2214.67	4.27232
2215.06	4.09357
2215.44	3.71818
2215.83	3.42106
2216.22	3.1259
2216.61	2.8391
2217	2.55005
2217.39	2.27081
2217.78	1.9963
2218.17	1.74709
2218.56	1.4854
2218.95	1.48199
2219.34	1.90182
2219.73	2.14504
2220.12	2.44997
2220.51	2.78216
2220.9	3.13467
2221.28	3.48186
2221.67	3.87257
2222.06	4.10071
2222.45	4.26001
2222.84	4.2181
2223.23	4.10662
2223.62	3.96825
2224.01	3.80297
2224.4	3.63032
2224.78	3.44646
2225.17	3.2694
2225.56	3.0856
2225.95	2.92005
2226.34	2.75566
2226.73	2.62145
2227.12	2.68674
2227.51	2.61632
2227.89	2.66261
2228.28	2.67424
2228.67	2.66017
2229.06	2.61868
2229.45	2.5302
2229.84	1.94515
2230.23	1.55195
2230.61	1.24289
2231	0.946995
2231.39	0.707152
2231.78	0.581819
2232.17	0.623768
2232.55	0.875904
2232.94	1.29167
2233.33	1.49401
2233.72	1.73369

2234.11	2.03106
2234.5	2.3676
2234.88	2.72548
2235.27	3.11805
2235.66	3.51146
2236.05	3.95358
2236.44	3.47585
2236.82	3.01384
2237.21	2.82385
2237.6	2.90305
2237.99	3.29107
2238.38	4.04053
2238.76	4.89327
2239.15	5.35262
2239.54	5.7184
2239.93	6.00578
2240.31	3.66536
2240.7	3.08308
2241.09	2.80923
2241.48	2.60376
2241.87	2.43573
2242.25	2.30362
2242.64	2.19442
2243.03	2.11233
2243.42	2.04415
2243.8	1.99428
2244.19	1.95341
2244.58	1.91093
2244.96	1.8908
2245.35	1.85664
2245.74	1.84934
2246.13	1.82033
2246.51	1.82141
2246.9	1.79754
2247.29	1.80443
2247.68	1.78693
2248.06	1.79881
2248.45	1.78965
2248.84	1.80681
2249.22	1.80878
2249.61	1.83332
2250	1.84826
2250.38	1.88221
2250.77	1.9115
2251.16	1.95043
2251.55	2.0022
2251.93	2.05598
2252.32	2.12229
2252.71	2.19073
2253.09	2.26377
2253.48	2.35634
2253.87	2.44588
2254.25	2.56054
2254.64	2.64515
2255.03	2.79427
2255.41	2.92556
2255.8	3.04464
2256.19	3.26646
2256.57	3.48435
2256.96	3.71655
2257.35	3.94664
2257.73	4.16814

2258.12	4.38971
2258.51	4.59822
2258.89	4.77449
2259.28	4.89723
2259.67	5.00437
2260.05	4.44189
2260.44	3.8088
2260.82	3.27587
2261.21	2.6286
2261.6	2.0113
2261.98	1.43015
2262.37	0.906327
2262.75	0.451519
2263.14	0.381973
2263.53	0.366277
2263.91	0.379961
2264.3	0.756522
2264.68	1.20107
2265.07	1.64566
2265.46	2.11682
2265.84	2.59681
2266.23	3.0657
2266.61	3.50894
2267	3.86613
2267.38	3.95818
2267.77	4.08701
2268.16	4.12654
2268.54	3.73913
2268.93	3.30874
2269.31	2.83726
2269.7	2.34778
2270.08	1.87216
2270.47	1.37177
2270.86	0.875094
2271.24	0.418805
2271.63	-0.0556185
2272.01	-0.479514
2272.4	-0.741084
2272.78	-1.00404
2273.17	-1.21084
2273.55	-1.32828
2273.94	-1.43524
2274.32	-1.5305
2274.71	-1.57958
2275.09	-1.59739
2275.48	-1.43025
2275.86	-1.23913
2276.25	-1.00154
2276.63	-0.772781
2277.02	-0.500005
2277.41	-0.229646
2277.79	0.0640209
2278.18	0.363128
2278.56	0.664635
2278.95	0.979509
2279.33	1.27647
2279.71	1.59309
2280.1	1.87322
2280.48	2.17609
2280.87	2.29681
2281.25	2.43142
2281.64	2.55094

2282.02	2.64795
2282.41	2.6905
2282.79	2.65537
2283.18	2.61769
2283.56	2.59096
2283.95	2.49909
2284.33	2.37984
2284.72	2.2656
2285.1	2.1002
2285.49	1.92626
2285.87	1.73515
2286.25	1.5308
2286.64	1.31779
2287.02	1.09321
2287.41	0.867043
2287.79	0.90744
2288.17	0.825237
2288.56	1.05227
2288.94	1.27974
2289.33	1.51008
2289.71	1.75371
2290.1	2.02266
2290.48	2.29186
2290.86	2.57836
2291.25	2.86364
2291.63	3.14849
2292.02	3.42827
2292.4	3.66591
2292.78	3.87693
2293.17	3.68232
2293.55	3.52028
2293.94	3.16002
2294.32	2.83354
2294.7	2.27777
2295.09	1.95594
2295.47	1.8083
2295.85	1.61254
2296.24	1.33381
2296.62	0.972644
2297.01	0.535558
2297.39	-0.238635
2297.77	0.141577
2298.16	0.606225
2298.54	1.0814
2298.92	1.47458
2299.31	1.89272
2299.69	2.24902
2300.07	2.76929
2300.46	2.812
2300.84	2.8067
2301.22	2.77852
2301.61	2.696
2301.99	2.61757
2302.37	2.49641
2302.76	2.39057
2303.14	2.19965
2303.52	2.10212
2303.91	1.9805
2304.29	1.77279
2304.67	1.736
2305.06	1.71652
2305.44	1.71993

2305.82	1.7319
2306.2	1.84573
2306.59	1.96866
2306.97	2.08241
2307.35	2.17571
2307.74	2.27605
2308.12	2.47622
2308.5	2.5801
2308.88	2.63915
2309.27	2.6845
2309.65	2.71892
2310.03	2.74578
2310.42	2.74729
2310.8	2.7458
2311.18	2.719
2311.56	2.70829
2311.95	2.51089
2312.33	2.45839
2312.71	2.36254
2313.09	2.30171
2313.48	2.19978
2313.86	2.13397
2314.24	2.06144
2314.62	2.00927
2315.01	1.947
2315.39	1.90883
2315.77	1.8622
2316.15	1.8366
2316.53	1.87167
2316.92	1.84131
2317.3	1.85679
2317.68	1.83645
2318.06	1.85076
2318.44	1.84104
2318.83	1.80767
2319.21	1.8239
2319.59	1.86611
2319.97	1.89474
2320.35	1.94847
2320.74	1.99424
2321.12	2.0584
2321.5	2.12696
2321.88	2.19908
2322.26	2.28739
2322.65	2.37472
2323.03	2.46654
2323.41	2.55291
2323.79	2.86432
2324.17	2.86482
2324.55	2.8623
2324.94	2.84264
2325.32	2.81814
2325.7	2.77212
2326.08	2.42124
2326.46	2.3074
2326.84	2.16818
2327.22	2.06026
2327.61	1.94598
2327.99	1.82917
2328.37	1.68326
2328.75	1.65705
2329.13	1.65197

2329.51	1.6562
2329.89	1.68693
2330.27	1.90156
2330.66	1.99821
2331.04	2.11862
2331.42	2.23594
2331.8	2.3684
2332.18	2.49806
2332.56	2.6345
2332.94	2.76851
2333.32	2.90221
2333.71	3.01785
2334.09	3.14281
2334.47	3.28664
2334.85	3.39631
2335.23	3.48293
2335.61	3.57383
2335.99	3.68804
2336.37	3.7507
2336.75	3.79533
2337.13	3.83395
2337.51	3.86147
2337.89	3.84828
2338.28	3.79449
2338.66	3.76764
2339.04	3.71178
2339.42	3.65944
2339.8	3.584
2340.18	3.51225
2340.56	3.42336
2340.94	3.35117
2341.32	3.25239
2341.7	3.15617
2342.08	3.06008
2342.46	2.95668
2342.84	2.85443
2343.22	2.74767
2343.6	2.64239
2343.98	2.53619
2344.36	2.33823
2344.74	2.24477
2345.12	2.17877
2345.5	2.10736
2345.88	2.06505
2346.26	2.01996
2346.64	2.00434
2347.02	1.98916
2347.4	1.98954
2347.78	2.01874
2348.16	2.05108
2348.54	2.10986
2348.92	2.19981
2349.3	2.29077
2349.68	2.36387
2350.06	2.47628
2350.44	2.6208
2350.82	2.75625
2351.2	2.88271
2351.58	3.0332
2351.96	3.22845
2352.34	3.35728
2352.72	3.49707

2353.1	3.61648
2353.48	3.75008
2353.86	3.85331
2354.24	3.96577
2354.62	4.02047
2355	4.10802
2355.38	4.21913
2355.75	4.36629
2356.13	4.47107
2356.51	4.59307
2356.89	4.68226
2357.27	4.77017
2357.65	4.8323
2358.03	4.87499
2358.41	4.89483
2358.79	4.87902
2359.17	4.86466
2359.55	4.75614
2359.93	4.69884
2360.31	4.27724
2360.68	3.49552
2361.06	2.86373
2361.44	2.2022
2361.82	1.50875
2362.2	0.843148
2362.58	0.169521
2362.96	-0.445521
2363.34	-1.12709
2363.72	-1.43685
2364.09	-1.33964
2364.47	-1.39994
2364.85	-1.32252
2365.23	-1.26719
2365.61	-1.16804
2365.99	-1.04387
2366.37	-0.907657
2366.74	-0.739248
2367.12	-0.579977
2367.5	-0.405956
2367.88	-0.258371
2368.26	-0.19715
2368.64	-0.100489
2369.02	-0.12312
2369.39	0.103569
2369.77	0.349849
2370.15	0.598625
2370.53	0.87165
2370.91	1.11332
2371.29	1.39724
2371.66	1.62925
2372.04	1.90983
2372.42	2.1253
2372.8	2.59424
2373.18	2.7399
2373.56	2.86622
2373.93	2.97953
2374.31	3.06118
2374.69	3.14262
2375.07	3.18788
2375.45	3.04987
2375.82	3.05186
2376.2	3.01903

2376.58	2.97172
2376.96	2.95022
2377.33	2.93377
2377.71	2.92626
2378.09	2.91703
2378.47	2.92714
2378.85	2.92918
2379.22	2.95563
2379.6	2.96821
2379.98	3.00622
2380.36	3.02237
2380.73	3.06132
2381.11	3.08077
2381.49	3.11945
2381.87	3.19119
2382.24	3.27474
2382.62	3.32324
2383	3.35403
2383.38	3.42534
2383.75	3.43953
2384.13	3.46912
2384.51	3.46897
2384.88	3.48865
2385.26	3.47235
2385.64	3.58385
2386.02	3.5404
2386.39	3.48194
2386.77	3.41935
2387.15	3.35258
2387.52	3.27233
2387.9	3.19809
2388.28	3.08406
2388.66	2.97101
2389.03	2.85197
2389.41	2.72942
2389.79	2.60601
2390.16	2.48413
2390.54	2.36558
2390.92	2.25215
2391.29	2.14585
2391.67	2.04793
2392.05	1.95939
2392.43	1.89782
2392.8	1.83193
2393.18	1.79101
2393.55	1.75138
2393.93	1.73165
2394.31	1.71851
2394.68	1.71923
2395.06	1.84352
2395.44	1.85962
2395.81	1.9395
2396.19	1.97329
2396.57	2.04272
2396.94	2.08728
2397.32	2.18783
2397.7	2.24202
2398.07	2.30419
2398.45	2.36085
2398.82	2.41481
2399.2	2.4715
2399.58	2.49543

2399.95	2.51186
2400.33	2.57059
2400.71	2.62795
2401.08	2.68484
2401.46	2.65825
2401.83	2.71195
2402.21	2.73443
2402.59	2.77632
2402.96	2.79642
2403.34	2.82239
2403.71	2.83604
2404.09	2.83022
2404.47	2.83212
2404.84	2.79413
2405.22	2.79865
2405.59	2.7029
2405.97	2.61773
2406.34	2.57266
2406.72	2.32596
2407.1	2.07269
2407.47	1.47542
2407.85	1.07358
2408.22	0.681088
2408.6	0.319492
2408.97	-0.0158194
2409.35	-0.33505
2409.72	-0.602331
2410.1	-0.780371
2410.48	-0.895072
2410.85	-0.348182
2411.23	-0.0835066
2411.6	0.566296
2411.98	1.20668
2412.35	1.83876
2412.73	2.51363
2413.1	3.16297
2413.48	3.75303
2413.85	4.26187
2414.23	4.27177
2414.6	4.22221
2414.98	3.8255
2415.35	3.32608
2415.73	2.77967
2416.1	2.24712
2416.48	1.70196
2416.85	1.19099
2417.23	0.714926
2417.6	0.553208
2417.98	0.473245
2418.35	0.428518
2418.73	0.758438
2419.1	1.17515
2419.48	1.59156
2419.85	2.0915
2420.23	2.59402
2420.6	3.03453
2420.98	3.495
2421.35	4.05426
2421.72	4.27505
2422.1	4.26573
2422.48	4.26056
2422.85	4.20143

2423.22	4.07921
2423.6	3.94727
2423.97	3.77094
2424.35	3.62059
2424.72	3.42872
2425.09	3.24848
2425.47	3.06015
2425.85	2.86132
2426.22	2.69371
2426.59	2.60763
2426.97	2.50184
2427.34	2.41025
2427.72	2.28672
2428.09	2.08935
2428.46	1.90891
2428.84	1.65188
2429.21	1.38252
2429.59	1.05659
2429.96	0.688285
2430.33	0.323923
2430.71	-0.066847
2431.08	-0.404885
2431.46	-0.424709
2431.83	0.0515563
2432.2	0.232688
2432.58	0.554938
2432.95	0.993843
2433.33	1.58381
2433.7	2.25862
2434.07	2.82658
2434.45	3.26615
2434.82	3.54901
2435.19	3.81491
2435.57	3.73451
2435.94	3.60362
2436.31	3.43635
2436.69	3.22381
2437.06	2.98769
2437.44	2.69133
2437.81	2.54769
2438.18	2.43396
2438.55	2.32779
2438.93	2.25066
2439.3	2.19428
2439.68	2.16808
2440.05	2.18195
2440.42	2.22066
2440.79	2.31794
2441.17	2.51993
2441.54	2.4674
2441.91	2.38594
2442.29	2.29614
2442.66	2.20911
2443.03	2.118
2443.41	2.03389
2443.78	1.9383
2444.15	1.82884
2444.52	1.75011
2444.9	1.72734
2445.27	1.64055
2445.64	1.56315
2446.02	1.60591

2446.39	1.68528
2446.76	1.76546
2447.13	1.86455
2447.51	1.97534
2447.88	2.08886
2448.25	2.22158
2448.63	2.34219
2449	2.48892
2449.37	2.608
2449.74	2.75397
2450.12	2.91996
2450.49	3.03947
2450.86	3.22675
2451.23	3.2306
2451.61	3.25322
2451.98	3.22229
2452.35	3.20947
2452.72	3.02364
2453.1	2.90098
2453.47	2.76375
2453.84	2.59891
2454.21	2.45865
2454.58	2.26118
2454.96	2.14862
2455.33	1.85752
2455.7	1.80144
2456.07	1.74632
2456.44	1.70902
2456.82	1.67029
2457.19	1.64755
2457.56	1.88869
2457.93	1.97942
2458.31	2.03851
2458.68	2.12577
2459.05	2.20128
2459.42	2.22361
2459.79	2.26823
2460.16	2.26153
2460.54	2.27838
2460.91	2.24548
2461.28	2.23095
2461.65	2.0054
2462.02	1.93039
2462.39	1.82797
2462.77	1.73962
2463.14	1.6426
2463.51	1.5584
2463.88	1.51003
2464.25	1.44767
2464.62	1.42551
2464.99	1.39869
2465.37	1.39512
2465.74	1.39131
2466.11	1.40014
2466.48	1.41036
2466.85	1.44015
2467.22	1.46952
2467.59	1.65498
2467.97	1.81176
2468.34	2.08361
2468.71	2.437
2469.08	4.22263

2469.45	4.52298
2469.82	4.56491
2470.19	3.92209
2470.56	3.32639
2470.93	2.91659
2471.31	2.60586
2471.68	2.6905
2472.05	2.86242
2472.42	3.0441
2472.79	3.18547
2473.16	3.28216
2473.53	3.3211
2473.9	3.32792
2474.27	3.32495
2474.64	3.35469
2475.01	3.44166
2475.38	4.00313
2475.75	4.04676
2476.13	4.06767
2476.5	4.08364
2476.87	4.08914
2477.24	4.09248
2477.61	4.09355
2477.98	4.08963
2478.35	4.09138
2478.72	4.08358
2479.09	3.95724
2479.46	4.00324
2479.83	4.03768
2480.2	4.09852
2480.57	4.14209
2480.94	4.21944
2481.31	4.27552
2481.68	4.3743
2482.05	4.45169
2482.42	4.56288
2482.79	4.65828
2483.16	4.76802
2483.53	4.90125
2483.9	4.99042
2484.27	5.09574
2484.64	5.19627
2485.01	5.36454
2485.38	5.33943
2485.75	5.33264
2486.12	5.27854
2486.49	5.24152
2486.86	5.16566
2487.23	4.89067
2487.6	4.7491
2487.97	4.6218
2488.34	4.46703
2488.71	4.3404
2489.08	4.21527
2489.45	4.04885
2489.82	4.02027
2490.19	4.03199
2490.56	4.05385
2490.93	4.07968
2491.3	4.4169
2491.67	4.53837
2492.04	4.74315

2492.41	4.89396
2492.78	5.04284
2493.15	5.10617
2493.52	5.08342
2493.89	5.04776
2494.25	5.02853
2494.62	4.81892
2494.99	4.31931
2495.36	3.91104
2495.73	3.4756
2496.1	3.0381
2496.47	2.66382
2496.84	2.2482
2497.21	1.77471
2497.58	1.65089
2497.95	1.56664
2498.32	1.68645
2498.68	1.74385
2499.05	1.81178
2499.42	1.89247
2499.79	1.96255
2500.16	2.04315
2500.53	2.10775
2500.9	2.18625
2501.27	2.23903
2501.64	2.30541
2502	2.33475
2502.37	2.31776
2502.74	2.2998
2503.11	2.27613
2503.48	2.26649
2503.85	2.24507
2504.22	2.09917
2504.58	2.14176
2504.95	2.18993
2505.32	2.23389
2505.69	2.28938
2506.06	2.33231
2506.43	2.38791
2506.79	2.42266
2507.16	2.47104
2507.53	2.5024
2507.9	2.53177
2508.27	2.55359
2508.64	2.56772
2509.01	2.57974
2509.37	2.57931
2509.74	2.58065
2510.11	2.56888
2510.48	2.56149
2510.85	2.53884
2511.21	2.52053
2511.58	2.48799
2511.95	2.45851
2512.32	2.41678
2512.69	2.36572
2513.05	2.20523
2513.42	1.89644
2513.79	1.58981
2514.16	1.25637
2514.53	0.868998
2514.89	0.679772

2515.26	0.67037
2515.63	0.79703
2516	1.56403
2516.36	1.28133
2516.73	1.00916
2517.1	0.764475
2517.47	0.506027
2517.84	0.303338
2518.2	0.0800586
2518.57	-0.0965872
2518.94	-0.28866
2519.31	-0.469765
2519.67	-0.630682
2520.04	-0.794899
2520.41	-0.917418
2520.78	-1.03545
2521.14	-1.09115
2521.51	-1.13512
2521.88	-1.09725
2522.24	-0.945842
2522.61	-0.810884
2522.98	-0.483966
2523.35	-0.145673
2523.71	0.251881
2524.08	0.645887
2524.45	1.0754
2524.81	1.4823
2525.18	1.87436
2525.55	2.15732
2525.91	2.42452
2526.28	2.24011
2526.65	1.92044
2527.02	1.61446
2527.38	1.24306
2527.75	0.812201
2528.12	0.364913
2528.48	-0.0740499
2528.85	-0.574208
2529.22	-0.901793
2529.36	-0.988057
2529.73	-0.899212
2530.1	-0.791982
2530.47	-0.607867
2530.84	-0.389596
2531.22	-0.203848
2531.59	0.345325
2531.96	0.361676
2532.33	0.366225
2532.7	0.360933
2533.07	0.317772
2533.44	0.142869
2533.81	0
2534.19	-0.190454
2534.56	-0.399028
2534.93	-0.585515
2535.3	-0.745769
2535.67	-0.920606
2536.04	-1.0702
2536.41	-1.19851
2536.78	-1.30729
2537.16	-1.41186
2537.53	-1.37994

2537.9	-1.42614
2538.27	-1.4172
2538.64	-1.36279
2539.01	-1.34762
2539.38	-1.30727
2539.75	-1.26636
2540.12	-1.21604
2540.49	-1.15768
2540.86	-1.12374
2541.24	-1.05253
2541.61	-0.972802
2541.98	-0.894003
2542.35	-0.803438
2542.72	-0.714496
2543.09	-0.612359
2543.46	-0.511537
2543.83	-0.40106
2544.2	-0.288574
2544.57	-0.178201
2544.94	-0.0586157
2545.31	0.152339
2545.68	0.219374
2546.05	0.251004
2546.42	0.295193
2546.79	0.299671
2547.17	0.283351
2547.54	0.0665903
2547.91	-0.050231
2548.28	-0.418551
2548.65	-0.605194
2549.02	-0.671065
2549.39	-0.714763
2549.76	-0.761837
2550.13	-0.786607
2550.5	-0.564941
2550.87	-0.476981
2551.24	-0.411271
2551.61	-0.342237
2551.98	-0.28104
2552.35	-0.232821
2552.72	-0.186807
2553.09	-0.14365
2553.46	-0.103373
2553.83	-0.0604391
2554.2	-0.0533075
2554.57	-0.0467796
2554.94	-0.0303431
2555.31	-0.00823116
2555.68	0
2556.04	-0.0920153
2556.41	2.36729
2556.78	4.58458
2557.15	6.80324
2557.52	7.28123
2557.89	7.02663
2558.26	6.73387
2558.63	6.48783
2559	6.20715
2559.37	5.94681
2559.74	5.75705
2560.11	5.52364
2560.48	5.38334

2560.85	5.27015
2561.22	5.65178
2561.59	5.96493
2561.96	6.29475
2562.33	6.65795
2562.69	7.01978
2563.06	7.35742
2563.43	7.70492
2563.8	8.03691
2564.17	8.31466
2564.54	8.55051
2564.91	8.53356
2565.28	8.45057
2565.65	8.06401
2566.02	7.68527
2566.39	7.21787
2566.76	6.71511
2567.12	6.18696
2567.49	5.6714
2567.86	5.16216
2568.23	4.69729
2568.6	4.20436
2568.97	4.28919
2569.34	4.35268
2569.71	4.52231
2570.07	4.74049
2570.44	4.99046
2570.81	5.26177
2571.18	5.52568
2571.55	5.77505
2571.92	5.99058
2572.29	6.18649
2572.66	5.99075
2573.02	5.74257
2573.39	5.44932
2573.76	5.10518
2574.13	4.73493
2574.5	4.37722
2574.87	4.00548
2575.23	3.67675
2575.6	3.29798
2575.97	3.19701
2576.34	3.38622
2576.71	3.54307
2577.08	3.75636
2577.44	3.99494
2577.81	4.18022
2578.18	4.70405
2578.55	4.71696
2578.92	4.67991
2579.28	4.59911
2579.65	4.37193
2580.02	4.17416
2580.39	3.95046
2580.76	3.72894
2581.12	3.50683
2581.49	3.30191
2581.86	3.09901
2582.23	2.8986
2582.6	2.72478
2582.96	2.53671
2583.33	2.40007

2583.7	2.32216
2584.07	2.2473
2584.43	2.18974
2584.8	2.11463
2585.17	2.03208
2585.54	1.97026
2585.91	1.89173
2586.27	1.83837
2586.64	1.76924
2587.01	1.71816
2587.37	1.63556
2587.74	1.60868
2588.11	1.4716
2588.48	1.49981
2588.84	1.50012
2589.21	1.5471
2589.58	1.58038
2589.95	1.70503
2590.31	1.83225
2590.68	1.96893
2591.05	2.13246
2591.42	2.2906
2591.78	2.42726
2592.15	2.81619
2592.52	2.83667
2592.88	2.83538
2593.25	2.82111
2593.62	2.78386
2593.98	2.47028
2594.35	2.25294
2594.72	2.10166
2595.09	1.87744
2595.45	1.71214
2595.82	1.53338
2596.19	1.36945
2596.55	1.21881
2596.92	1.06223
2597.29	1.09676
2597.65	1.00624
2598.02	0.972897
2598.39	0.951026
2598.75	0.920276
2599.12	0.889455
2599.49	0.876728
2599.85	0.856476
2600.22	0.857825
2600.59	0.84349
2600.95	0.8451
2601.32	0.821843
2601.69	0.794599
2602.05	0.809568
2602.42	0.890148
2602.79	0.969596
2603.15	1.06535
2603.52	1.16584
2603.88	1.27833
2604.25	1.40549
2604.62	1.53107
2604.98	1.66805
2605.35	1.80436
2605.72	1.94591
2606.08	2.08919

2606.45	2.23018
2606.81	2.37554
2607.18	2.51021
2607.55	2.64355
2607.91	2.76194
2608.28	2.86853
2608.64	2.96903
2609.01	3.04678
2609.38	2.90854
2609.74	2.8975
2610.11	2.93514
2610.47	2.9111
2610.84	2.9131
2611.21	2.88597
2611.57	2.89015
2611.94	2.8657
2612.3	2.86562
2612.67	2.83977
2613.03	2.83656
2613.4	2.84213
2613.77	2.79629
2614.13	2.82051
2614.5	2.74036
2614.86	2.61269
2615.23	2.49257
2615.59	2.33695
2615.96	2.18691
2616.32	2.00461
2616.69	1.8438
2617.05	1.65111
2617.42	1.4633
2617.79	1.26095
2618.15	1.09575
2618.52	1.07363
2618.88	1.39177
2619.25	1.62708
2619.61	1.89034
2619.98	2.10806
2620.34	2.40947
2620.71	2.65308
2621.07	2.87493
2621.44	3.05085
2621.8	3.14532
2622.17	3.06518
2622.53	2.70749
2622.9	2.3596
2623.26	1.95591
2623.63	1.57529
2623.99	1.13718
2624.36	0.721137
2624.72	0.35092
2625.09	0.0587752
2625.45	-0.166998
2625.82	-0.0861847
2626.18	0.189911
2626.54	0.577839
2626.91	1.01407
2627.28	1.47108
2627.64	1.93513
2628	2.38695
2628.37	2.80043
2628.73	3.17647

2629.1	3.30027
2629.46	3.39194
2629.83	3.32415
2630.19	3.006
2630.55	2.63585
2630.92	2.22468
2631.28	1.79307
2631.65	1.39532
2632.01	0.980081
2632.38	0.60582
2632.74	0.270449
2633.11	0.210256
2633.47	0.150521
2633.83	0.18737
2634.2	0.305019
2634.56	0.489113
2634.93	0.721577
2635.29	0.99459
2635.65	1.33169
2636.02	1.70057
2636.38	2.11815
2636.75	2.55377
2637.11	3.03455
2637.47	3.34397
2637.84	3.47828
2638.2	3.64724
2638.57	3.73815
2638.93	3.59415
2639.29	3.18624
2639.66	2.74487
2640.02	2.30814
2640.38	1.86737
2640.75	1.39384
2641.11	0.917531
2641.47	0.501307
2641.84	0.250703
2642.2	0.217026
2642.57	0.436821
2642.93	1.02535
2643.29	1.73031
2643.66	2.43778
2644.02	3.00309
2644.38	3.4695
2644.75	4.21228
2645.11	4.01342
2645.47	3.59157
2645.84	3.11427
2646.2	2.68077
2646.56	2.21576
2646.93	1.59044
2647.29	1.31172
2647.65	1.08404
2648.02	0.95036
2648.38	0.879907
2648.74	0.847554
2649.11	0.888614
2649.47	0.942333
2649.83	1.026
2650.19	1.11253
2650.56	1.22665
2650.92	1.33385
2651.28	1.46794

2651.65	1.58595
2652.01	1.7311
2652.37	1.85396
2652.73	2.00273
2653.1	2.12408
2653.46	2.26974
2653.82	2.38611
2654.19	2.45208
2654.55	2.54582
2654.91	2.63697
2655.27	2.71432
2655.64	2.78239
2656	2.83668
2656.36	2.92191
2656.72	2.94712
2657.09	3.05202
2657.45	2.95988
2657.81	2.89442
2658.17	2.76489
2658.54	2.63245
2658.9	2.5009
2659.26	2.33228
2659.62	2.15032
2659.99	1.98288
2660.35	1.77447
2660.71	1.56437
2661.07	1.38233
2661.44	1.19564
2661.8	0.946298
2662.16	0.885648
2662.52	0.858284
2662.88	1.22187
2663.25	1.41069
2663.61	1.56666
2663.97	1.77132
2664.33	1.98721
2664.69	2.20126
2665.06	2.36359
2665.42	2.54691
2665.78	2.7056
2666.14	2.82102
2666.5	2.93724
2666.87	2.7847
2667.23	2.51438
2667.59	2.22364
2667.95	1.87616
2668.31	1.51555
2668.67	1.14564
2669.04	0.788862
2669.4	0.377203
2669.76	0.0725658
2670.12	-0.247685
2670.48	-0.562929
2670.84	-0.902979
2671.2	-0.714235
2671.57	-0.440521
2671.93	-0.149511
2672.29	0.178281
2672.65	0.531513
2673.01	0.861873
2673.37	1.23547
2673.73	1.58613

2674.1	1.94459
2674.46	2.28566
2674.82	2.61393
2675.18	2.75787
2675.54	2.93428
2675.9	3.13219
2676.26	3.21446
2676.62	3.31446
2676.99	3.40059
2677.35	3.45444
2677.71	3.5179
2678.07	3.5366
2678.43	3.58189
2678.79	3.48219
2679.15	3.3913
2679.51	3.25664
2679.87	3.12405
2680.23	2.9628
2680.6	2.79396
2680.96	2.61705
2681.32	2.42699
2681.68	2.24802
2682.04	2.05653
2682.4	1.86806
2682.76	1.70275
2683.12	1.44745
2683.48	1.46428
2683.84	1.65592
2684.2	2.02581
2684.56	2.29602
2684.92	2.55357
2685.28	2.87544
2685.64	3.15927
2686	3.46763
2686.36	3.76795
2686.73	4.05461
2687.08	4.12125
2687.45	3.87166
2687.81	3.67827
2688.17	3.44553
2688.53	3.18543
2688.89	2.92493
2689.25	2.64196
2689.61	2.34384
2689.97	2.0631
2690.33	1.75717
2690.69	1.49632
2691.05	1.21246
2691.41	1.1069
2691.77	0.9715
2692.13	0.923303
2692.49	0.851241
2692.85	0.772993
2693.21	0.733529
2693.57	0.699346
2693.93	0.667034
2694.29	0.640601
2694.65	0.612096
2695.01	0.573253
2695.37	0.529824
2695.73	0.268096
2696.09	0.028466

2696.45	-0.222203
2696.81	-0.174797
2697.17	0.166693
2697.53	0.742135
2697.88	1.48419
2698.24	2.35782
2698.6	3.26897
2698.96	4.0436
2699.32	4.71239
2699.68	5.53381
2700.04	5.26072
2700.4	4.58089
2700.76	3.8651
2701.12	3.37703
2701.48	2.74861
2701.84	2.53929
2702.2	2.39978
2702.56	2.30318
2702.92	2.26904
2703.28	2.24915
2703.64	2.27797
2703.99	2.30041
2704.35	2.35737
2704.71	2.39143
2705.07	2.43875
2705.43	2.45769
2705.79	2.44489
2706.15	2.43055
2706.51	2.26299
2706.87	2.10949
2707.23	1.99371
2707.58	1.78677
2707.94	1.6296
2708.3	1.46902
2708.66	1.31129
2709.02	1.16981
2709.38	1.04477
2709.74	0.96251
2710.1	0.920136
2710.45	1.33512
2710.81	1.6359
2711.17	2.06889
2711.53	2.51397
2711.89	2.97784
2712.25	3.42682
2712.61	3.8737
2712.97	4.30501
2713.32	4.70732
2713.68	5.11015
2714.04	5.46016
2714.4	5.73246
2714.76	5.79144
2715.11	5.71385
2715.47	5.5033
2715.83	5.1338
2716.19	4.59901
2716.55	3.96608
2716.91	3.31705
2717.26	2.6541
2717.62	1.99646
2717.98	1.34622
2718.34	0.742678

2718.7	0.194517
2719.06	0.348308
2719.41	0.533713
2719.77	0.833359
2720.13	1.20993
2720.49	1.6386
2720.85	2.12536
2721.2	2.64797
2721.56	3.18397
2721.92	3.61873
2722.28	3.88189
2722.64	4.01237
2722.99	4.04208
2723.35	3.96272
2723.71	3.78394
2724.07	3.42052
2724.42	3.00528
2724.78	2.69788
2725.14	2.44199
2725.5	2.24462
2725.85	2.03878
2726.21	1.96686
2726.57	2.15305
2726.93	2.60103
2727.28	3.16547
2727.64	3.78979
2728	4.40211
2728.36	4.95458
2728.71	5.80041
2729.07	5.61383
2729.43	5.14954
2729.79	4.65048
2730.14	4.15791
2730.5	3.70632
2730.86	3.29329
2731.21	2.71886
2731.57	2.55808
2731.93	2.4429
2732.29	2.30422
2732.64	2.26657
2733	2.23291
2733.36	2.24097
2733.71	2.2548
2734.07	2.29153
2734.43	2.33807
2734.79	2.39573
2735.14	2.45845
2735.5	2.52134
2735.86	2.59236
2736.21	2.65623
2736.57	2.72973
2736.93	2.79371
2737.28	2.86632
2737.64	2.90066
2738	2.93631
2738.35	2.98044
2738.71	3.01287
2739.07	3.055
2739.42	3.09456
2739.78	3.13498
2740.14	3.18343
2740.49	3.2122

2740.85	3.25708
2741.21	3.27234
2741.56	3.31009
2741.92	3.30941
2742.27	3.52818
2742.63	3.45715
2742.99	3.39341
2743.34	3.31662
2743.7	3.2324
2744.06	3.151
2744.41	3.04737
2744.77	2.96335
2745.13	2.8179
2745.48	2.6926
2745.84	2.60626
2746.19	2.49069
2746.55	2.41055
2746.91	2.31211
2747.26	2.23796
2747.62	2.16572
2747.97	2.11245
2748.33	2.06772
2748.69	2.03617
2749.04	2.18929
2749.4	2.21521
2749.75	2.2832
2750.11	2.3349
2750.47	2.48151
2750.82	2.57099
2751.18	2.69471
2751.53	2.72303
2751.89	2.88385
2752.24	3.08128
2752.6	3.26808
2752.96	3.46982
2753.31	3.67095
2753.67	3.86853
2754.02	4.07029
2754.38	4.26996
2754.73	4.46065
2755.09	4.61019
2755.45	4.76049
2755.8	4.89384
2756.16	4.978
2756.51	5.10332
2756.87	4.89142
2757.22	4.46877
2757.58	4.14345
2757.93	3.79709
2758.29	3.39245
2758.64	3.0383
2759	2.67885
2759.35	2.32343
2759.71	1.98241
2760.06	1.66939
2760.42	1.42512
2760.77	1.43512
2761.13	1.36778
2761.48	1.6686
2761.84	1.97604
2762.19	2.3368
2762.55	2.72484

2762.9	3.07909
2763.26	3.48024
2763.61	3.82494
2763.97	4.17418
2764.32	4.47221
2764.68	4.38211
2765.03	4.33487
2765.39	4.20033
2765.74	3.99328
2766.1	3.72585
2766.45	3.43043
2766.81	3.08635
2767.16	2.71755
2767.52	2.29939
2767.87	1.88007
2768.22	1.4269
2768.58	1.46567
2768.93	1.26729
2769.29	1.18261
2769.64	1.95859
2770	2.84671
2770.35	3.85864
2770.71	4.6385
2771.06	4.72475
2771.41	2.53896
2771.77	2.45367
2772.12	2.36263
2772.48	2.2732
2772.83	2.18771
2773.18	2.10558
2773.54	2.02781
2773.89	1.95644
2774.25	1.88767
2774.6	1.83179
2774.96	1.77424
2775.31	1.7414
2775.66	1.70116
2776.02	1.69999
2776.37	1.68709
2776.73	1.71843
2777.08	1.73868
2777.43	1.79282
2777.79	1.84085
2778.14	1.90451
2778.5	1.96854
2778.85	2.02944
2779.2	2.09405
2779.56	2.13983
2779.91	2.18788
2780.26	2.20509
2780.62	2.22044
2780.97	1.84592
2781.32	1.74308
2781.68	1.55398
2782.03	1.34223
2782.39	1.13857
2782.74	1.07516
2783.09	1.33109
2783.45	1.67411
2783.8	2.07506
2784.15	2.37017
2784.51	2.54706

2784.86	2.67254
2785.21	2.74315
2785.57	2.78066
2785.92	2.78388
2786.27	2.76889
2786.63	2.69523
2786.98	2.55246
2787.33	2.2464
2787.69	1.4445
2788.04	0.333376
2788.39	-0.271553
2788.74	-0.202487
2789.1	-0.0965858
2789.45	0.647191
2789.8	2.64196
2790.16	3.02046
2790.51	3.1418
2790.86	3.08958
2791.21	2.99853
2791.57	2.85532
2791.92	2.68057
2792.27	2.52585
2792.63	2.33738
2792.98	2.18648
2793.33	2.00816
2793.68	1.87445
2794.04	1.72406
2794.39	1.62353
2794.74	1.51406
2795.09	1.46392
2795.45	1.41346
2795.8	1.40621
2796.15	1.45897
2796.51	1.62353
2796.86	1.85498
2797.21	2.10041
2797.56	2.36994
2797.92	2.64721
2798.27	2.91835
2798.62	3.20473
2798.97	3.46457
2799.32	3.72093
2799.68	3.87646
2800.03	3.55781
2800.38	3.05465
2800.73	2.62073
2801.09	2.11361
2801.44	1.6087
2801.79	1.07442
2802.14	0.547081
2802.49	0.048656
2802.85	-0.381728
2803.2	-0.385424
2803.55	-0.392356
2803.9	-0.275709
2804.25	-0.0340476
2804.61	0.391806
2804.96	0.976334
2805.31	1.68562
2805.66	2.49513
2806.01	3.29255
2806.37	4.13474

2806.72	4.64266
2807.07	4.75342
2807.42	4.7616
2807.77	4.74949
2808.12	4.7018
2808.48	4.54781
2808.83	4.22095
2809.18	3.54535
2809.53	2.52154
2809.88	1.62478
2810.23	0.191143
2810.58	0.0238066
2810.94	0.731636
2811.29	1.62676
2811.64	2.58243
2811.99	3.22393
2812.34	3.76078
2812.69	4.23399
2813.04	4.53537
2813.4	4.44861
2813.75	4.27954
2814.1	4.05754
2814.45	3.77914
2814.8	3.47851
2815.15	3.08943
2815.5	2.7852
2815.85	2.46877
2816.21	2.09233
2816.56	1.92122
2816.91	1.8471
2817.26	1.73385
2817.61	1.76133
2817.96	1.77288
2818.31	1.76874
2818.66	1.86225
2819.01	1.87114
2819.36	1.90992
2819.72	2.05897
2820.07	1.97002
2820.42	1.82903
2820.77	1.7166
2821.12	1.37378
2821.47	1.19297
2821.82	0.952819
2822.17	0.790599
2822.52	0.565081
2822.87	0.366697
2823.22	0.313581
2823.57	0.0817728
2823.92	0.238644
2824.27	0.593208
2824.62	0.927444
2824.97	1.24713
2825.32	1.67702
2825.68	2.05399
2826.03	2.41869
2826.38	2.72476
2826.73	3.04271
2827.08	3.28675
2827.43	3.82765
2827.78	3.8823
2828.13	3.8431

2828.48	3.70189
2828.83	3.59634
2829.18	3.50362
2829.53	3.34538
2829.88	3.22439
2830.23	3.08764
2830.58	2.96102
2830.93	2.83217
2831.28	2.70768
2831.63	2.59685
2831.98	2.48446
2832.33	2.37314
2832.68	2.26238
2833.03	2.16387
2833.38	2.02125
2833.73	1.91421
2834.08	1.81027
2834.43	1.68004
2834.78	1.54342
2835.13	1.41234
2835.48	1.29984
2835.83	1.19908
2836.17	1.11946
2836.52	1.15614
2836.88	1.22896
2837.22	1.31687
2837.57	1.4186
2837.92	1.55853
2838.27	1.71076
2838.62	1.841
2838.97	1.9539
2839.32	2.05455
2839.67	2.0836
2840.02	2.064
2840.37	1.99595
2840.72	1.90563
2841.07	1.7876
2841.42	1.66537
2841.77	1.5315
2842.12	1.38787
2842.47	1.24605
2842.81	1.05353
2843.16	0.989799
2843.51	1.04556
2843.86	1.0688
2844.21	1.12722
2844.56	1.20098
2844.91	1.35826
2845.26	1.44674
2845.61	1.51902
2845.95	1.58277
2846.3	1.64238
2846.65	1.7023
2847	1.74252
2847.35	1.74244
2847.7	1.68918
2848.05	1.64823
2848.4	1.59028
2848.75	1.54422
2849.09	1.48591
2849.44	1.44412
2849.79	1.38768

2850.14	1.35286
2850.49	1.30788
2850.84	1.27825
2851.19	1.24141
2851.54	1.21043
2851.88	1.18168
2852.23	1.15789
2852.58	1.1369
2852.93	1.16317
2853.28	1.13396
2853.62	1.02148
2853.97	0.887001
2854.32	0.741675
2854.67	0.578493
2855.02	0.401877
2855.37	0.211793
2855.71	0.0246296
2856.06	-0.174762
2856.41	-0.3347
2856.76	-0.397636
2857.11	-0.294089
2857.46	-0.0123482
2857.8	0.174742
2858.15	0.449237
2858.5	0.744738
2858.85	1.00579
2859.2	1.29702
2859.54	1.32468
2859.89	1.28338
2860.24	1.10446
2860.59	0.923607
2860.94	0.713223
2861.28	0.475751
2861.63	0.259179
2861.98	0.031703
2862.33	-0.181141
2862.62	-0.341042
2862.97	-0.429377
2863.33	-0.324873
2863.68	-0.272397
2864.03	-0.218194
2864.38	-0.0892625
2864.73	0.0225725
2865.09	0.136274
2865.44	0.257514
2865.79	0.383948
2866.14	0.499932
2866.5	0.625336
2866.85	0.899975
2867.2	0.87385
2867.55	0.854689
2867.9	0.786707
2868.26	0.719833
2868.61	0.359634
2868.96	0.115785
2869.31	-0.202556
2869.66	-0.287913
2870.02	-0.320705
2870.37	-0.342954
2870.72	-0.319208
2871.07	-0.114179
2871.42	0.00662947

2871.78	0.109039
2872.13	0.21526
2872.48	0.319837
2872.83	0.427224
2873.18	0.544194
2873.53	0.644141
2873.89	0.7629
2874.24	0.864843
2874.59	0.9941
2874.94	1.00466
2875.29	0.991954
2875.64	0.767283
2876	0.609099
2876.35	0.399232
2876.7	0.231018
2877.05	0.00387907
2877.4	-0.221871
2877.75	-0.41248
2878.1	-0.61621
2878.45	-0.775666
2878.81	-0.923272
2879.16	-1.0315
2879.51	-1.05925
2879.86	-0.573566
2880.21	-0.244503
2880.56	0.162971
2880.91	0.607287
2881.26	1.14319
2881.61	1.65165
2881.97	1.87271
2882.32	1.90341
2882.67	1.40919
2883.02	0.878123
2883.37	0.444132
2883.72	0.490942
2884.07	0.748559
2884.42	1.34111
2884.77	3.85794
2885.13	3.83912
2885.48	3.48909
2885.83	3.39375
2886.18	3.28313
2886.53	3.23917
2886.88	3.17397
2887.23	3.14707
2887.58	3.10717
2887.93	3.08712
2888.28	3.05742
2888.63	3.03561
2888.98	3.00969
2889.33	2.98554
2889.68	2.98286
2890.03	2.97136
2890.39	2.90504
2890.74	2.87747
2891.09	2.82154
2891.44	2.67268
2891.79	2.56397
2892.14	2.36201
2892.49	2.19458
2892.84	1.96783
2893.19	1.72482

2893.54	1.50296
2893.89	1.28944
2894.24	1.08364
2894.59	0.937759
2894.94	0.813342
2895.29	0.721648
2895.64	1.04068
2895.99	1.17721
2896.34	1.38646
2896.69	1.5776
2897.04	1.45828
2897.39	1.15693
2897.74	0.848384
2898.09	0.410621
2898.44	-0.005373
2898.79	-0.390841
2899.14	-0.801666
2899.49	-1.0157
2899.84	-1.16079
2900.19	-0.845911
2900.54	-0.359303
2900.89	0.210623
2901.24	0.819677
2901.59	1.49423
2901.94	2.01164
2902.29	2.30039
2902.64	2.78479
2902.99	2.77069
2903.34	2.73391
2903.69	2.56898
2904.03	2.32314
2904.38	2.08105
2904.73	1.65027
2905.08	1.59111
2905.43	1.52134
2905.78	1.50144
2906.13	1.592
2906.48	1.69518
2906.83	1.81485
2907.18	2.04905
2907.53	2.17434
2907.88	2.33978
2908.23	2.43947
2908.58	2.51122
2908.93	2.54457
2909.28	2.56295
2909.62	2.56871
2909.97	2.52911
2910.32	2.38642
2910.67	2.26311
2911.02	2.12966
2911.37	1.98018
2911.72	1.86592
2912.07	1.73153
2912.42	1.61102
2912.77	1.50068
2913.12	1.37986
2913.46	1.30662
2913.81	1.18556
2914.16	1.10472
2914.51	0.985914
2914.86	0.894487

2915.21	0.785524
2915.56	0.744107
2915.9	0.725065
2916.25	0.6736
2916.6	0.682454
2916.95	0.706486
2917.3	0.766101
2917.65	0.836035
2918	0.928561
2918.34	0.989062
2918.69	0.140779
2919.04	0.287115
2919.39	0.662691
2919.74	1.10407
2920.09	1.71267
2920.44	2.29906
2920.78	2.82299
2921.13	3.22319
2921.48	3.54389
2921.83	3.83673
2922.18	3.36826
2922.53	2.79532
2922.87	1.99981
2923.22	0.935308
2923.57	0.731117
2923.92	0.586323
2924.27	0.566945
2924.61	0.577551
2924.96	0.643016
2925.31	0.756724
2925.66	0.872203
2926.01	1.05937
2926.35	1.17945
2926.7	1.37376
2927.05	1.46226
2927.4	1.45521
2927.75	1.45144
2928.09	1.50969
2928.44	1.52859
2928.79	1.57779
2929.14	1.62716
2929.49	1.7516
2929.83	1.90185
2930.18	2.08934
2930.53	2.37136
2930.88	2.70392
2931.22	3.0845
2931.57	3.41677
2931.92	3.89782
2932.27	3.51541
2932.61	3.01977
2932.96	2.47053
2933.31	1.9034
2933.66	1.53513
2934	1.28436
2934.35	1.10851
2934.7	1.34768
2935.05	1.67602
2935.39	2.07481
2935.74	2.52701
2936.09	3.03164
2936.43	3.66224

2936.78	4.06531
2937.13	4.4229
2937.48	4.68495
2937.82	4.65581
2938.17	4.58092
2938.52	4.45667
2938.86	4.26734
2939.21	4.07819
2939.56	3.82938
2939.91	3.70348
2940.25	3.48239
2940.6	3.18725
2940.95	2.90281
2941.29	2.62539
2941.64	2.36148
2941.99	2.0998
2942.33	1.85359
2942.68	1.65106
2943.03	1.46735
2943.37	1.30083
2943.72	1.2306
2944.07	1.18599
2944.41	1.18588
2944.76	1.19142
2945.11	1.28791
2945.45	1.3936
2945.8	1.56447
2946.15	1.62849
2946.49	1.91438
2946.84	2.18867
2947.19	2.50592
2947.53	2.81412
2947.88	3.13021
2948.23	3.44317
2948.57	3.74532
2948.92	3.99318
2949.27	4.21088
2949.61	4.41215
2949.96	4.37902
2950.3	4.38105
2950.65	4.09472
2951	3.79404
2951.34	3.36819
2951.69	2.93901
2952.04	2.46316
2952.38	1.99336
2952.73	1.56583
2953.07	0.988454
2953.42	0.6709
2953.77	0.390273
2954.11	0.403386
2954.46	0.493299
2954.8	0.521151
2955.15	0.691348
2955.5	0.709772
2955.84	0.755667
2956.19	0.769238
2956.53	0.822388
2956.88	0.826663
2957.22	0.86382
2957.57	0.685061
2957.92	0.638608

2958.26	0.537135
2958.61	0.233588
2958.95	0.415305
2959.3	0.626531
2959.65	0.85922
2959.99	1.08561
2960.34	1.35667
2960.68	1.68551
2961.03	2.00215
2961.37	2.26612
2961.72	2.56461
2962.06	2.81572
2962.41	3.04154
2962.75	3.2672
2963.1	3.30149
2963.44	3.38858
2963.79	3.47934
2964.13	3.49927
2964.48	3.53056
2964.83	3.55333
2965.17	3.56547
2965.52	3.55722
2965.86	3.52664
2966.21	3.48264
2966.55	3.40724
2966.9	3.37246
2967.24	3.37754
2967.59	3.14245
2967.93	2.91724
2968.28	2.65954
2968.62	2.3814
2968.97	2.11414
2969.31	1.823
2969.66	1.54158
2970	1.23561
2970.35	0.989282
2970.69	0.811521
2971.04	0.620131
2971.38	0.442604
2971.73	0.747975
2972.07	1.06188
2972.42	1.30507
2972.76	1.61871
2973.1	1.9565
2973.45	2.29159
2973.79	2.63942
2974.14	2.95831
2974.48	3.23354
2974.83	3.51283
2975.17	3.455
2975.52	3.56602
2975.86	3.41569
2976.2	3.30315
2976.55	3.0903
2976.89	2.81685
2977.24	2.56034
2977.58	2.3348
2977.93	2.08496
2978.27	1.85039
2978.62	1.67859
2978.96	1.49362
2979.3	1.37728

2979.65	1.4838
2979.99	1.54826
2980.34	1.68322
2980.68	1.88626
2981.02	2.0866
2981.37	2.49065
2981.71	2.91375
2982.06	3.39823
2982.4	3.84152
2982.75	4.30439
2983.09	4.71594
2983.43	5.16996
2983.78	5.48076
2984.12	5.64038
2984.46	5.69079
2984.81	5.57335
2985.15	5.05879
2985.5	4.49248
2985.84	3.879
2986.18	3.27733
2986.53	2.6769
2986.87	2.12318
2987.21	1.56435
2987.56	1.05692
2987.9	0.958878
2988.24	0.811002
2988.59	0.829117
2988.93	1.04185
2989.28	1.3867
2989.62	1.7872
2989.96	2.24167
2990.31	2.69807
2990.65	3.1765
2990.99	3.66784
2991.34	4.11863
2991.68	4.58648
2992.02	4.9699
2992.37	5.02908
2992.71	5.14197
2993.05	5.25834
2993.4	5.23649
2993.74	5.127
2994.08	4.97691
2994.42	4.77114
2994.77	4.53042
2995.11	4.24288
2995.45	3.83713
2995.8	3.36474
2996.14	2.85275
2996.48	2.33103
2996.83	2.45772
2997.17	2.62588
2997.51	2.54789
2997.86	2.42987
2998.2	2.05664
2998.54	1.67508
2998.88	1.40555
2999.23	1.33231
2999.57	1.73485
2999.91	2.26819
3000.25	3.18945
3000.6	2.87822

3000.94	2.25415
3001.28	1.84319
3001.63	1.43741
3001.97	1.0955
3002.31	0.78079
3002.65	0.494761
3003	0.261154
3003.34	0.0378551
3003.68	-0.13493
3004.02	-0.278744
3004.37	-0.353002
3004.71	-0.412141
3005.05	-0.436391
3005.39	-0.152093
3005.74	0.124625
3006.08	0.480711
3006.42	0.826595
3006.76	1.21523
3007.1	1.60449
3007.45	1.98872
3007.79	2.38236
3008.13	2.78475
3008.47	3.13297
3008.81	3.48172
3009.16	3.43903
3009.5	3.45359
3009.84	3.39609
3010.18	3.31745
3010.53	3.19035
3010.87	3.06317
3011.21	2.89298
3011.55	2.68502
3011.89	2.48799
3012.23	2.29856
3012.58	2.12993
3012.92	2.01019
3013.26	1.91199
3013.6	2.18144
3013.94	2.30595
3014.29	2.41189
3014.63	2.57096
3014.97	2.73489
3015.31	2.88004
3015.65	2.98967
3016	3.14293
3016.34	3.27919
3016.68	3.11667
3017.02	2.94627
3017.36	2.41306
3017.7	1.97707
3018.04	1.53389
3018.38	0.941258
3018.73	0.473116
3019.07	0.488266
3019.41	0.547335
3019.75	0.701535
3020.09	0.877346
3020.43	1.14624
3020.77	1.44367
3021.12	1.74437
3021.46	2.05903
3021.8	2.43408

3022.14	2.6091
3022.48	2.81943
3022.82	2.74942
3023.16	2.81881
3023.5	2.7895
3023.84	2.77567
3024.19	2.71266
3024.53	2.60523
3024.87	2.49341
3025.21	2.34912
3025.55	2.50337
3025.89	2.6628
3026.23	2.99731
3026.57	3.29467
3026.91	3.61393
3027.25	3.94172
3027.59	4.25407
3027.94	4.51827
3028.28	4.70693
3028.62	4.88654
3028.96	4.80788
3029.3	4.78039
3029.64	4.55913
3029.98	4.38859
3030.32	3.94619
3030.66	3.44941
3031	2.9215
3031.34	2.39823
3031.68	1.84677
3032.02	1.33058
3032.36	0.901705
3032.7	0.461667
3033.04	0.272182
3033.38	0.0955129
3033.72	0.270514
3034.07	0.459155
3034.41	0.727187
3034.75	1.02019
3035.09	1.32771
3035.43	1.64553
3035.77	1.98599
3036.11	2.32846
3036.45	2.60916
3036.79	2.91992
3037.13	2.81827
3037.47	2.73548
3037.81	2.4776
3038.15	2.22824
3038.49	1.98543
3038.83	1.71682
3039.17	1.42319
3039.51	1.04352
3039.85	0.983396
3040.19	0.913354
3040.53	1.16444
3040.87	1.37553
3041.21	1.64048
3041.55	1.85157
3041.89	2.32349
3042.23	2.34752
3042.57	2.32093
3042.91	2.30448

3043.24	2.06663
3043.58	1.70057
3043.92	1.49551
3044.26	1.3785
3044.6	1.22357
3044.94	1.16641
3045.28	1.1474
3045.62	1.14852
3045.96	1.33276
3046.3	1.52623
3046.64	1.69903
3046.98	1.84646
3047.32	2.12067
3047.66	2.43261
3048	2.72761
3048.34	3.03403
3048.68	3.32907
3049.02	3.62167
3049.35	3.90805
3049.69	4.17145
3050.03	4.38471
3050.37	4.60582
3050.71	4.78021
3051.05	4.91523
3051.39	4.95054
3051.73	4.97114
3052.07	4.78142
3052.41	4.38519
3052.75	4.07454
3053.08	3.68567
3053.43	3.26632
3053.76	2.84903
3054.1	2.41241
3054.44	2.02834
3054.78	1.56274
3055.12	0.950211
3055.46	0.868863
3055.8	1.02953
3056.14	1.15205
3056.47	1.3135
3056.81	1.50845
3057.15	1.72144
3057.49	1.92436
3057.83	2.12985
3058.17	2.3277
3058.51	2.5362
3058.84	2.72371
3059.18	2.92335
3059.52	3.09168
3059.86	3.0292
3060.2	3.13151
3060.54	3.18328
3060.88	3.25495
3061.21	3.31618
3061.55	3.38918
3061.89	3.4495
3062.23	3.51656
3062.57	3.59891
3062.91	3.66921
3063.24	3.67345
3063.58	3.71514
3063.92	3.69485

3064.26	3.77096
3064.6	3.70256
3064.93	3.64411
3065.27	3.56392
3065.61	3.4688
3065.95	3.38397
3066.29	3.26355
3066.63	3.15788
3066.96	3.01465
3067.3	2.94175
3067.64	2.89269
3067.98	2.82405
3068.31	2.87416
3068.65	2.93928
3068.99	3.02794
3069.33	3.18527
3069.67	3.70298
3070	3.92632
3070.34	4.18519
3070.68	4.46415
3071.02	4.63233
3071.35	4.70323
3071.69	4.76571
3072.03	4.44923
3072.37	4.19263
3072.71	3.87992
3073.04	3.5959
3073.38	3.25559
3073.72	2.90967
3074.06	2.5673
3074.39	2.23241
3074.73	1.91645
3075.07	1.61835
3075.4	1.32053
3075.74	1.07715
3076.08	1.01163
3076.42	0.908427
3076.75	0.842638
3077.09	0.815712
3077.43	0.844227
3077.77	0.896815
3078.1	0.853148
3078.44	1.08208
3078.78	1.38443
3079.11	1.71288
3079.45	2.04011
3079.79	2.41199
3080.13	2.75105
3080.46	3.09569
3080.8	3.43323
3081.14	3.72339
3081.47	4.05005
3081.81	4.3105
3082.15	4.27416
3082.48	4.42377
3082.82	4.27373
3083.16	4.11194
3083.49	3.86798
3083.83	3.63535
3084.17	3.42088
3084.5	3.00702
3084.84	2.80016

3085.18	2.60173
3085.51	2.41245
3085.85	2.24974
3086.19	2.10906
3086.52	1.99288
3086.86	1.97012
3087.2	1.9419
3087.53	2.00294
3087.87	2.10458
3088.21	2.13853
3088.54	2.23134
3088.88	2.3165
3089.22	2.39895
3089.55	2.45898
3089.89	2.54188
3090.23	2.59992
3090.56	2.67192
3090.9	2.73298
3091.23	2.79145
3091.57	2.66689
3091.91	2.73052
3092.24	2.8043
3092.58	2.86841
3092.92	2.9491
3093.25	3.01979
3093.59	3.09079
3093.92	3.1861
3094.26	3.22213
3094.6	3.27574
3094.93	3.43486
3095.27	3.31406
3095.6	3.17858
3095.94	3.00501
3096.28	2.80489
3096.61	2.60298
3096.95	2.12526
3097.28	1.80381
3097.62	1.44417
3097.95	1.23903
3098.29	1.01135
3098.63	1.01556
3098.96	1.24553
3099.3	1.38533
3099.63	1.64939
3099.97	1.8937
3100.3	2.12534
3100.64	2.3864
3100.98	2.60217
3101.31	2.82443
3101.65	3.03465
3101.98	3.175
3102.32	3.3231
3102.65	3.1352
3102.99	2.88503
3103.32	2.61675
3103.66	2.34803
3103.99	2.00303
3104.33	1.67434
3104.67	1.33942
3105	1.04374
3105.34	0.804908
3105.67	0.514263

3106.01	0.786468
3106.34	1.15478
3106.68	1.54954
3107.01	2.00581
3107.35	2.48744
3107.68	2.87932
3108.02	3.38131
3108.35	3.78783
3108.69	3.99158
3109.02	3.87007
3109.36	3.78604
3109.69	3.6466
3110.03	3.49736
3110.36	3.2927
3110.7	3.05528
3111.03	2.88354
3111.37	2.81933
3111.7	2.77258
3112.04	2.75884
3112.37	2.71791
3112.71	2.91369
3113.04	3.08771
3113.38	3.1632
3113.71	3.23041
3114.04	3.32924
3114.38	3.24786
3114.71	3.02198
3115.05	2.82013
3115.38	2.35147
3115.72	2.04031
3116.05	1.69259
3116.39	1.34353
3116.72	1.04996
3117.05	0.73465
3117.39	0.425655
3117.72	0.0832458
3118.06	0.122114
3118.39	0.382717
3118.73	0.622576
3119.06	0.911027
3119.39	1.2332
3119.73	1.57385
3120.06	1.87207
3120.4	2.22778
3120.73	2.51716
3121.07	3.00993
3121.4	3.32272
3121.73	3.36392
3122.07	3.13759
3122.4	2.97899
3122.74	2.84209
3123.07	2.64777
3123.4	2.43619
3123.74	2.25364
3124.07	2.0437
3124.41	1.85684
3124.74	1.66865
3125.07	1.51692
3125.41	1.41267
3125.74	1.31821
3126.07	1.24471
3126.41	1.28681

3126.74	1.39682
3127.08	1.43447
3127.41	1.36505
3127.74	1.5501
3128.08	1.77384
3128.41	1.98637
3128.74	2.23397
3129.08	2.47601
3129.41	2.74264
3129.75	3.03061
3130.08	3.30342
3130.41	3.55289
3130.74	3.81672
3131.08	4.06566
3131.41	4.274
3131.75	4.48524
3132.08	4.68567
3132.41	4.83202
3132.74	4.97339
3133.08	4.69425
3133.41	4.56949
3133.75	4.47204
3134.08	4.38962
3134.41	4.29955
3134.74	4.21481
3135.08	4.13618
3135.41	4.0462
3135.74	3.97016
3136.08	3.88249
3136.41	3.80791
3136.74	3.768
3137.08	3.54438
3137.41	3.6306
3137.74	3.76593
3138.07	4.08195
3138.41	4.38519
3138.74	4.68907
3139.07	5.22176
3139.41	5.44637
3139.74	5.6526
3140.07	5.83905
3140.4	6.00787
3140.74	6.14323
3141.07	6.23667
3141.4	6.28522
3141.74	6.32458
3142.07	6.32515
3142.4	6.34265
3142.73	6.33634
3143.07	6.33873
3143.4	6.31597
3143.73	6.30833
3144.06	6.28556
3144.39	6.19278
3144.73	6.05548
3145.06	5.90323
3145.39	5.79226
3145.73	5.66313
3146.06	5.56407
3146.39	5.52262
3146.72	5.49258
3147.05	5.43081

3147.39	5.38456
3147.72	5.34094
3148.05	5.29798
3148.38	5.23008
3148.71	5.16002
3149.05	5.07584
3149.38	5.00608
3149.71	4.93643
3150.04	4.87429
3150.38	4.83549
3150.71	4.71432
3151.04	4.67273
3151.37	4.63415
3151.7	4.6076
3152.04	4.58272
3152.37	4.53794
3152.7	4.516
3153.03	4.52066
3153.36	4.5795
3153.69	4.5803
3154.03	4.55705
3154.36	4.47693
3154.69	4.27639
3155.02	3.98986
3155.35	3.71328
3155.68	3.40607
3156.02	2.92093
3156.35	2.62686
3156.68	2.50639
3157.01	2.4191
3157.34	2.36989
3157.67	2.9196
3158	3.45772
3158.34	4.10251
3158.67	4.52989
3159	4.81308
3159.33	5.05439
3159.66	5.28165
3159.99	5.45133
3160.32	5.39301
3160.66	5.15869
3160.99	4.82597
3161.32	4.55437
3161.65	4.15934
3161.98	3.77668
3162.31	3.44248
3162.64	3.09416
3162.97	2.82584
3163.31	2.56364
3163.64	2.34083
3163.97	2.14322
3164.3	2.13025
3164.63	2.07732
3164.96	1.96648
3165.29	1.94133
3165.62	1.95893
3165.95	2.05209
3166.28	2.18741
3166.62	2.33101
3166.95	2.32291
3167.28	2.28036
3167.61	2.21617

3167.94	2.16005
3168.27	2.07035
3168.6	1.99556
3168.93	1.94674
3169.26	1.89081
3169.59	1.76567
3169.92	1.60681
3170.25	1.58357
3170.58	1.52689
3170.91	1.48934
3171.25	1.30562
3171.58	1.01797
3171.91	0.682006
3172.24	0.51679
3172.57	0.60285
3172.9	0.647997
3173.23	0.81606
3173.56	0.854583
3173.89	0.902578
3174.22	1.03768
3174.55	1.19828
3174.88	1.29808
3175.21	1.18457
3175.54	1.08158
3175.87	1.00372
3176.2	0.866609
3176.53	0.722101
3176.61	0.66046
3176.94	0.824051
3177.28	0.716266
3177.61	0.732842
3177.95	0.531064
3178.28	0.363863
3178.61	0.414716
3178.95	0.619608
3179.28	1.10921
3179.62	1.47463
3179.95	1.79429
3180.29	2.10287
3180.62	2.3328
3180.96	2.54593
3181.29	2.68033
3181.63	2.65753
3181.96	2.51043
3182.29	2.39021
3182.63	2.20419
3182.96	2.09648
3183.3	2.10339
3183.63	2.33522
3183.97	2.34699
3184.3	2.37692
3184.63	2.55701
3184.97	2.62458
3185.3	2.66513
3185.64	2.75857
3185.97	2.78736
3186.31	2.71483
3186.64	2.64781
3186.97	2.45764
3187.31	2.24843
3187.64	1.98452
3187.98	1.6708

3188.31	1.29338
3188.64	0.779234
3188.98	0.312585
3189.31	-0.0884254
3189.64	-0.544344
3189.98	-0.721988
3190.31	-0.754622
3190.65	-0.498695
3190.98	-0.288854
3191.31	-0.0622184
3191.65	0.184152
3191.98	0.429787
3192.32	0.710239
3192.65	0.822525
3192.98	0.831635
3193.32	0.742902
3193.65	0.620126
3193.98	0.346708
3194.32	0.109723
3194.65	-0.222186
3194.98	-0.55642
3195.32	-0.934902
3195.65	-1.25551
3195.98	-1.39232
3196.32	-1.57229
3196.65	-1.58749
3196.98	-1.50574
3197.32	-1.31986
3197.65	-1.21767
3197.98	-0.927249
3198.32	-0.698481
3198.65	-0.460001
3198.98	0
3199.32	0.194493
3199.65	0.274278
3199.98	0.517065

Table S2. XRD data

2θ	Counts
9.41989	4.02279
9.43979	1.40495
9.45968	2.83919
9.47958	2.53385
9.49947	0.67643
9.51937	1.44401
9.53926	1.83138
9.55916	4.14063
9.57905	1.29362
9.59895	4.05078
9.61884	2.89128
9.63873	2.57031
9.65863	-1.26628
9.67852	2.02734
9.69842	0.80013
9.71831	4.08854
9.73821	3.39779
9.7581	1.01953
9.778	1.99544
9.79789	0.32552
9.81779	0.31185

9.83768	2.04818
9.85758	-2.3457
9.87747	2.48438
9.89736	-0.47721
9.91726	4.79036
9.93715	1.48747
9.95705	-2.50823
9.97694	-1.82665
9.99684	2.75644
10.01673	1.1215
10.03663	1.76356
10.05652	0.8391
10.07642	-2.61522
10.09631	-1.4117
10.11621	0.98627
10.1361	-1.07215
10.15599	0.54859
10.17589	3.67677
10.19578	0.73962
10.21568	-0.07004
10.23557	0.12814
10.25547	0.11551
10.27536	1.50677
10.29526	0.06764
10.31515	-0.51843
10.33505	1.98405
10.35494	0.36153
10.37484	0.64526
10.39473	1.29357
10.41462	0.65022
10.43452	0.30895
10.45441	2.32705
10.47431	0.43815
10.4942	1.63993
10.5141	-2.2181
10.53399	-0.38549
10.55389	1.39329
10.57378	-0.90232
10.59368	0.70707
10.61357	0.19039
10.63347	0.4384
10.65336	-0.63733
10.67325	1.61436
10.69315	0.05843
10.71304	1.6275
10.73294	1.67861
10.75283	0.01015
10.77273	-1.19011
10.79262	2.46868
10.81252	-2.1226
10.83241	0.20291
10.85231	1.21626
10.8722	1.36035
10.89209	-2.35423
10.91199	-0.88041
10.93188	2.40696
10.95178	0.90909
10.97167	1.31883
10.99157	-1.23686
11.01146	-0.92275
11.03136	1.7872
11.05125	0.80443

11.07115	2.17063
11.09104	1.19307
11.11094	0.58531
11.13083	1.15983
11.15072	0.47915
11.17062	1.35576
11.19051	1.32092
11.21041	-0.5056
11.2303	0.37622
11.2502	-2.04925
11.27009	0.82736
11.28999	0.86543
11.30988	-0.32046
11.32978	0.91552
11.34967	3.30255
11.36957	-0.89897
11.38946	1.41515
11.40935	0.01571
11.42925	0.99649
11.44914	2.75852
11.46904	-0.84404
11.48893	1.98049
11.50883	1.96647
11.52872	2.12434
11.54862	-0.22301
11.56851	0.56506
11.58841	-2.475
11.6083	0.66979
11.6282	-1.75377
11.64809	1.0258
11.66798	1.29495
11.68788	2.11098
11.70777	0.83326
11.72767	1.84199
11.74756	-1.34198
11.76746	-1.44783
11.78735	1.85778
11.80725	-0.18036
11.82714	-0.86433
11.84704	1.42044
11.86693	0.09584
11.88683	1.36446
11.90672	-1.07642
11.92661	1.84079
11.94651	0.93365
11.9664	3.44156
11.9863	1.02576
12.00619	-1.42335
12.02609	0.48975
12.04598	1.44703
12.06588	1.01575
12.08577	-1.30979
12.10567	-2.08231
12.12556	-1.20364
12.14545	2.1169
12.16535	1.95096
12.18524	2.57294
12.20514	-0.14877
12.22503	0.97848
12.24493	0.14218
12.26482	3.76422
12.28472	3.03209

12.30461	-0.39796
12.32451	0.78137
12.3444	0.42424
12.3643	1.09315
12.38419	-1.10773
12.40408	1.73305
12.42398	0.82384
12.44387	1.38859
12.46377	-0.005
12.48366	-0.54442
12.50356	1.48907
12.52345	0.26215
12.54335	2.27481
12.56324	0.15206
12.58314	1.5293
12.60303	0.97946
12.62293	-2.34642
12.64282	1.56208
12.66271	-2.46693
12.68261	1.99661
12.7025	-0.46729
12.7224	0.3813
12.74229	-0.1451
12.76219	0.35974
12.78208	3.73437
12.80198	0.06214
12.82187	1.6451
12.84177	0.53016
12.86166	2.61833
12.88156	-1.4862
12.90145	3.91448
12.92134	1.62245
12.94124	0.37729
12.96113	2.33005
12.98103	-0.67031
13.00092	-1.41547
13.02082	1.68833
13.04071	0.74526
13.06061	2.92719
13.0805	2.06745
13.1004	-1.20375
13.12029	-0.53745
13.14019	0.07156
13.16008	-0.03297
13.17997	1.3
13.19987	-0.64062
13.21976	-0.60148
13.23966	-0.01024
13.25955	-0.26797
13.27945	1.06285
13.29934	0.07595
13.31924	1.2401
13.33913	0.47196
13.35903	0.97986
13.37892	-1.07474
13.39881	0.39714
13.41871	-0.96456
13.4386	-0.82626
13.4585	-0.4588
13.47839	2.45033
13.49829	1.28134
13.51818	-0.04911

13.53808	-1.18164
13.55797	1.20106
13.57787	1.03087
13.59776	1.62027
13.61766	1.79215
13.63755	-0.23999
13.65744	-0.48139
13.67734	-1.60915
13.69723	0.73606
13.71713	-0.15934
13.73702	0.01712
13.75692	0.77581
13.77681	3.06461
13.79671	1.22201
13.8166	1.29547
13.8365	1.13456
13.85639	1.92677
13.87629	0.9169
13.89618	0.34452
13.91607	-1.21222
13.93597	-0.70126
13.95586	1.41386
13.97576	1.17482
13.99565	-1.53817
14.01555	0.04049
14.03544	1.7025
14.05534	-0.08342
14.07523	0.55254
14.09513	0.20934
14.11502	0.48071
14.13492	0.04376
14.15481	-0.46091
14.1747	0.46151
14.1946	0.71206
14.21449	1.19709
14.23439	1.36857
14.25428	0.87218
14.27418	-1.74016
14.29407	-0.6874
14.31397	0.1396
14.33386	2.67282
14.35376	0.19318
14.37365	-3.07583
14.39355	1.33919
14.41344	0.02608
14.43333	-0.37026
14.45323	3.3763
14.47312	-1.09417
14.49302	0.67607
14.51291	1.94774
14.53281	0.19511
14.5527	-3.26385
14.5726	1.50339
14.59249	0.846
14.61239	-0.22541
14.63228	1.84663
14.65217	0.73416
14.67207	-1.57208
14.69196	1.49147
14.71186	-0.40852
14.73175	0.74878
14.75165	-1.146

14.77154	-1.06682
14.79144	4.69464
14.81133	1.28424
14.83123	1.34779
14.85112	1.74988
14.87102	1.9801
14.89091	3.81969
14.9108	3.67491
14.9307	-0.31363
14.95059	0.10409
14.97049	-1.23861
14.99038	0.05411
15.01028	3.12168
15.03017	1.45028
15.05007	-0.97587
15.06996	0.36907
15.08986	0.77658
15.10975	-1.14416
15.12965	-0.33088
15.14954	1.26308
15.16943	-0.62814
15.18933	-0.10897
15.20922	1.50262
15.22912	0.59523
15.24901	0.44305
15.26891	-1.53427
15.2888	-0.87639
15.3087	1.49584
15.32859	2.88005
15.34849	-0.75438
15.36838	-1.99552
15.38828	-0.32216
15.40817	0.71913
15.42806	0.48799
15.44796	-0.10392
15.46785	1.73525
15.48775	1.32334
15.50764	3.87403
15.52754	2.13741
15.54743	1.70213
15.56733	3.80263
15.58722	1.52781
15.60712	2.43008
15.62701	1.4886
15.64691	4.7867
15.6668	-1.26416
15.68669	0.14332
15.70659	0.46225
15.72648	2.18744
15.74638	0.23033
15.76627	1.0701
15.78617	-2.24117
15.80606	-0.25036
15.82596	1.6519
15.84585	0.35105
15.86575	0.81061
15.88564	3.28058
15.90554	4.71931
15.92543	1.80387
15.94532	1.08634
15.96522	1.55111
15.98511	1.91171

16.00501	3.33482
16.0249	4.90896
16.0448	4.08206
16.06469	-0.26046
16.08459	0.68868
16.10448	-0.77364
16.12438	-0.63179
16.14427	-0.58368
16.16416	0.00608
16.18406	0.55419
16.20395	1.80541
16.22385	1.2233
16.24374	-0.41149
16.26364	1.09153
16.28353	0.11267
16.30343	0.19891
16.32332	1.19411
16.34322	3.09314
16.36311	2.00026
16.38301	-1.81883
16.4029	2.27138
16.42279	0.33868
16.44269	0.65395
16.46258	-1.68376
16.48248	-0.95564
16.50237	-0.73455
16.52227	0.84937
16.54216	0.48889
16.56206	1.53301
16.58195	2.99221
16.60185	1.13736
16.62174	0.02061
16.64164	-0.08716
16.66153	0.46512
16.68142	0.56812
16.70132	1.00315
16.72121	0.34835
16.74111	0.81732
16.761	0.77467
16.7809	0.67355
16.80079	0.04583
16.82069	2.05352
16.84058	0.68621
16.86048	-1.38944
16.88037	1.90992
16.90027	1.25615
16.92016	1.63363
16.94005	-0.69201
16.95995	2.57088
16.97984	-1.81726
16.99974	1.77376
17.01963	-0.19771
17.03953	1.77578
17.05942	-0.49542
17.07932	0.69684
17.09921	1.00342
17.11911	0.37726
17.139	-0.88496
17.1589	-0.18449
17.17879	-0.5059
17.19868	0.43181
17.21858	0.12321

17.23847	-0.92153
17.25837	-0.10892
17.27826	2.36285
17.29816	-0.25651
17.31805	-0.52049
17.33795	1.08533
17.35784	1.68192
17.37774	2.89923
17.39763	-1.52727
17.41752	1.95524
17.43742	1.27959
17.45731	-0.4705
17.47721	-1.36246
17.4971	-1.54188
17.517	1.48618
17.53689	0.59687
17.55679	1.99862
17.57668	1.88944
17.59658	2.24856
17.61647	4.90418
17.63637	7.16362
17.65626	4.99569
17.67615	7.35366
17.69605	6.53975
17.71594	9.26751
17.73584	9.0109
17.75573	11.80116
17.77563	9.00809
17.79552	9.65252
17.81542	11.81778
17.83531	7.78512
17.85521	9.32018
17.8751	4.16252
17.895	7.07778
17.91489	6.49825
17.93478	5.03331
17.95468	2.91211
17.97457	1.07737
17.99447	1.97701
18.01436	1.29331
18.03426	1.17211
18.05415	0.57696
18.07405	0.9766
18.09394	1.79811
18.11384	4.20816
18.13373	1.74842
18.15363	0.48139
18.17352	1.82374
18.19341	3.15046
18.21331	4.81051
18.2332	1.12681
18.2531	4.07355
18.27299	1.98358
18.29289	1.62251
18.31278	0.97468
18.33268	1.62339
18.35257	1.11027
18.37247	-0.50742
18.39236	2.75986
18.41226	-0.09316
18.43215	-2.26967
18.45204	0.43339

18.47194	0.70344
18.49183	1.76432
18.51173	-2.7747
18.53162	2.19559
18.55152	-1.00659
18.57141	0.18706
18.59131	1.54102
18.6112	0.65624
18.6311	0.15757
18.65099	0.77399
18.67088	-0.4478
18.69078	-1.79446
18.71067	1.51505
18.73057	-0.44134
18.75046	0.57049
18.77036	-0.55384
18.79025	1.67497
18.81015	0.16101
18.83004	1.45434
18.84994	-0.1065
18.86983	-0.8288
18.88973	0.53224
18.90962	0.12765
18.92951	0.95223
18.94941	1.16743
18.9693	0.74722
18.9892	3.06659
19.00909	-0.57083
19.02899	-0.58515
19.04888	-1.48646
19.06878	2.03796
19.08867	0.2227
19.10857	0.91165
19.12846	-0.27004
19.14836	1.05794
19.16825	-0.07828
19.18814	1.56096
19.20804	0.38719
19.22793	-0.3287
19.24783	0.705
19.26772	-1.42834
19.28762	-0.33805
19.30751	0.27277
19.32741	-0.16356
19.3473	1.08737
19.3672	0.94744
19.38709	-0.92186
19.40699	-0.22885
19.42688	1.55773
19.44677	2.94832
19.46667	-1.8227
19.48656	-0.03657
19.50646	1.77548
19.52635	2.81136
19.54625	1.90443
19.56614	4.17969
19.58604	4.52776
19.60593	3.36012
19.62583	6.60906
19.64572	10.55584
19.66562	12.24733
19.68551	14.57938

19.7054	19.1822
19.7253	15.83703
19.74519	16.93973
19.76509	15.43299
19.78498	12.37412
19.80488	12.45582
19.82477	8.54269
19.84467	8.64513
19.86456	7.96628
19.88446	3.22489
19.90435	6.06159
19.92424	0.69512
19.94414	1.55258
19.96403	2.46209
19.98393	1.60074
20.00382	0.92685
20.02372	-0.55957
20.04361	2.90712
20.06351	2.97273
20.0834	7.2779
20.1033	9.41638
20.12319	11.53921
20.14309	13.30265
20.16298	14.9671
20.18287	15.46487
20.20277	16.21782
20.22266	18.10097
20.24256	15.66639
20.26245	15.22658
20.28235	13.57842
20.30224	10.50316
20.32214	10.95393
20.34203	8.71718
20.36193	6.27209
20.38182	5.99885
20.40172	6.14748
20.42161	3.13984
20.4415	1.52282
20.4614	3.45787
20.48129	5.73144
20.50119	3.29147
20.52108	3.39835
20.54098	1.76565
20.56087	2.00793
20.58077	1.41687
20.60066	2.23726
20.62056	1.17222
20.64045	0.82592
20.66035	0.97961
20.68024	0.37809
20.70013	1.87552
20.72003	0.7271
20.73992	1.27139
20.75982	1.37296
20.77971	0.60473
20.79961	2.44066
20.8195	1.50054
20.8394	1.1802
20.85929	3.0734
20.87919	1.46139
20.89908	1.50041
20.91898	2.84672

20.93887	2.38052
20.95876	1.46119
20.97866	0.66685
20.99855	4.98188
21.01845	2.63546
21.03834	2.44528
21.05824	3.47385
21.07813	4.15346
21.09803	2.20287
21.11792	2.74707
21.13782	3.64543
21.15771	4.76775
21.1776	2.63487
21.1975	4.1374
21.21739	1.29097
21.23729	2.87683
21.25718	3.57728
21.27708	1.7621
21.29697	2.9938
21.31687	2.92341
21.33676	4.00407
21.35666	3.6316
21.37655	4.21746
21.39645	4.6627
21.41634	3.98815
21.43623	1.4386
21.45613	3.86821
21.47602	5.44887
21.49592	1.77432
21.51581	2.30289
21.53571	3.20125
21.5556	3.14128
21.5755	3.65944
21.59539	2.10988
21.61529	2.28429
21.63518	2.88578
21.65508	2.98206
21.67497	3.20855
21.69486	1.93504
21.71476	3.22403
21.73465	5.05989
21.75455	4.06763
21.77444	3.36183
21.79434	4.70811
21.81423	4.46585
21.83413	2.31213
21.85402	3.84591
21.87392	3.47344
21.89381	6.13743
21.91371	4.76496
21.9336	3.65811
21.95349	3.99398
21.97339	4.03297
21.99328	5.80112
22.01318	4.15261
22.03307	2.38952
22.05297	4.46496
22.07286	3.73833
22.09276	6.73044
22.11265	8.41006
22.13255	8.44905
22.15244	7.9047

22.17234	5.68848
22.19223	7.25351
22.21212	6.23521
22.23202	6.93045
22.25191	4.98506
22.27181	7.52405
22.2917	7.63075
22.3116	5.18015
22.33149	6.76602
22.35139	6.68522
22.37128	6.93254
22.39118	9.60173
22.41107	9.41677
22.43097	7.74221
22.45086	8.33329
22.47075	14.07019
22.49065	16.38522
22.51054	20.53359
22.53044	21.54132
22.55033	19.23656
22.57023	18.81201
22.59012	16.98121
22.61002	16.30145
22.62991	15.8196
22.64981	12.32213
22.6697	10.30904
22.68959	7.40011
22.70949	6.32972
22.72938	5.31663
22.74928	3.58479
22.76917	5.3894
22.78907	5.4336
22.80896	5.11321
22.82886	4.45949
22.84875	5.05056
22.86865	9.05309
22.88854	3.4775
22.90844	5.56336
22.92833	6.10756
22.94822	6.26634
22.96812	6.80012
22.98801	8.88599
23.00791	9.77914
23.0278	11.9848
23.0477	12.57066
23.06759	13.00548
23.08749	11.84135
23.10738	11.28659
23.12728	8.35683
23.14717	7.92186
23.16707	7.03376
23.18696	8.28629
23.20685	7.43987
23.22675	8.32781
23.24664	8.17409
23.26654	11.06204
23.28643	9.62186
23.30633	8.78585
23.32622	11.63734
23.34612	8.6607
23.36601	9.8299
23.38591	9.94181

23.4058	6.52767
23.4257	7.5927
23.44559	7.13169
23.46548	7.70193
23.48538	4.09509
23.50527	3.86845
23.52517	6.02723
23.54506	3.95164
23.56496	3.38125
23.58485	2.57128
23.60475	5.00089
23.62464	4.93572
23.64454	4.58408
23.66443	5.33661
23.68433	3.35477
23.70422	5.84167
23.72411	4.77649
23.74401	5.41965
23.7639	4.18781
23.7838	1.607
23.80369	3.01058
23.82359	5.58602
23.84348	5.02085
23.86338	6.71088
23.88327	6.66132
23.90317	7.22635
23.92306	9.86951
23.94295	7.721
23.96285	9.59332
23.98274	11.33023
24.00264	9.90567
24.02253	8.7832
24.04243	8.11907
24.06232	8.75702
24.08222	6.41059
24.10211	4.26729
24.12201	4.3844
24.1419	4.52755
24.1618	4.15509
24.18169	3.47532
24.20158	4.29035
24.22148	2.98039
24.24137	4.85792
24.26127	3.52191
24.28116	4.18069
24.30106	3.59988
24.32095	2.49825
24.34085	1.67265
24.36074	4.46685
24.38064	4.9173
24.40053	4.12816
24.42043	4.52653
24.44032	5.02906
24.46021	4.87534
24.48011	3.66953
24.5	4.9429
24.5199	6.45584
24.53979	10.349
24.55969	8.03382
24.57958	9.95823
24.59948	10.66909
24.61937	13.05183

24.63927	12.35645
24.65916	13.74439
24.67906	11.61671
24.69895	10.95779
24.71884	10.77803
24.73874	9.29618
24.75863	4.93934
24.77853	4.85333
24.79842	4.63711
24.81832	1.3688
24.83821	4.02758
24.85811	3.57178
24.878	5.15765
24.8979	2.92059
24.91779	1.87104
24.93769	3.74857
24.95758	0.05839
24.97747	3.00363
24.99737	1.55825
25.01726	4.98265
25.03716	2.15706
25.05705	1.88355
25.07695	1.19337
25.09684	2.66986
25.11674	1.53176
25.13663	2.69054
25.15653	1.22432
25.17642	2.29456
25.19631	2.54189
25.21621	1.32567
25.2361	1.77611
25.256	2.07552
25.27589	2.74993
25.29579	3.32016
25.31568	2.00499
25.33558	3.04398
25.35547	4.13505
25.37537	3.773
25.39526	3.09844
25.41516	3.35618
25.43505	3.57746
25.45494	3.42895
25.47484	3.39502
25.49473	6.7986
25.51463	8.82717
25.53452	11.10053
25.55442	12.58744
25.57431	14.88684
25.59421	18.17062
25.6141	17.78253
25.634	23.22777
25.65389	22.63655
25.67379	21.3995
25.69368	20.59474
25.71357	16.60768
25.73347	12.88625
25.75336	10.22733
25.77326	9.16215
25.79315	5.50322
25.81305	5.08388
25.83294	2.57078
25.85284	2.58894

25.87273	2.13314
25.89263	1.83879
25.91252	1.77882
25.93242	3.90114
25.95231	1.77347
25.9722	0.22391
25.9921	1.59103
26.01199	2.89564
26.03189	0.46588
26.05178	3.00487
26.07168	0.74178
26.09157	1.82243
26.11147	-0.57608
26.13136	0.31724
26.15126	1.99178
26.17115	1.24962
26.19105	2.75742
26.21094	0.2756
26.23083	1.16354
26.25073	-0.07356
26.27062	1.2466
26.29052	0.89484
26.31041	1.35034
26.33031	3.13392
26.3502	0.55289
26.3701	0.69057
26.38999	0.92196
26.40989	0.75747
26.42978	2.61899
26.44967	0.57423
26.46957	3.99296
26.48946	3.69291
26.50936	4.34073
26.52925	5.99372
26.54915	9.28209
26.56904	10.85688
26.58894	11.86912
26.60883	11.70945
26.62873	13.31537
26.64862	13.32228
26.66852	12.31353
26.68841	10.28912
26.7083	8.63445
26.7282	6.74537
26.74809	2.16874
26.76799	4.33165
26.78788	3.43723
26.80778	2.16256
26.82767	0.99722
26.84757	0.13392
26.86746	-0.00547
26.88736	2.01656
26.90725	3.33021
26.92715	2.03965
26.94704	3.19696
26.96693	0.9584
26.98683	1.06353
27.00672	0.78842
27.02662	1.69554
27.04651	0.81617
27.06641	0.66591
27.0863	-0.26043

27.1062	0.83921
27.12609	1.73048
27.14599	0.28316
27.16588	-0.13296
27.18578	1.51337
27.20567	-1.10076
27.22556	0.16527
27.24546	1.47812
27.26535	-1.17262
27.28525	-0.20883
27.30514	0.28096
27.32504	0.54152
27.34493	-0.07296
27.36483	-0.48437
27.38472	1.1875
27.40462	-0.14068
27.42451	0.94255
27.44441	0.36426
27.4643	-0.41199
27.48419	-0.42268
27.50409	3.13429
27.52398	1.74329
27.54388	1.94078
27.56377	1.20071
27.58367	0.67934
27.60356	0.09541
27.62346	0.33434
27.64335	2.32322
27.66325	0.40579
27.68314	1.14976
27.70303	0.87805
27.72293	0.02295
27.74282	0.50112
27.76272	1.22403
27.78261	-0.65729
27.80251	-0.20013
27.8224	-0.35761
27.8423	0.76089
27.86219	-0.5113
27.88209	-0.04396
27.90198	0.1004
27.92188	-1.11989
27.94177	0.75351
27.96166	0.00185
27.98156	-0.65612
28.00145	1.6598
28.02135	0.01732
28.04124	1.71853
28.06114	0.88842
28.08103	0.09471
28.10093	1.01968
28.12082	0.84562
28.14072	1.03088
28.16061	2.19523
28.18051	0.89597
28.2004	1.18519
28.22029	0.98476
28.24019	3.45092
28.26008	1.06805
28.27998	0.7372
28.29987	0.63545
28.31977	1.24195

28.33966	0.56193
28.35956	-0.90463
28.37945	1.04541
28.39935	2.70371
28.41924	0.22652
28.43914	2.69196
28.45903	5.24587
28.47892	3.60699
28.49882	5.55138
28.51871	5.34985
28.53861	4.82012
28.5585	5.65489
28.5784	5.26042
28.59829	7.26171
28.61819	3.01812
28.63808	4.10258
28.65798	4.75988
28.67787	2.95876
28.69777	0.54818
28.71766	2.41877
28.73755	-0.23676
28.75745	1.37324
28.77734	0.71754
28.79724	-0.32887
28.81713	-0.44829
28.83703	1.13534
28.85692	1.02095
28.87682	0.10961
28.89671	1.57839
28.91661	-0.03105
28.9365	3.0313
28.95639	-0.18249
28.97629	1.13489
28.99618	1.30634
29.01608	-0.26189
29.03597	0.3
29.05587	-0.37779
29.07576	1.64228
29.09566	-0.3793
29.11555	0.85953
29.13545	-0.21935
29.15534	0.56114
29.17524	-0.26253
29.19513	0.17943
29.21502	1.15263
29.23492	1.47479
29.25481	0.04175
29.27471	0.1087
29.2946	0.41003
29.3145	0.45094
29.33439	0.22623
29.35429	0.62131
29.37418	1.38618
29.39408	0.53647
29.41397	0.11905
29.43387	-0.814
29.45376	-1.41892
29.47365	1.21575
29.49355	0.62645
29.51344	-0.38472
29.53334	1.38015
29.55323	-0.14665

29.57313	0.62864
29.59302	-0.63253
29.61292	0.18443
29.63281	-0.14445
29.65271	2.79229
29.6726	-0.18242
29.6925	1.52516
29.71239	0.45149
29.73228	-0.71072
29.75218	2.43956
29.77207	0.13152
29.79197	-0.51507
29.81186	1.57793
29.83176	0.5928
29.85165	1.14413
29.87155	2.07566
29.89144	1.73116
29.91134	1.24603
29.93123	-0.37451
29.95113	0.87473
29.97102	0.64481
29.99091	-0.43928
30.01081	1.25268
30.0307	0.38734
30.0506	2.05846
30.07049	2.89625
30.09039	-1.04721
30.11028	0.46245
30.13018	2.17003
30.15007	0.35677
30.16997	1.62165
30.18986	1.28756
30.20976	0.60451
30.22965	2.91105
30.24954	1.17071
30.26944	0.08663
30.28933	0.76816
30.30923	0.3247
30.32912	1.08436
30.34902	1.16173
30.36891	2.18181
30.38881	1.53523
30.4087	1.84176
30.4286	0.86705
30.44849	0.25171
30.46838	0.39159
30.48828	1.01062
30.50817	0.14529
30.52807	-1.01693
30.54796	0.73464
30.56786	-0.06068
30.58775	-1.26279
30.60765	0.83718
30.62754	0.44757
30.64744	-0.2285
30.66733	0.65794
30.68723	0.21103
30.70712	1.71205
30.72701	0.82244
30.74691	0.79741
30.7668	2.23072
30.7867	0.55924

30.80659	1.75646
30.82649	2.69751
30.84638	0.93982
30.86628	0.20746
30.88617	1.29217
30.90607	0.67841
30.92596	-0.3421
30.94586	-0.65992
30.96575	-0.82709
30.98564	0.71892
31.00554	0.0667
31.02543	-0.29414
31.04533	1.60517
31.06522	0.62403
31.08512	0.46559
31.10501	0.29654
31.12491	1.71064
31.1448	1.2704
31.1647	1.34563
31.18459	-0.272
31.20449	1.08942
31.22438	0.23716
31.24427	0.71813
31.26417	0.0792
31.28406	1.58079
31.30396	2.13958
31.32385	-0.92151
31.34375	0.22566
31.36364	1.50816
31.38354	0.34789
31.40343	1.48963
31.42333	-0.09266
31.44322	0.3875
31.46312	0.97177
31.48301	0.1758
31.5029	0.07254
31.5228	1.67762
31.54269	1.08478
31.56259	0.95027
31.58248	1.30534
31.60238	1.00937
31.62227	0.30195
31.64217	0.37577
31.66206	1.84543
31.68196	0.13279
31.70185	-0.78817
31.72174	0.48357
31.74164	1.22927
31.76153	1.03226
31.78143	0.48108
31.80132	1.28407
31.82122	1.87873
31.84111	0.3588
31.86101	0.52117
31.8809	-0.11855
31.9008	0.03861
31.92069	-0.10632
31.94059	0.24876
31.96048	0.8955
31.98037	1.40682
32.00027	-0.01414
32.02016	0.52843

32.04006	0.53975
32.05995	1.96774
32.07985	1.14574
32.09974	0.31331
32.11964	0.98609
32.13953	1.81512
32.15943	0.89416
32.17932	-0.63619
32.19922	1.7241
32.21911	-0.14479
32.239	-0.30534
32.2589	-0.27318
32.27879	0.74335
32.29869	0.57238
32.31858	0.28162
32.33848	-0.4258
32.35837	0.95011
32.37827	1.51351
32.39816	1.64984
32.41806	0.79137
32.43795	1.75582
32.45785	0.00673
32.47774	0.27326
32.49763	0.61792
32.51753	-0.39159
32.53742	1.4114
32.55732	1.5321
32.57721	0.38717
32.59711	0.81516
32.617	0.35774
32.6369	1.07739
32.65679	0.80747
32.67669	1.5115
32.69658	1.43949
32.71648	1.3154
32.73637	0.05068
32.75626	-0.03695
32.77616	2.91187
32.79605	2.19403
32.81595	2.25223
32.83584	1.59689
32.85574	2.12383
32.87563	0.49974
32.89553	0.46419
32.91542	2.39218
32.93532	1.37746
32.95521	1.25858
32.9751	1.09803
32.995	1.18748
33.01489	1.76651
33.03479	1.15283
33.05468	0.35166
33.07458	2.34736
33.09447	3.05139
33.11437	6.88563
33.13426	8.16258
33.15416	9.3562
33.17405	9.9769
33.19395	7.62884
33.21384	7.67142
33.23373	6.5317
33.25363	7.5274

33.27352	6.01268
33.29342	4.49276
33.31331	3.24366
33.33321	3.18207
33.3531	3.15173
33.373	1.21514
33.39289	2.23688
33.41279	1.85237
33.43268	-0.34985
33.45258	-0.43748
33.47247	-0.9522
33.49236	0.47058
33.51226	1.37774
33.53215	-0.88177
33.55205	-0.03919
33.57194	-0.67057
33.59184	0.26533
33.61173	1.50852
33.63163	-1.40975
33.65152	0.28657
33.67142	-0.65253
33.69131	1.28337
33.71121	0.5526
33.7311	0.87391
33.75099	0.38794
33.77089	2.95405
33.79078	2.27536
33.81068	2.87793
33.83057	3.60029
33.85047	4.17681
33.87036	4.77417
33.89026	3.60069
33.91015	4.30743
33.93005	3.88395
33.94994	2.55422
33.96984	3.24012
33.98973	2.85831
34.00962	2.81505
34.02952	2.71343
34.04941	3.79726
34.06931	3.8739
34.0892	2.20903
34.1091	1.12043
34.12899	2.18107
34.14889	0.1254
34.16878	0.73472
34.18868	0.14449
34.20857	1.29227
34.22846	0.7979
34.24836	-0.27607
34.26825	1.93499
34.28815	1.82174
34.30804	-0.63661
34.32794	0.02353
34.34783	-0.61448
34.36773	0.36087
34.38762	-0.28477
34.40752	-0.08782
34.42741	-0.97533
34.44731	-1.57748
34.4672	0.90784
34.48709	2.42859

34.50699	1.02646
34.52688	1.6598
34.54678	0.19323
34.56667	0.09552
34.58657	1.3042
34.60646	0.77242
34.62636	-0.01023
34.64625	-0.81454
34.66615	1.7918
34.68604	-0.00371
34.70594	0.57502
34.72583	0.80923
34.74572	0.29793
34.76562	-0.13598
34.78551	0.26796
34.80541	1.32226
34.8253	0.1311
34.8452	1.28824
34.86509	1.00204
34.88499	0.24647
34.90488	-1.16596
34.92478	0.08768
34.94467	-0.17488
34.96457	0.16095
34.98446	1.35039
35.00435	0.48227
35.02425	0.54645
35.04414	0.54291
35.06404	1.10708
35.08393	0.22333
35.10383	1.64688
35.12372	1.46626
35.14362	1.65022
35.16351	0.99564
35.18341	1.88273
35.2033	0.6969
35.2232	1.26107
35.24309	0.40337
35.26298	2.07171
35.28288	2.00046
35.30277	1.62713
35.32267	3.7538
35.34256	2.27631
35.36246	2.75715
35.38235	3.87861
35.40225	3.62507
35.42214	2.88716
35.44204	2.26904
35.46193	3.56238
35.48182	3.94947
35.50172	4.47197
35.52161	2.37989
35.54151	3.66802
35.5614	4.65407
35.5813	2.89532
35.60119	4.93345
35.62109	4.33096
35.64098	3.24929
35.66088	5.0843
35.68077	5.81514
35.70067	5.40014
35.72056	5.29244

35.74045	5.61182
35.76035	5.51974
35.78024	5.37557
35.80014	6.01787
35.82003	5.96746
35.83993	6.35455
35.85982	4.08538
35.87972	4.87351
35.89961	6.39602
35.91951	5.05915
35.9394	5.58686
35.9593	3.68228
35.97919	4.04854
35.99908	5.17521
36.01898	4.16125
36.03887	4.05355
36.05877	5.21857
36.07866	4.65405
36.09856	3.47977
36.11845	4.00303
36.13835	4.27591
36.15824	2.80882
36.17814	4.46635
36.19803	2.89954
36.21793	3.42089
36.23782	3.76998
36.25771	3.23327
36.27761	2.43575
36.2975	3.24722
36.3174	1.77184
36.33729	1.59295
36.35719	3.03347
36.37708	2.27567
36.39698	2.4654
36.41687	1.70682
36.43677	1.71868
36.45666	1.55828
36.47656	0.56935
36.49645	1.11649
36.51634	1.85593
36.53624	-0.82689
36.55613	1.34926
36.57603	-0.54269
36.59592	1.15349
36.61582	-0.0101
36.63571	0.78425
36.65561	-0.31244
36.6755	1.40818
36.6954	0.08152
36.71529	-0.37575
36.73519	-0.69802
36.75508	-0.01548
36.77497	0.02082
36.79487	0.10879
36.81476	-0.38698
36.83466	1.27309
36.85455	-0.93704
36.87445	-0.66321
36.89434	0.37583
36.91424	1.77904
36.93413	-0.17754
36.95403	-0.72829

36.97392	-0.10758
36.99381	0.33563
37.01371	0.48676
37.0336	-0.78441
37.0535	0.65233
37.07339	0.81261
37.09329	1.2381
37.11318	0.55378
37.13308	-0.13617
37.15297	-0.99841
37.17287	0.02954
37.19276	-0.26585
37.21266	0.86541
37.23255	-1.80584
37.25244	-0.79002
37.27234	-0.82151
37.29223	0.24553
37.31213	-0.49515
37.33202	-0.08522
37.35192	-0.4049
37.37181	-0.83439
37.39171	-2.02994
37.4116	0.46678
37.4315	-0.59422
37.45139	-1.49421
37.47129	-0.64985
37.49118	-0.10281
37.51107	-0.01975
37.53097	-0.67151
37.55086	-0.30808
37.57076	2.12781
37.59065	-0.22841
37.61055	0.13367
37.63044	0.35989
37.65034	0.55441
37.67023	-0.11611
37.69013	0.14522
37.71002	-0.53662
37.72992	-0.72933
37.74981	1.29105
37.7697	0.60785
37.7896	0.29398
37.80949	0.89633
37.82939	1.04509
37.84928	-0.94203
37.86918	0.34123
37.88907	0.54069
37.90897	1.51572
37.92886	1.78197
37.94876	1.55295
37.96865	1.6568
37.98855	0.57789
38.00844	-0.31398
38.02833	1.12701
38.04823	1.24982
38.06812	2.22632
38.08802	1.74402
38.10791	0.57375
38.12781	0.62175
38.1477	1.9974
38.1676	2.78403
38.18749	1.06497

38.20739	1.24127
38.22728	1.69313
38.24717	3.83201
38.26707	1.76208
38.28696	0.76457
38.30686	2.152
38.32675	3.77853
38.34665	1.83165
38.36654	2.04054
38.38644	3.18643
38.40633	1.35788
38.42623	3.13821
38.44612	2.88159
38.46602	1.79635
38.48591	3.42416
38.5058	1.06709
38.5257	3.51682
38.54559	4.60693
38.56549	3.87933
38.58538	5.19339
38.60528	4.16892
38.62517	5.04027
38.64507	6.23976
38.66496	4.97049
38.68486	5.35226
38.70475	4.73404
38.72465	6.55331
38.74454	6.85696
38.76443	7.12415
38.78433	6.21946
38.80422	6.56478
38.82412	7.16009
38.84401	6.13562
38.86391	7.41843
38.8838	9.295
38.9037	8.27573
38.92359	9.45959
38.94349	10.59657
38.96338	8.63459
38.98328	10.87053
39.00317	12.13251
39.02306	10.12887
39.04296	12.34398
39.06285	14.43409
39.08275	12.63357
39.10264	12.87992
39.12254	13.74086
39.14243	15.17993
39.16233	13.69191
39.18222	15.63098
39.20212	15.28358
39.22201	18.11848
39.24191	19.05234
39.2618	16.90807
39.28169	19.41484
39.30159	21.54662
39.32148	20.57943
39.34138	22.28412
39.36127	21.10861
39.38117	23.3758
39.40106	24.26799
39.42096	25.37372

39.44085	25.83883
39.46075	26.81435
39.48064	26.37841
39.50053	29.04144
39.52043	29.07425
39.54032	31.26332
39.56022	30.47842
39.58011	33.09978
39.60001	32.43468
39.6199	32.95708
39.6398	32.8701
39.65969	34.04875
39.67959	33.73261
39.69948	34.14042
39.71938	35.47011
39.73927	33.13835
39.75916	34.8847
39.77906	34.92273
39.79895	33.95554
39.81885	34.45711
39.83874	33.38576
39.85864	35.8092
39.87853	35.17014
39.89843	34.41128
39.91832	33.42327
39.93822	33.47171
39.95811	33.15556
39.97801	33.1363
39.9979	29.99203
40.01779	31.46234
40.03769	30.29203
40.05758	28.84568
40.07748	29.41495
40.09737	26.94777
40.11727	26.19413
40.13716	24.8884
40.15706	25.26497
40.17695	23.14674
40.19685	21.91393
40.21674	23.25404
40.23664	22.59414
40.25653	20.89258
40.27642	21.28477
40.29632	20.2603
40.31621	17.92853
40.33611	19.19051
40.356	18.42124
40.3759	17.63635
40.39579	16.98687
40.41569	16.74886
40.43558	14.71396
40.45548	14.90303
40.47537	14.4723
40.49527	14.52595
40.51516	12.87647
40.53505	12.01866
40.55495	11.92648
40.57484	11.31867
40.59474	11.56503
40.61463	11.24888
40.63453	9.98482
40.65442	9.98639

40.67432	9.21191
40.69421	9.76035
40.71411	8.80358
40.734	9.03432
40.75389	7.71296
40.77379	8.37599
40.79368	7.8463
40.81358	6.47287
40.83347	8.16714
40.85337	7.26246
40.87326	6.55569
40.89316	6.81767
40.91305	5.91299
40.93295	5.33122
40.95284	6.79633
40.97274	5.84997
40.99263	7.35675
41.01252	6.62915
41.03242	5.98488
41.05231	5.10623
41.07221	5.02967
41.0921	5.6927
41.112	4.69947
41.13189	4.94583
41.15179	5.83802
41.17168	5.82916
41.19158	4.57031
41.21147	4.99375
41.23137	6.47448
41.25126	6.60105
41.27115	4.70157
41.29105	5.38543
41.31094	6.7672
41.33084	4.24272
41.35073	3.39533
41.37063	3.79794
41.39052	5.52867
41.41042	4.97815
41.43031	3.50055
41.45021	4.14274
41.4701	4.94118
41.49	3.86462
41.50989	4.7516
41.52978	5.2792
41.54968	2.68702
41.56957	3.87608
41.58947	4.32035
41.60936	4.03546
41.62926	2.31827
41.64915	3.38234
41.66905	3.83703
41.68894	2.63026
41.70884	3.9912
41.72873	2.91985
41.74863	3.14537
41.76852	4.39693
41.78841	2.98183
41.80831	3.06152
41.8282	1.64642
41.8481	3.21569
41.86799	2.71204
41.88789	3.2084

41.90778	2.65533
41.92768	3.20109
41.94757	2.71545
41.96747	2.56821
41.98736	2.75937
42.00725	0.54413
42.02715	2.31311
42.04704	2.17569
42.06694	2.55373
42.08683	3.166
42.10673	1.60101
42.12662	1.84316
42.14652	1.60076
42.16641	2.64465
42.18631	0.16753
42.2062	0.59648
42.2261	0.77005
42.24599	2.08405
42.26588	1.68953
42.28578	0.67503
42.30567	1.6968
42.32557	1.15586
42.34546	1.08868
42.36536	1.55255
42.38525	0.5162
42.40515	0.5161
42.42504	0.98973
42.44494	0.97877
42.46483	2.28007
42.48473	1.47177
42.50462	0.1424
42.52451	1.5055
42.54441	1.38397
42.5643	0.99136
42.5842	2.58807
42.60409	1.06993
42.62399	-0.49014
42.64388	0.82451
42.66378	0.30555
42.68367	0.49463
42.70357	0.07405
42.72346	1.8667
42.74336	0.70072
42.76325	1.21671
42.78314	1.45634
42.80304	1.6696
42.82293	-0.11226
42.84283	-0.73301
42.86272	1.17192
42.88262	-0.33496
42.90251	-0.31096
42.92241	0.99392
42.9423	1.79319
42.9622	0.10249
42.98209	-0.95841
43.00199	-0.73327
43.02188	0.13728
43.04177	0.64281
43.06167	-0.44586
43.08156	-0.73811
43.10146	-0.82771
43.12135	1.09783

43.14125	0.34579
43.16114	0.59324
43.18104	0.06412
43.20093	-0.03845
43.22083	1.5928
43.24072	0.96828
43.26062	1.1088
43.28051	-0.42837
43.3004	1.38801
43.3203	0.6048
43.34019	0.12823
43.36009	-0.02088
43.37998	0.49597
43.39988	0.90274
43.41977	0.54836
43.43967	1.21927
43.45956	0.03523
43.47946	0.72018
43.49935	0.50846
43.51924	1.05154
43.53914	0.79773
43.55903	0.49705
43.57893	0.29013
43.59882	0.15611
43.61872	-0.09248
43.63861	0.63591
43.65851	0.47296
43.6784	-1.02391
43.6983	1.24957
43.71819	-0.75338
43.73809	1.61311
43.75798	0.81787
43.77787	0.37133
43.79777	0.24229
43.81766	0.3214
43.83756	3.42118
43.85745	-0.69272
43.87735	1.48491
43.89724	0.5166
43.91714	1.18361
43.93703	-0.56614
43.95693	1.93924
43.97682	-0.55545
43.99672	1.50187
44.01661	0.27788
44.0365	-0.46178
44.0564	0.60058
44.07629	-0.0871
44.09619	-0.80607
44.11608	0.42804
44.13598	-0.17642
44.15587	0.7295
44.17577	1.15622
44.19566	1.87979
44.21556	-1.79251
44.23545	0.59768
44.25535	2.22221
44.27524	1.24256
44.29513	0.13267
44.31503	0.76756
44.33492	0.36598
44.35482	1.4175

44.37471	-0.32787
44.39461	0.49966
44.4145	2.13447
44.4344	1.23802
44.45429	-0.28865
44.47419	0.29924
44.49408	0.81942
44.51398	-0.13437
44.53387	1.21912
44.55376	1.21843
44.57366	1.37919
44.59355	2.01391
44.61345	-0.12222
44.63334	2.3406
44.65324	2.66279
44.67313	1.66726
44.69303	1.30193
44.71292	2.92618
44.73282	1.89418
44.75271	2.66425
44.7726	2.12181
44.7925	2.66271
44.81239	3.58901
44.83229	1.56218
44.85218	2.42077
44.87208	2.03456
44.89197	1.10147
44.91187	1.67879
44.93176	3.06861
44.95166	2.90113
44.97155	1.12948
44.99145	1.15471
45.01134	2.73202
45.03123	3.22079
45.05113	3.30851
45.07102	1.91187
45.09092	1.78084
45.11081	3.88419
45.13071	3.02921
45.1506	3.12736
45.1705	3.67862
45.19039	4.00593
45.21029	4.73949
45.23018	2.32201
45.25008	3.27432
45.26997	2.62246
45.28986	3.2831
45.30976	3.57916
45.32965	3.76584
45.34955	4.44211
45.36944	4.24337
45.38934	3.94047
45.40923	5.57507
45.42913	4.99092
45.44902	4.07864
45.46892	5.06741
45.48881	5.2593
45.50871	6.56577
45.5286	6.70037
45.54849	6.13184
45.56839	7.48519
45.58828	7.04166

45.60818	5.93146
45.62807	9.38897
45.64797	8.14336
45.66786	7.10608
45.68776	8.60005
45.70765	7.55756
45.72755	8.39008
45.74744	8.68613
45.76734	10.5551
45.78723	9.63761
45.80712	9.60033
45.82702	9.72971
45.84691	11.4216
45.86681	10.75931
45.8867	10.00328
45.9066	10.40871
45.92649	10.03809
45.94639	11.01122
45.96628	10.53644
45.98618	11.41582
46.00607	12.11291
46.02596	11.60167
46.04586	12.49667
46.06575	12.35522
46.08565	12.69293
46.10554	11.96294
46.12544	12.24857
46.14533	14.90399
46.16523	15.14795
46.18512	15.04295
46.20502	15.04733
46.22491	15.53608
46.24481	16.26963
46.2647	16.98234
46.28459	16.28359
46.30449	14.59005
46.32438	14.87046
46.34428	15.94775
46.36417	16.58234
46.38407	15.72734
46.40396	15.76817
46.42386	15.16316
46.44375	15.28733
46.46365	14.26566
46.48354	14.09294
46.50344	13.01398
46.52333	14.01314
46.54322	12.59043
46.56312	12.76146
46.58301	11.42729
46.60291	10.7129
46.6228	10.84748
46.6427	11.53413
46.66259	11.09579
46.68249	9.17307
46.70238	8.59931
46.72228	9.72346
46.74217	9.59241
46.76207	8.28948
46.78196	7.74175
46.80185	8.03778
46.82175	6.9588

46.84164	6.43191
46.86154	6.32689
46.88143	5.62812
46.90133	6.84081
46.92122	6.56912
46.94112	5.54222
46.96101	6.32261
46.98091	5.01967
47.0008	5.83131
47.0207	4.99191
47.04059	3.9598
47.06048	4.53706
47.08038	5.42682
47.10027	5.05616
47.12017	1.74279
47.14006	3.30963
47.15996	3.38689
47.17985	4.01622
47.19975	2.86952
47.21964	3.00927
47.23954	1.72714
47.25943	2.79918
47.27932	3.34517
47.29922	2.80783
47.31911	2.66632
47.33901	2.96752
47.3589	2.51351
47.3788	2.21054
47.39869	1.54819
47.41859	2.29208
47.43848	3.06202
47.45838	1.61841
47.47827	1.57064
47.49817	2.41869
47.51806	1.31883
47.53795	2.13045
47.55785	2.87436
47.57774	1.20161
47.59764	1.98197
47.61753	0.53837
47.63743	2.60518
47.65732	1.60428
47.67722	1.46796
47.69711	1.93058
47.71701	3.69008
47.7369	3.84541
47.7568	6.4174
47.77669	7.92688
47.79658	8.24886
47.81648	11.86249
47.83637	13.67925
47.85627	16.38141
47.87616	21.26065
47.89606	21.49405
47.91595	22.59202
47.93585	24.79936
47.95574	23.14732
47.97564	23.36505
47.99553	22.39007
48.01543	21.92029
48.03532	19.37238
48.05521	18.24632

48.07511	16.84422
48.095	16.23898
48.1149	14.98789
48.13479	12.67949
48.15469	12.02733
48.17458	10.61474
48.19448	7.85318
48.21437	4.19055
48.23427	2.69978
48.25416	2.32358
48.27406	1.0984
48.29395	1.88361
48.31384	0.77817
48.33374	-0.51479
48.35363	0.69743
48.37353	-0.66851
48.39342	-0.99281
48.41332	0.09431
48.43321	1.22828
48.45311	0.25282
48.473	-0.28518
48.4929	1.89553
48.51279	-0.07486
48.53268	-0.92552
48.55258	0.40084
48.57247	-0.77808
48.59237	1.12105
48.61226	-0.36532
48.63216	0.50238
48.65205	-1.01024
48.67195	1.75306
48.69184	0.89642
48.71174	-0.3406
48.73163	0.16699
48.75153	-1.36211
48.77142	0.74532
48.79131	-0.10276
48.81121	-0.42519
48.8311	0.02274
48.851	0.70965
48.87089	-0.00524
48.89079	0.4716
48.91068	0.36903
48.93058	0.60843
48.95047	0.57451
48.97037	-1.06538
48.99026	0.19547
49.01016	-0.00225
49.03005	0.37798
49.04994	-1.04401
49.06984	0.32555
49.08973	0.09087
49.10963	0.11653
49.12952	-0.79015
49.14942	-0.77266
49.16931	0
49.18921	0.70464
49.2091	0.3103
49.229	-0.09449
49.24889	1.85488
49.26879	0.18443
49.28868	0.65459

49.30857	-0.5211
49.32847	0.25112
49.34836	0.26292
49.36826	1.76429
49.38815	0.18752
49.40805	-0.23821
49.42794	-0.21602
49.44784	0.41553
49.46773	1.28666
49.48763	0.42862
49.50752	1.77891
49.52742	0.66566
49.54731	2.27636
49.5672	0.01206
49.5871	1.75297
49.60699	0.64492
49.62689	0.77645
49.64678	0.91319
49.66668	0.88847
49.68657	1.49396
49.70647	-0.83805
49.72636	1.68931
49.74626	0.15938
49.76615	0.2857
49.78605	0.92244
49.80594	-1.21165
49.82583	-0.06449
49.84573	0.00975
49.86562	0.09441
49.88552	-0.36259
49.90541	-1.48105
49.92531	1.82757
49.9452	-0.02006
49.9651	0.10628
49.98499	1.12845
50.00489	-1.27126
50.02478	-1.13971
50.04467	1.471
50.06457	0.77443
50.08446	0.07265
50.10436	0.65733
50.12425	-0.8882
50.14415	0.99857
50.16404	1.14055
50.18394	1.03774
50.20383	-0.38278
50.22373	-0.18351
50.24362	-0.6509
50.26352	1.41297
50.28341	0.206
50.3033	-1.36555
50.3232	0.51603
50.34309	0.32469
50.36299	1.46669
50.38288	0.14515
50.40278	0.86007
50.42267	0.08541
50.44257	0.48784
50.46246	0.70276
50.48236	-0.47814
50.50225	-0.133
50.52215	-0.29827

50.54204	0
50.56193	0.62929
50.58183	1.15451
50.60172	0.56004
50.62162	-0.34163
50.64151	-0.55049
50.66141	1.29804
50.6813	1.1102
50.7012	1.72976
50.72109	-0.12975
50.74099	0.28166
50.76088	0.86505
50.78078	0.72458
50.80067	-0.3585
50.82056	0.39706
50.84046	0.50688
50.86035	1.75743
50.88025	0.53411
50.90014	0.66506
50.92004	-0.26639
50.93993	-1.00503
50.95983	1.46996
50.97972	0.46589
50.99962	-1.86621
51.01951	1.64033
51.03941	1.24593
51.0593	0.35162
51.07919	-0.19883
51.09909	0.06852
51.11898	-0.18486
51.13888	0.3379
51.15877	0.68888
51.17867	0.67017
51.19856	0.70885
51.21846	0.28409
51.23835	0.64068
51.25825	-1.09639
51.27814	1.1354
51.29803	0.11208
51.31793	-1.20802
51.33782	0.27406
51.35772	0.80833
51.37761	0.48332
51.39751	-0.20096
51.4174	0.16694
51.4373	0.51411
51.45719	1.10096
51.47709	-0.51001
51.49698	2.06662
51.51688	0.67982
51.53677	-0.25377
51.55666	0.47421
51.57656	0.01999
51.59645	1.11275
51.61635	1.01811
51.63624	1.10066
51.65614	1.14164
51.67603	0.32855
51.69593	1.40619
51.71582	1.29642
51.73572	0.27531
51.75561	1.02512

51.77551	1.72295
51.7954	0.23859
51.81529	0.44704
51.83519	0.91601
51.85508	3.18196
51.87498	1.46884
51.89487	1.52665
51.91477	0.45957
51.93466	1.08529
51.95456	0.60695
51.97445	0.936
51.99435	0.76516
52.01424	0.09441
52.03414	1.5644
52.05403	0.28969
52.07392	0.10362
52.09382	0.92287
52.11371	1.42972
52.13361	0.82729
52.1535	0.89163
52.1734	1.41962
52.19329	2.37479
52.21319	0.68423
52.23308	2.06148
52.25298	2.11071
52.27287	1.57671
52.29277	1.23031
52.31266	1.59755
52.33255	1.64719
52.35245	2.41568
52.37234	2.26239
52.39224	0.63526
52.41213	1.6853
52.43203	1.47504
52.45192	1.08779
52.47182	2.25273
52.49171	1.30839
52.51161	0.385
52.5315	1.97212
52.55139	1.06976
52.57129	2.81334
52.59118	1.83306
52.61108	1.10289
52.63097	1.25823
52.65087	1.37722
52.67076	1.64736
52.69066	3.21968
52.71055	1.58377
52.73045	2.92192
52.75034	1.61955
52.77024	2.96833
52.79013	2.85888
52.81002	3.70786
52.82992	3.23924
52.84981	2.53114
52.86971	3.26064
52.8896	3.62046
52.9095	4.18871
52.92939	5.74144
52.94929	3.0599
52.96918	3.66492
52.98908	1.8638

53.00897	3.8909
53.02887	3.25665
53.04876	4.64855
53.06865	3.74368
53.08855	3.86495
53.10844	3.44986
53.12834	4.27447
53.14823	3.4898
53.16813	3.34586
53.18802	3.94682
53.20792	3.86039
53.22781	3.60739
53.24771	3.69824
53.2676	5.80483
53.2875	6.76569
53.30739	4.30477
53.32728	6.06792
53.34718	8.00305
53.36707	5.89141
53.38697	6.0455
53.40686	6.40282
53.42676	7.15607
53.44665	6.8261
53.46655	6.92332
53.48644	6.52585
53.50634	9.33681
53.52623	7.57497
53.54613	5.89136
53.56602	6.93181
53.58591	6.57653
53.60581	6.27344
53.6257	8.1892
53.6456	8.0582
53.66549	8.27626
53.68539	6.34338
53.70528	7.78561
53.72518	6.19669
53.74507	7.92038
53.76497	8.08168
53.78486	6.66495
53.80475	7.57125
53.82465	7.33703
53.84454	6.22791
53.86444	7.38974
53.88433	7.95791
53.90423	6.61474
53.92412	7.60501
53.94402	7.86621
53.96391	6.78377
53.98381	7.39935
54.0037	8.77546
54.0236	7.53188
54.04349	7.45507
54.06338	7.0242
54.08328	6.64031
54.10317	7.57945
54.12307	6.71661
54.14296	8.08304
54.16286	7.63708
54.18275	7.44643
54.20265	6.96943
54.22254	9.0967

54.24244	7.75012
54.26233	8.70053
54.28223	8.5677
54.30212	9.02352
54.32201	11.06279
54.34191	12.15945
54.3618	10.48538
54.3817	11.09267
54.40159	11.92924
54.42149	14.19299
54.44138	14.65998
54.46128	14.93436
54.48117	13.71406
54.50107	17.35324
54.52096	19.58107
54.54086	21.55901
54.56075	25.0058
54.58064	31.52041
54.60054	36.95179
54.62043	45.52912
54.64033	56.018
54.66022	67.84554
54.68012	82.32943
54.70001	94.12593
54.71991	106.1309
54.7398	113.5057
54.7597	120.2296
54.77959	123.7974
54.79949	123.0319
54.81938	122.0791
54.83927	119.9336
54.85917	119.632
54.87906	112.7941
54.89896	105.9562
54.91885	100.7122
54.93875	91.95271
54.95864	83.37561
54.97854	72.73611
54.99843	66.41443
55.01833	56.90535
55.03822	43.73492
55.05811	32.48648
55.07801	26.50377
55.0979	22.63575
55.1178	19.16367
55.13769	17.66045
55.15759	13.81359
55.17748	13.4512
55.19738	13.91706
55.21727	12.17468
55.23717	11.94804
55.25706	11.78401
55.27696	11.67737
55.29685	12.32606
55.31674	13.48005
55.33664	12.61853
55.35653	12.28317
55.37643	13.99478
55.39632	14.35754
55.41622	17.35583
55.43611	17.95318
55.45601	23.15481

55.4759	25.57009
55.4958	28.35527
55.51569	33.7916
55.53559	38.25928
55.55548	43.16458
55.57537	46.85123
55.59527	48.48591
55.61516	52.62584
55.63506	52.97931
55.65495	54.31196
55.67485	52.02482
55.69474	50.51893
55.71464	48.57033
55.73453	46.18424
55.75443	45.48044
55.77432	43.20372
55.79422	40.21347
55.81411	36.97843
55.834	33.46214
55.8539	31.18023
55.87379	28.01812
55.89369	21.52788
55.91358	19.75639
55.93348	17.55262
55.95337	15.16655
55.97327	13.4732
55.99316	12.9361
56.01306	13.15421
56.03295	13.03899
56.05285	11.34565
56.07274	12.49085
56.09263	14.00064
56.11253	11.76043
56.13242	13.79626
56.15232	13.08731
56.17221	14.7794
56.19211	15.30483
56.212	15.53859
56.2319	18.21507
56.25179	19.14154
56.27169	22.00031
56.29158	26.46325
56.31148	30.82203
56.33137	39.62352
56.35126	55.19584
56.37116	75.29942
56.39105	99.42904
56.41095	127.866
56.43084	163.6154
56.45074	198.094
56.47063	239.5205
56.49053	273.3584
56.51042	290.1339
56.53032	304.9042
56.55021	305.1119
56.5701	301.7728
56.59	293.9858
56.60989	284.7561
56.62979	275.0316
56.64968	260.9737
56.66958	248.1399
56.68947	230.9206

56.70937	211.5242
56.72926	193.1122
56.74916	174.445
56.76905	157.2361
56.78895	133.595
56.80884	98.0788
56.82873	65.52619
56.84863	44.7392
56.86852	34.80118
56.88842	30.18608
56.90831	27.22202
56.92821	24.89339
56.9481	23.66371
56.968	21.73612
56.98789	20.21478
57.00779	18.95386
57.02768	19.70857
57.04758	17.33308
57.06747	17.33258
57.08736	17.42584
57.10726	17.28473
57.12715	16.02904
57.14705	15.38272
57.16694	13.55412
57.18684	14.35572
57.20673	12.68858
57.22663	12.1152
57.24652	12.45327
57.26642	13.66635
57.28631	16.23359
57.30621	16.75397
57.3261	18.90455
57.34599	21.35722
57.36589	25.05469
57.38578	28.06466
57.40568	31.59025
57.42557	35.53773
57.44547	35.48
57.46536	37.23998
57.48526	39.38538
57.50515	37.09328
57.52505	37.49389
57.54494	37.30076
57.56484	35.26908
57.58473	34.34679
57.60462	32.86721
57.62452	29.68971
57.64441	29.29867
57.66431	26.5691
57.6842	23.6364
57.7041	22.96412
57.72399	20.01581
57.74389	17.21854
57.76378	12.64002
57.78368	11.42608
57.80357	8.43091
57.82346	7.31073
57.84336	6.4718
57.86325	5.77872
57.88315	6.27834
57.90304	3.90297
57.92294	3.22551

57.94283	3.58452
57.96273	3.35499
57.98262	4.17754
58.00252	4.02614
58.02241	4.16641
58.04231	3.5046
58.0622	2.43654
58.08209	3.08203
58.10199	2.1546
58.12188	2.63863
58.14178	2.44558
58.16167	2.05983
58.18157	2.84596
58.20146	3.12167
58.22136	2.29842
58.24125	2.02206
58.26115	0.86549
58.28104	1.64121
58.30094	0.73464
58.32083	2.67183
58.34072	2.37465
58.36062	1.08788
58.38051	1.59279
58.40041	1.69665
58.4203	1.2224
58.4402	1.46169
58.46009	-0.09069
58.47999	0.37777
58.49988	0.05457
58.51978	0.15324
58.53967	0.69463
58.55957	0.20998
58.57946	-1.05592
58.59935	1.32922
58.61925	0.37062
58.63914	-0.62443
58.65904	0.15655
58.67893	-1.41662
58.69883	-0.1773
58.71872	0.65578
58.73862	-0.81842
58.75851	-0.09992
58.77841	0.35296
58.7983	-0.9077
58.8182	-1.93398
58.83809	-0.9863
58.85798	-0.55424
58.87788	-2.08051
58.89777	-1.62761
58.91767	-1.76325
58.93756	-1.18535
58.95746	-1.09182
58.97735	-1.98786
58.99725	-1.34745
59.01714	-2.49349
59.03704	-2.31141
59.05693	-0.25953
59.07682	-2.03577
59.09672	-2.35888
59.11661	-1.45803
59.13651	-1.68218
59.1564	-0.98445

59.1763	-1.23464
59.19619	-1.6619
59.21609	-2.09437
59.23598	-2.52163
59.25588	-2.10514
59.27577	-1.44906
59.29567	-1.98568
59.31556	-0.73064
59.33545	0.05045
59.35535	-1.59033
59.37524	-1.68423
59.39514	-3.12188
59.41503	-2.06995
59.43493	-2.0128
59.45482	-1.15878
59.47472	-1.17975
59.49461	-2.02364
59.51451	-1.96127
59.5344	-2.28431
59.5543	-1.45632
59.57419	-1.65957
59.59408	-2.08156
59.61398	-3.9723
59.63387	-2.6495
59.65377	-1.56628
59.67366	-2.2018
59.69356	-3.01441
59.71345	-2.23326
59.73335	-1.98336
59.75324	-2.572
59.77314	-1.07209
59.79303	-1.79093
59.81293	-2.95768
59.83282	-1.97339
59.85271	-3.14535
59.87261	-2.37459
59.8925	-1.99967
59.9124	-1.20807
59.93229	-1.96335
59.95219	-1.53633
59.97208	-2.79681
59.99198	-1.31249
60.01187	-2.12505
60.03177	-2.56781
60.05166	-1.7189
60.07156	-2.60436
60.09145	-1.60441
60.11134	-1.84924
60.13124	-1.79719
60.15113	-2.72952
60.17103	-2.03163
60.19092	-1.92749
60.21082	-3.77648
60.23071	-2.36504
60.25061	-0.64631
60.2705	-2.08903
60.2904	-1.42238
60.31029	-1.89113
60.33018	-2.66197
60.35008	-0.92239
60.36997	-3.04218
60.38987	-0.26093

60.40976	-1.90676
60.42966	-0.75571
60.44955	-0.26611
60.46945	-0.5838
60.48934	0.02559
60.50924	-0.83376
60.52913	0.52043
60.54903	-0.44308
60.56892	0.0205
60.58881	0.08824
60.60871	1.08307
60.6286	-0.34397
60.6485	-0.35434
60.66839	0.66133
60.68829	0.82805
60.70818	1.41664
60.72808	1.0417
60.74797	0.95322
60.76787	0.73453
60.78776	2.46898
60.80766	1.6878
60.82755	3.87016
60.84744	2.89107
60.86734	3.6724
60.88723	3.78186
60.90713	4.10486
60.92702	5.06329
60.94692	6.93317
60.96681	6.29264
60.98671	6.54795
61.0066	7.64701
61.0265	9.48566
61.04639	10.53264
61.06629	12.45463
61.08618	15.31933
61.10607	18.6059
61.12597	21.96019
61.14586	28.21032
61.16576	36.77295
61.18565	43.98662
61.20555	57.05446
61.22544	70.65877
61.24534	83.11724
61.26523	98.47676
61.28513	113.8259
61.30502	126.3469
61.32492	135.5085
61.34481	140.3576
61.3647	146.1494
61.3846	154.7329
61.40449	157.6289
61.42439	161.9207
61.44428	165.2855
61.46418	170.4888
61.48407	175.0619
61.50397	181.661
61.52386	187.3331
61.54376	195.375
61.56365	205.745
61.58354	218.5733
61.60344	236.6256
61.62333	265.0737

61.64323	312.126
61.66312	396.1939
61.68302	550.3816
61.70291	824.9339
61.72281	1197.33
61.7427	1655.497
61.7626	2206.299
61.78249	2865.128
61.80239	3572.414
61.82228	4251.112
61.84217	4779.842
61.86207	5036.488
61.88196	5116.264
61.90186	5082.316
61.92175	4991.926
61.94165	4874.947
61.96154	4735.395
61.98144	4579.875
62.00133	4434.734
62.02123	4250.36
62.04112	4038.021
62.06102	3821.194
62.08091	3571.866
62.1008	3294.361
62.1207	2964.392
62.14059	2495.497
62.16049	1804.336
62.18038	1136.206
62.20028	748.0862
62.22017	557.7272
62.24007	443.5452
62.25996	373.2903
62.27986	328.7906
62.29975	296.0305
62.31965	273.0308
62.33954	253.9895
62.35943	233.4637
62.37933	219.5526
62.39922	207.0686
62.41912	195.3866
62.43901	183.2932
62.45891	176.8092
62.4788	168.5022
62.4987	161.1484
62.51859	155.3415
62.53849	151.9668
62.55838	144.8474
62.57828	140.3113
62.59817	134.7231
62.61806	134.8745
62.63796	131.3958
62.65785	126.219
62.67775	124.459
62.69764	122.6886
62.71754	119.2098
62.73743	119.2466
62.75733	117.846
62.77722	114.5287
62.79712	113.3832
62.81701	113.217
62.8369	112.4986
62.8568	112.0563

62.87669	108.9838
62.89659	108.4218
62.91648	108.5784
62.93638	105.5216
62.95627	102.7355
62.97617	101.3245
62.99606	99.80411
63.01596	98.41392
63.03585	98.59145
63.05575	98.85231
63.07564	98.32164
63.09553	94.29637
63.11543	94.89108
63.13532	95.97557
63.15522	93.31545
63.17511	93.72324
63.19501	94.65206
63.2149	92.86753
63.2348	92.83319
63.25469	90.31466
63.27459	91.28592
63.29448	89.7782
63.31438	90.76546
63.33427	89.21646
63.35416	88.87599
63.37406	87.52008
63.39395	85.39874
63.41385	82.74113
63.43374	84.9431
63.45364	83.05671
63.47353	84.30594
63.49343	80.16994
63.51332	79.39351
63.53322	77.82039
63.55311	76.71622
63.57301	75.79975
63.5929	74.85742
63.61279	73.5455
63.63269	72.85356
63.65258	70.34931
63.67248	68.85047
63.69237	68.01328
63.71227	69.02003
63.73216	66.05301
63.75206	65.75806
63.77195	65.77581
63.79185	65.88229
63.81174	63.70772
63.83164	65.41354
63.85153	64.95811
63.87142	60.74765
63.89132	63.31864
63.91121	61.29607
63.93111	60.95078
63.951	59.98069
63.9709	59.76599
63.99079	57.96816
64.01069	58.56114
64.03058	56.64911
64.05048	56.01331
64.07037	55.63292
64.09027	54.9923

64.11016	56.17479
64.13005	55.49289
64.14995	56.52473
64.16984	53.90051
64.18974	53.14106
64.20963	53.36098
64.22953	53.10713
64.24942	52.75973
64.26932	52.67293
64.28921	51.52904
64.30911	52.08325
64.329	50.6585
64.34889	49.94748
64.36879	50.66373
64.38868	49.90622
64.40858	49.37286
64.42847	47.88657
64.44837	49.43173
64.46826	48.2479
64.48816	49.49136
64.50805	47.82355
64.52795	47.41115
64.54784	47.45206
64.56774	47.5817
64.58763	47.53967
64.60752	46.87803
64.62742	47.1593
64.64731	44.87826
64.66721	45.13908
64.6871	45.17093
64.707	46.57276
64.72689	45.06333
64.74679	44.35617
64.76668	45.77941
64.78658	45.69244
64.80647	45.51711
64.82637	44.72218
64.84626	45.16703
64.86615	44.51832
64.88605	44.99481
64.90594	43.50795
64.92584	43.07336
64.94573	42.68064
64.96563	41.20998
64.98552	41.78118
65.00542	42.51924
65.02531	42.16374
65.04521	42.04802
65.0651	41.59395
65.085	42.85882
65.10489	43.06139
65.12478	42.25373
65.14468	41.47752
65.16457	40.74316
65.18447	41.70691
65.20436	42.49378
65.22426	41.95791
65.24415	40.66183
65.26405	42.70969
65.28394	41.20565
65.30384	41.70702
65.32373	40.78149

65.34363	41.12179
65.36352	42.06123
65.38341	40.90191
65.40331	44.18549
65.4232	42.16718
65.4431	43.14385
65.46299	43.02175
65.48289	42.9936
65.50278	43.56981
65.52268	43.5212
65.54257	43.18113
65.56247	46.45062
65.58236	46.47551
65.60225	47.54226
65.62215	48.94253
65.64204	50.72842
65.66194	51.24887
65.68183	51.27993
65.70173	55.0143
65.72162	57.73845
65.74152	60.2805
65.76141	65.11441
65.78131	68.8808
65.8012	75.84009
65.8211	84.07041
65.84099	96.82696
65.86088	115.4431
65.88078	141.9969
65.90067	177.8321
65.92057	221.5061
65.94046	274.3678
65.96036	333.1411
65.98025	397.6125
66.00015	462.1779
66.02004	513.6393
66.03994	538.8613
66.05983	547.5991
66.07973	547.6965
66.09962	542.768
66.11951	530.6002
66.13941	515.0471
66.1593	501.9734
66.1792	478.9675
66.19909	459.4671
66.21899	441.295
66.23888	415.5137
66.25878	390.342
66.27867	362.0558
66.29857	328.1293
66.31846	282.1612
66.33836	224.4746
66.35825	165.5539
66.37814	122.003
66.39804	99.52535
66.41793	86.66243
66.43783	80.84657
66.45772	79.69236
66.47762	82.22063
66.49751	83.40534
66.51741	88.22566
66.5373	91.82742
66.5572	97.91895

66.57709	102.4794
66.59699	104.2432
66.61688	104.8301
66.63677	104.1463
66.65667	103.4992
66.67656	101.3679
66.69646	100.3045
66.71635	96.37674
66.73625	94.57935
66.75614	91.34985
66.77604	91.32367
66.79593	87.14663
66.81583	85.62604
66.83572	80.98063
66.85561	77.57499
66.87551	70.13308
66.8954	64.46219
66.9153	58.02587
66.93519	53.70432
66.95509	49.5965
66.97498	50.87949
66.99488	48.61579
67.01477	47.52937
67.03467	50.60459
67.05456	50.3102
67.07446	47.41705
67.09435	48.51366
67.11424	47.60526
67.13414	48.546
67.15403	48.84109
67.17393	49.24054
67.19382	48.26518
67.21372	51.04521
67.23361	49.26813
67.25351	50.0225
67.2734	50.02704
67.2933	49.67761
67.31319	50.51066
67.33309	49.57827
67.35298	51.76065
67.37287	50.67759
67.39277	50.3395
67.41266	51.23598
67.43256	52.03369
67.45245	50.81075
67.47235	51.24945
67.49224	53.12584
67.51214	55.07533
67.53203	55.03021
67.55193	53.54257
67.57182	53.77387
67.59172	54.08347
67.61161	55.83076
67.6315	53.76574
67.6514	55.61757
67.67129	54.12062
67.69119	55.74886
67.71108	56.04395
67.73098	57.34444
67.75087	59.10865
67.77077	56.83137
67.79066	59.04908

67.81056	60.03259
67.83045	59.3392
67.85035	61.06267
67.87024	61.24465
67.89013	61.06744
67.91003	62.58312
67.92992	63.35419
67.94982	64.7192
67.96971	64.56876
67.98961	67.49143
68.0095	65.20073
68.0294	68.06126
68.04929	68.23969
68.06919	71.10058
68.08908	69.23249
68.10897	73.69271
68.12887	73.74166
68.14876	73.53558
68.16866	74.3453
68.18855	76.32188
68.20845	77.94447
68.22834	80.46828
68.24824	80.0704
68.26813	83.60499
68.28803	83.46789
68.30792	86.02889
68.32782	86.7984
68.34771	89.22955
68.3676	90.57755
68.3875	92.33198
68.40739	94.70118
68.42729	96.07576
68.44718	100.6068
68.46708	103.0963
68.48697	106.8725
68.50687	108.6228
68.52676	112.217
68.54666	115.0666
68.56655	119.9425
68.58645	121.0633
68.60634	125.3562
68.62623	130.9721
68.64613	133.4372
68.66602	140.4338
68.68592	146.0399
68.70581	152.9638
68.72571	161.0859
68.7456	168.7603
68.7655	183.6223
68.78539	196.6042
68.80529	212.4562
68.82518	233.2562
68.84508	258.7648
68.86497	284.086
68.88486	312.3033
68.90476	348.1249
68.92465	383.9571
68.94455	417.5446
68.96444	457.7678
68.98434	493.2724
69.00423	542.449
69.02413	603.2248

69.04402	686.7664
69.06392	795.4019
69.08381	947.7303
69.10371	1178.444
69.1236	1651.195
69.14349	2663.967
69.16339	3904.613
69.18328	5307.531
69.20318	7051.574
69.22307	9082.404
69.24297	11367.45
69.26286	13611.79
69.28276	15165.39
69.30265	15795.96
69.32255	16055.35
69.34244	16261.62
69.36233	16507.75
69.38223	16830.83
69.40212	17273.49
69.42202	17914.03
69.44191	18591.57
69.46181	19191.56
69.4817	19381.55
69.5016	18983.3
69.52149	18318.9
69.54139	17503.68
69.56128	16447.21
69.58118	14572.66
69.60107	11377
69.62096	8850.892
69.64086	7683.127
69.66075	7063.403
69.68065	6609.148
69.70054	6170.393
69.72044	5751.337
69.74033	5295.207
69.76023	4764.594
69.78012	3814.1
69.80002	2329.221
69.81991	1248.853
69.83981	799.2762
69.8597	603.4708
69.87959	496.7332
69.89949	432.7041
69.91938	386.071
69.93928	348.7038
69.95917	321.9669
69.97907	295.8239
69.99896	272.3477
70.01886	257.3093
70.03875	239.1303
70.05865	225.9932
70.07854	214.6376
70.09844	201.9748
70.11833	191.4267
70.13822	185.0455
70.15812	175.7061
70.17801	168.8305
70.19791	164.6164
70.2178	156.6109
70.2377	151.0274
70.25759	145.6212

70.27749	141.9182
70.29738	136.7779
70.31728	131.9347
70.33717	127.3104
70.35707	126.8841
70.37696	120.4633
70.39685	118.3394
70.41675	113.9085
70.43664	110.7329
70.45654	108.2763
70.47643	105.2156
70.49633	101.8009
70.51622	100.0375
70.53612	96.55022
70.55601	95.9121
70.57591	93.78455
70.5958	89.58946
70.6157	88.34766
70.63559	85.92895
70.65548	82.04685
70.67538	81.73784
70.69527	79.96544
70.71517	78.83384
70.73506	74.81177
70.75496	74.04508
70.77485	71.46605
70.79475	71.46009
70.81464	68.71472
70.83454	68.50597
70.85443	65.79217
70.87432	62.84416
70.89422	63.63589
70.91411	60.45904
70.93401	60.46984
70.9539	58.48602
70.9738	58.55444
70.99369	55.4876
71.01359	54.9626
71.03348	54.56275
71.05338	54.41827
71.07327	50.51353
71.09317	51.40583
71.11306	50.42329
71.13295	50.21695
71.15285	48.84932
71.17274	48.57559
71.19264	45.54681
71.21253	45.80986
71.23243	44.36473
71.25232	42.95101
71.27222	42.8864
71.29211	42.223
71.31201	42.25246
71.3319	38.63104
71.3518	39.01499
71.37169	39.04492
71.39158	36.59585
71.41148	38.6313
71.43137	37.26587
71.45127	35.42144
71.47116	35.78549
71.49106	34.33199

71.51095	34.19635
71.53085	34.22233
71.55074	32.77971
71.57064	31.27474
71.59053	31.08244
71.61043	31.06737
71.63032	30.55245
71.65021	29.07936
71.67011	29.67933
71.69	29.63363
71.7099	28.58287
71.72979	30.36039
71.74969	27.24223
71.76958	27.4211
71.78948	26.78242
71.80937	25.14388
71.82927	26.66175
71.84916	25.8204
71.86906	25.05211
71.88895	24.40898
71.90884	24.17745
71.92874	22.85754
71.94863	22.82944
71.96853	22.87441
71.98842	23.38307
72.00832	22.67314
72.02821	23.70814
72.04811	21.0558
72.068	22.19527
72.0879	21.15781
72.10779	21.83925
72.12768	19.19792
72.14758	19.6609
72.16747	20.19175
72.18737	17.48316
72.20726	19.68617
72.22716	18.10808
72.24705	19.2541
72.26695	17.91069
72.28684	17.10908
72.30674	18.37013
72.32663	15.65215
72.34653	17.61662
72.36642	15.34685
72.38631	16.35848
72.40621	15.99005
72.4261	14.49156
72.446	15.80571
72.46589	14.95855
72.48579	15.96049
72.50568	14.53549
72.52558	13.82418
72.54547	13.51927
72.56537	13.99054
72.58526	15.11299
72.60516	13.93351
72.62505	13.20729
72.64494	12.99164
72.66484	12.50529
72.68473	12.5295
72.70463	11.84552
72.72452	12.6044

72.74442	12.69154
72.76431	11.0809
72.78421	12.30374
72.8041	10.44339
72.824	10.03631
72.84389	9.99915
72.86379	10.41006
72.88368	9.6961
72.90357	10.56041
72.92347	9.43528
72.94336	9.36757
72.96326	9.50834
72.98315	9.65446
73.00305	10.07676
73.02294	9.32211
73.04284	8.03635
73.06273	8.00073
73.08263	10.59546
73.10252	7.16949
73.12242	7.99886
73.14231	7.719
73.1622	7.22053
73.1821	7.44094
73.20199	5.59378
73.22189	7.74155
73.24178	6.98841
73.26168	7.18853
73.28157	7.8315
73.30147	6.06314
73.32136	6.75325
73.34126	6.95912
73.36115	7.54013
73.38104	6.50147
73.40094	7.08796
73.42083	6.92978
73.44073	6.8759
73.46062	5.32737
73.48052	7.06021
73.50041	6.56403
73.52031	6.55756
73.5402	6.53559
73.5601	6.80022
73.57999	5.95039
73.59989	7.09549
73.61978	5.40218
73.63967	5.51629
73.65957	6.00553
73.67946	6.03656
73.69936	5.16148
73.71925	4.42194
73.73915	3.55233
73.75904	4.2245
73.77894	4.06869
73.79883	3.86092
73.81873	4.56994
73.83862	3.0343
73.85852	3.52483
73.87841	3.96861
73.8983	2.34481
73.9182	2.26802
73.93809	4.73301
73.95799	3.02105

73.97788	3.35609
73.99778	3.04542
74.01767	3.44842
74.03757	3.11717
74.05746	2.49438
74.07736	2.13213
74.09725	2.79605
74.11715	2.24655
74.13704	2.41071
74.15693	1.83021
74.17683	1.15087
74.19672	2.25291
74.21662	2.34986
74.23651	1.70214
74.25641	2.36704
74.2763	1.59457
74.2962	2.067
74.31609	0.64373
74.33599	1.7987
74.35588	1.28713
74.37578	1.24443
74.39567	2.55601
74.41556	0.87813
74.43546	0.63787
74.45535	1.80398
74.47525	2.55875
74.49514	1.03239
74.51504	0.48011
74.53493	1.98002
74.55483	0.90714
74.57472	1.70417
74.59462	0.57944
74.61451	0.32983
74.6344	0.92409
74.6543	1.63825
74.67419	0.63378
74.69409	0.35338
74.71398	-0.18211
74.73388	-0.33206
74.75377	0.6483
74.77367	0.19649
74.79356	-0.43229
74.81346	-0.61824
74.83335	1.35737
74.85325	-0.21898
74.87314	-1.04001
74.89303	0.7849
74.91293	-0.11924
74.93282	-0.30973
74.95272	-0.19281
74.97261	-0.47153
74.99251	-0.1825
75.0124	0.9347
75.0323	-0.25535
75.05219	-0.51305
75.07209	0.75532
75.09198	0.87271
75.11188	0.63597
75.13177	-0.01739
75.15166	0.79804
75.17156	0.36352
75.19145	0.42904

75.21135	0.67169
75.23124	0.15918
75.25114	0.55816
75.27103	1.53011
75.29093	0.27293
75.31082	2.04705
75.33072	1.23267
75.35061	0.7777
75.37051	2.06236
75.3904	2.1804
75.41029	0.85577
75.43019	2.36973
75.45008	2.45664
75.46998	1.24672
75.48987	1.89622
75.50977	1.22284
75.52966	0.63283
75.54956	2.74078
75.56945	2.59878
75.58935	2.63389
75.60924	2.66384
75.62914	2.21467
75.64903	1.58324
75.66892	1.47268
75.68882	1.99237
75.70871	1.1996
75.72861	2.32875
75.7485	0.72355
75.7684	0.71215
75.78829	0.75808
75.80819	1.18425
75.82808	0.24588
75.84798	1.32838
75.86787	1.12967
75.88776	1.06121
75.90766	0.03965
75.92755	0.70043
75.94745	0.10603
75.96734	-0.00916
75.98724	0.03194
76.00713	-0.1561
76.02703	0.93194
76.04692	0.64502
76.06682	1.13418
76.08671	1.32649
76.10661	0.82613
76.1265	-0.23148
76.14639	0.69011
76.16629	0.69507
76.18618	0.73131
76.20608	0.73113
76.22597	0.4341
76.24587	1.53816
76.26576	2.31933
76.28566	1.23074
76.30555	2.04844
76.32545	3.17346
76.34534	2.88705
76.36524	4.63714
76.38513	4.50705
76.40502	5.26762
76.42492	6.68968

76.44481	6.04927
76.46471	7.59639
76.4846	7.09667
76.5045	8.14907
76.52439	8.27441
76.54429	9.54041
76.56418	8.80644
76.58408	8.43708
76.60397	8.47401
76.62387	9.32346
76.64376	9.00107
76.66365	8.32975
76.68355	7.99701
76.70344	7.76325
76.72334	7.39931
76.74323	6.67603
76.76313	6.33298
76.78302	5.79725
76.80292	5.25635
76.82281	4.2936
76.84271	1.59129
76.8626	2.83172
76.8825	3.34301
76.90239	1.75536
76.92228	1.42817
76.94218	1.50725
76.96207	0.62281
76.98197	1.07694
77.00186	0.42173
77.02176	0.22487
77.04165	1.26763
77.06155	-0.50209
77.08144	0.82196
77.10134	0.50021
77.12123	0.3764
77.14113	0.89324
77.16102	-0.0899
77.18091	1.08845
77.20081	0.54807
77.2207	-0.29436
77.2406	-0.2149
77.26049	0
77.28039	0.00398
77.30028	0.27873
77.32018	0.5378
77.34007	-0.3285
77.35997	-0.04434
77.37986	-0.26592
77.39975	-0.48276
77.41965	-0.15836
77.43954	0.72817
77.45944	0.4008
77.47933	1.7972
77.49923	0.93312
77.51912	0.26691
77.53902	0.29334
77.55891	0.25201
77.57881	0.84603
77.5987	1.20042
77.6186	0.76828
77.63849	1.19026
77.65838	-0.02891

77.67828	0.12057
77.69817	0.58663
77.71807	2.03077
77.73796	1.11966
77.75786	0.5096
77.77775	0.22664
77.79765	-0.07294
77.81754	-0.17037
77.83744	-1.07604
77.85733	-0.06076
77.87723	0.46924
77.89712	-0.49645
77.91701	0.05262
77.93691	0.47064
77.9568	0.63783
77.9767	0.18962
77.99659	0.34999
78.01649	0.17625
78.03638	0.50696
78.05628	-0.36619
78.07617	0.86929
78.09607	0.1249
78.11596	-0.83373
78.13586	0.24894
78.15575	0.23262
78.17564	0.47669
78.19554	-0.07615
78.21543	0.91264
78.23533	0.16703
78.25522	0.07242
78.27512	-0.3868
78.29501	0.73207
78.31491	-0.44597
78.3348	0.31867
78.3547	0.15099
78.37459	0.44681
78.39449	0.45093
78.41438	1.02273
78.43427	0.70387
78.45417	1.60373
78.47406	0.79523
78.49396	-0.01331
78.51385	0.13124
78.53375	0.67159
78.55364	-0.03809
78.57354	0.01782
78.59343	0.55808
78.61333	0.69726
78.63322	-0.18964
78.65311	1.44427
78.67301	0.02606
78.6929	-0.65781
78.7128	0.41871
78.73269	1.22436
78.75259	0.4831
78.77248	0.7991
78.79238	1.20361
78.81227	0.75913
78.83217	0.65836
78.85206	-0.17681
78.87196	0.61819
78.89185	0.15862

78.91174	0.38078
78.93164	0.23773
78.95153	1.93777
78.97143	0.52246
78.99132	0.64278
79.01122	-0.6545
79.03111	1.28674
79.05101	0.39347
79.0709	1.04571
79.0908	-0.25672
79.11069	-0.26406
79.13059	1.05984
79.15048	-0.07181
79.17037	0.60093
79.19027	1.41083
79.21016	1.27904
79.23006	0.35043
79.24995	1.24871
79.26985	1.01304
79.28974	0.52736
79.30964	0.22397
79.32953	0.96225
79.34943	1.11199
79.36932	0.38152
79.38922	0
79.40911	-1.42E-14
